# Keto-Enol Tautomerism in Passerini and Ugi Adducts

**DOI:** 10.3390/molecules26040919

**Published:** 2021-02-09

**Authors:** Pablo Pertejo, Andrea Sancho-Medina, Tomás Hermosilla, Beatriz González-Saiz, Javier Gómez-Ayuso, Roberto Quesada, Daniel Moreno, Israel Carreira-Barral, María García-Valverde

**Affiliations:** 1 Departamento de Química, Facultad de Ciencias, Universidad de Burgos, 09001 Burgos, Spain; ppf0003@alu.ubu.es (P.P.); asmedina@ubu.es (A.S.-M.); thj0001@alu.ubu.es (T.H.); beatrizgs@ubu.es (B.G.-S.); jga0108@alu.ubu.es (J.G.-A.); rquesada@ubu.es (R.Q.); 2 Departamento de Didáctica de las Matemáticas y las Ciencias Experimentales, Facultad de Educación, Universidad Internacional de La Rioja, 26006 Logroño, Spain; daniel.moreno@unir.net

**Keywords:** Keto-enol tautomerism, Passerini adduct, Ugi adduct, lactone, lactame, 2-hydroxyglutaric acid

## Abstract

The use of arylglyoxal as starting material in Passerini and Ugi reactions affords β-ketoamides. This has allowed to study keto-enol tautomerism in these systems and assess the way in which the presence of acyloxy or aminoacyl groups bound to the C2 position affects such tautomerism, and to investigate the reactivity of both the enol and carbonyl forms. In this work we also prove the versatility of the Passerini reaction, since depending on the conditions to which the corresponding adducts are subjected different products of synthetic interest can be obtained.

## 1. Introduction

**Figure 1:**
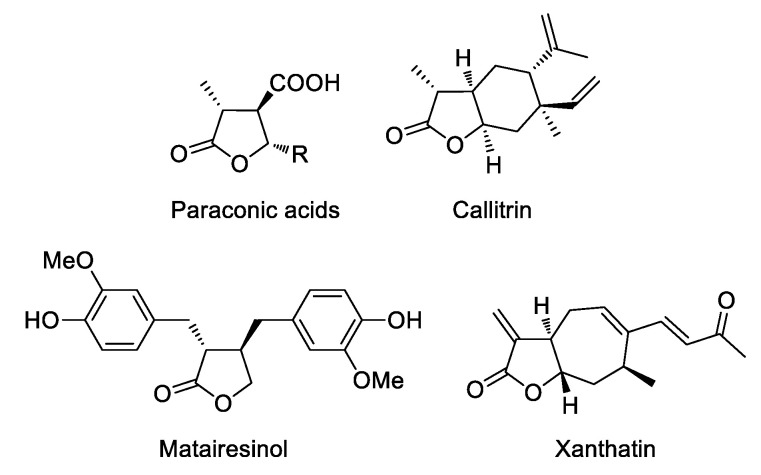
γ-Lactones present in natural products.

Keto-enol tautomerism, especially in 1,3-dicarbonyl systems, has been widely studied. Most of the research conducted in this field has focused on determining the influence of structural factors (aromaticity, conjugation, sterical effects, hydrogen bonds, etc.) or external effects (temperature, solvent, concentration, etc.) on tautomeric equilibria [1,2,3,4], NMR being the most useful tool to evaluate it [5,6]. Although one of the most important aspects of keto-enol tautomerism is the different reactivity of tautomers, it is not easy to find examples in the literature addressing this issue in structurally similar compounds. Given this, we decided to investigate keto-enol tautomerism in β-ketoamides synthesized by Passerini [7,8] and Ugi reactions [9], from their synthesis, identification and analysis, to their different reactivity and use in the construction of different substituted systems. The final purpose of this work has been the use of the corresponding adducts as intermediates in the synthesis of highly functionalized γ-lactames and mainly γ-lactones, and of novel 2-hydroxyglutaric acid derivatives, because of their wide range of biological activity and their use as therapeutic agents (Figure 1) [10,11].

## 2. Discussion of Results

### 2.1. Keto-Enol Tautomerism in Passerini and Ugi Adducts

Firstly, we analyzed the tautomeric equilibrium from both the theoretical and experimental point of view. We synthesized β-ketoamides substituted at C2 with acyloxy and aminoacyl groups, employing phenylglyoxal in the Passerini [12] and Ugi [13] reactions.

**Scheme 1:**
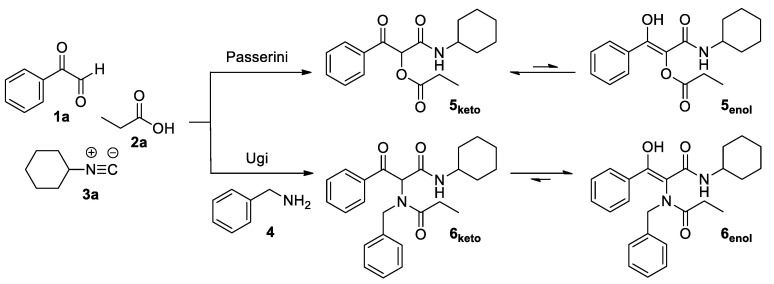
Synthesis of β-ketoamides by Passerini and Ugi reactions.

In order to carry out the Passerini reaction, phenylglyoxal (**1a**), propionic acid (**2a**) and cyclohexyl isocyanide (**3a**) were used, with benzylamine (**4**) acting as the fourth component in the Ugi reaction. These processes afforded Passerini and Ugi adducts **5** and **6**, respectively (Scheme 1), which after their isolation were characterized by means of NMR spectroscopy. The ^1^H NMR spectrum of Passerini adduct **5** exhibits a singlet at 6.3 ppm, corresponding to the proton linked to the C2 position of the keto tautomer, while the signal attributable to the enol hydroxylic proton is not observed, indicating that the Passerini adduct is in its keto form. However, the ^1^H NMR spectrum of Ugi adduct **6** displays a deshielded signal (15.3 ppm), due to the proton of the enol group, and the singlet that would be attributed to the proton at the C2 position of the keto tautomer is not shown. These data confirm that the Ugi adduct is found exclusively in its enol form. (NMR data and additional crystallographic data are presented in the Supplementary Materials).

**Figure 2:**
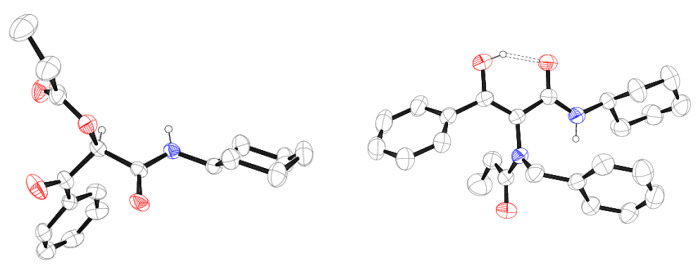
X-ray molecular structures of Passerini **5** (**left**) and Ugi **6** (**right**) adducts. The ORTEP plot is at the 30% probability level. Hydrogen atoms, except those of the NH fragments, that of the OH group in **6** and that linked to C2 in **5**, have been omitted for the sake of simplicity.

Single-crystal X-ray diffraction analyses of compounds **5** and **6** show that the major tautomer is different in each case (Figure 2), in agreement with solution studies. Interestingly, although it has been described that both enol and keto tautomers are able to establish intramolecular hydrogen-bonding interactions [14,15], no intramolecular hydrogen bond between the NH fragment of the amide group and the carbonyl oxygen (N-H⋯O) is observed for the keto tautomer of Passerini adduct **5**, neither in the solid state nor in solution. Indeed, the chemical shift of the signal due to the NH proton observed in its ^1^H NMR spectrum is too low (6.2 ppm) to participate in this bond.

**Figure 3:**
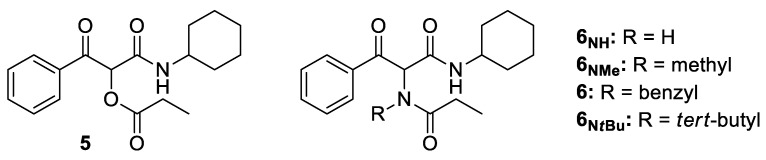
Keto forms of the studied structures.

Different studies dealing with keto-enol tautomerism in β-ketoamides concluded that the presence of electron-donating groups at their C2 position weakens the intramolecular hydrogen bond involving the OH fragment of the enol tautomer, favoring the carbonyl form; nevertheless, electron-withdrawing groups strengthen such a hydrogen bond and therefore shift the equilibrium towards the enol tautomer [14]. Considering these facts, in the present case we expected the shift of the tautomeric equilibrium towards the enol form in both the Passerini and Ugi adducts. Intrigued by our experimental results, we tried the synthesis of a third derivative, the non-substituted Ugi adduct **6_NH_**, similar to **6** but derived from ammonia, in order to determine both the electronic and steric effect of the C2 substituent on the equilibrium. Although the synthesis of this compound was not successful, we decided to include it in the theoretical study with the purpose of establishing the role of the heteroatom attached to the C2 position in the tautomeric equilibrium and subsequently the steric effect due to the *N*-benzyl substituent. So, initially we conducted DFT quantum chemical calculations for adducts **5**, **6_NH_** and **6**, using Gaussian 09 [16] (Figure 3). The geometries of all species were fully optimized at the B3LYP/6-31G** level. The environmental effects were taken into account by the polarizable continuum model (PCM) using the integral equation formalism variant (IEFPCM).

**Scheme 2:**
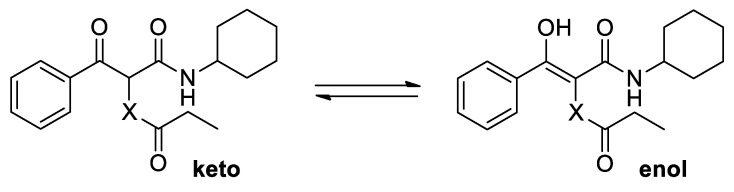
Tautomeric equilibrium in Passerini and Ugi adducts.

The performed calculations were in agreement with the experimental data for adducts **5** and **6**. Thus, for Passerini adduct **5** (X: O) the keto form was the most stable tautomer, whereas the enol form was the thermodynamically favorable tautomer in the case of Ugi adduct **6** (X: NBn); the equilibrium constants (in chloroform) for the conversion of the keto form into the enol one amount to 1.4 × 10^−3^ and 4.0 × 10^4^, respectively (Scheme 2; Entries 1 and 4, Table 1). This difference seems to be attributable more to steric than to electronic effects. Indeed, the calculated equilibrium constant for non-substituted Ugi adduct **6_NH_** (X: NH), although higher than the equilibrium constant for Passerini adduct **5** (X: O) (Entries 1 and 2, Table 1), remains very low, while the introduction of a benzyl substituent on the nitrogen attached to the C2 position shifts the equilibrium to the enol form. In order to increase the scope of this study, the theoretical analysis was extended to other *N*-substituted Ugi adducts, namely *N*-methyl (**6_NMe_**, X: NMe) and *N*-*tert*-butyl (**6_N*t*Bu_**, X: *N^t^*Bu) (Scheme 2; Entries 3 and 5, Table 1). Overall, these calculations show that the most favorable tautomer for all *N*-substituted Ugi adducts is the enol one. In this sense, a correlation between steric hindrance and the calculated equilibrium constants is noticed, since as the former increases, the latter also rises. These results support the importance of the steric factor in the tautomeric equilibrium.

As mentioned above, electron-withdrawing groups at C2 should strengthen the intramolecular hydrogen bond involving the OH fragment of the enol group, thus stabilizing the enol tautomer [14]. Due to this, we calculated charge distribution in adducts **5** and **6_NH_** by NBO analysis, in order to determine the character of acyloxy and aminoacyl groups as substituents in the β-ketoamides from the electronic point of view. This analysis revealed a more negative carbon adjacent to the aminoacyl group in **6_NH_**, −0.22*e* and −0.10*e* for the keto and enol forms, respectively, than for the carbon contiguous to the acyloxy group, with values of −0.07*e* and 0.08*e*. Therefore, the latter has a higher electron-withdrawing character, and therefore the enol tautomer should be more favored in Passerini adduct **5** than in non-substituted Ugi adduct **6_NH_**, in contrast to the theoretical results (Entries 1 and 2, Table 1). Nonetheless, the keto form would be the major tautomer in both cases.

Bearing these results in mind, the difference in the tautomeric equilibrium for Passerini **5** and Ugi **6** adducts appears to be due to steric reasons more than to electronic factors. Thus, when the benzyl group is attached to the nitrogen atom in the aminoacyl substituent of the Ugi adduct, the shift of the equilibrium to the enol form would be explained by the reduction of steric repulsion. In this way, the aminoacyl substituent at C2 would be located in a perpendicular fashion with respect to the enol double bond, allowing the benzyl and propionyl groups to get away from the bulky cyclohexyl and phenyl substituents in the enol tautomer of Ugi adduct **6**. These results are supported by the X-ray molecular structure (Figure 2) and the ^1^H NMR spectrum of Ugi adduct **6**. This spectrum displays signals due to diastereotopic protons, regardless of the absence of a stereogenic centre at C2 in this enol form: an AB spin system assignable to the benzyl protons and an ABX_3_ spin system corresponding to the ethyl group. This proves the existence of a stereogenic element in the molecule, a chiral axis, a consequence of the restriction of the spin around the C_enol_-N bond, thus leading to atropisomers (Figure 2) [17].

### 2.2. Reactivity of Keto and Enol Tautomers

**Scheme 3:**
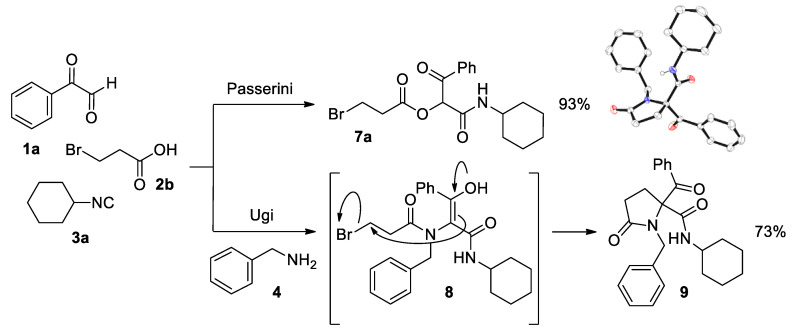
Passerini and Ugi reactions using phenylglyoxal and 3-bromopropionic acid and X-ray molecular structure of lactame **9**. The ORTEP plot is at the 30% probability level. Hydrogen atoms, except that of the NH fragment, have been omitted for the sake of simplicity.

An important aspect of keto and enol tautomers is their different reactivity. Indeed, the keto form is characterized by the electrophilicity of the carbonyl carbon and the acidity of the α positions, while the enol tautomer is distinguished by the nucleophilicity of the α carbon and the acidity of its OH group. Considering this reactivity and the different tautomers observed for the Passerini and Ugi adducts derived from phenylglyoxal, keto **5_keto_** and enol **6_enol_** (Scheme 1), we envisaged the possibility of studying their different reactivity by introducing an electrophilic position in the carboxylic component. Thus, propionic acid (**2a**) was replaced by 3-bromopropionic acid (**2b**), keeping the remaining reagents. The reactions would afford either 3-bromopropanoate **7a** (Passerini adduct) or 2-bromopropionamide **8** (Ugi adduct) (Scheme 3).

As expected, the results were rather different because of the preferred tautomer. In this way, although Passerini adduct **7a** was obtained with a high yield, Ugi adduct **8** could not be isolated, since a spontaneous cyclization took place yielding γ-lactame **9** (Scheme 3). This is the result of an intramolecular nucleophilic substitution, because of the nucleophilic character of the α carbon on the enol tautomer, as previously reported [18,19].

Then, we tried the cyclization of the Passerini adduct to the γ-lactone, based on the acidity of the α position in the keto tautomer. Consequently, this adduct was subjected to different alkaline media. Although in many cases the final product was not the expected γ-lactone, in most of them the isolated compounds were interesting intermediates obtained in a quantitative way, which proves the versatility and the synthetic interest of Passerini adducts.

**Scheme 4:**
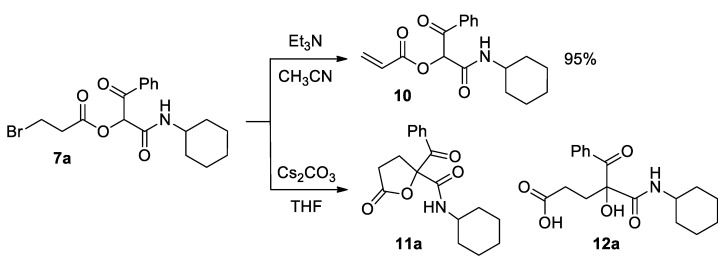
Passerini adduct **7a** treated in basic media.

Use of potassium hydroxide in methanol led to a complex mixture of products, which could not be identified, likely formed from the saponification product. Then, a solution of triethylamine in acetonitrile was used; this afforded a new compound, not the γ-lactone but acrylate **10**, resulting from elimination of hydrogen bromide (Scheme 4). This was confirmed by the ^1^H NMR spectrum of the product, which exhibited the typical signals of a monosubstituted olefin. In light of these results, the cyclization reaction was attempted employing cesium carbonate in boiling THF, turning out that the water content in the solvent was crucial. Thus, when dry THF was used, Passerini adduct **7a** was recovered, but when wet THF was employed a mixture of two compounds was obtained, γ-lactone **11a** and 4-benzoyl-5-(cyclohexylamino)-4-hydroxy-5-oxopentanoic acid **12a** (Scheme 4), the latter resulting from the lactone’s opening, in a mechanism similar to that of Favorskii’s rearrangement. No other products were identified.

**Scheme 5:**
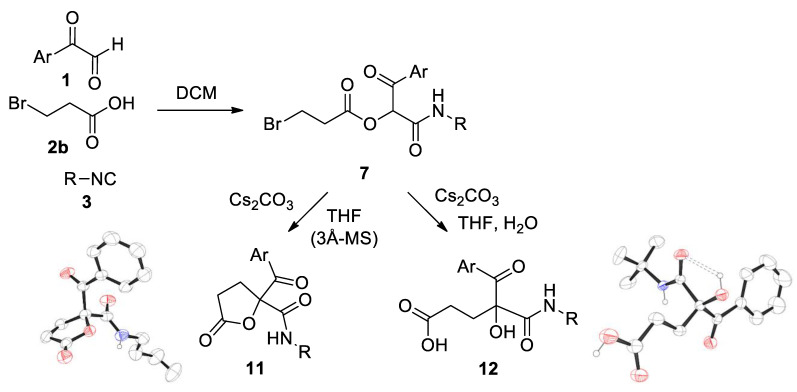
Synthesis of γ-lactones **11** and 2-hydroxyglutaric acid derivatives **12** from 3-bromopropionic acid. X-ray molecular structures of **11b** (left) and **12c** (right) are also displayed. The ORTEP plot is at the 30% probability level. Hydrogen atoms, except those of NH and OH fragments, have been omitted for the sake of simplicity.

In view of these results we tried to optimize the synthesis of each of these derivatives. Thus, we used cesium carbonate in THF, varying the amount of water in the medium. Quantitative synthesis of γ-lactone **11a** was achieved when THF was kept on 3 Å molecular sieves for three minutes before its use, whereas quantitative synthesis of glutaric acid derivative **12a** was reached when some drops of water were added to the reaction mixture. Under these conditions we synthesized a variety of γ-lactones **11** and 2-hydroxyglutaric acid derivatives **12**, from different arylglyoxals **1** and isocyanides **3**, with good yields (Scheme 5, Table 2).

**Scheme 6:**
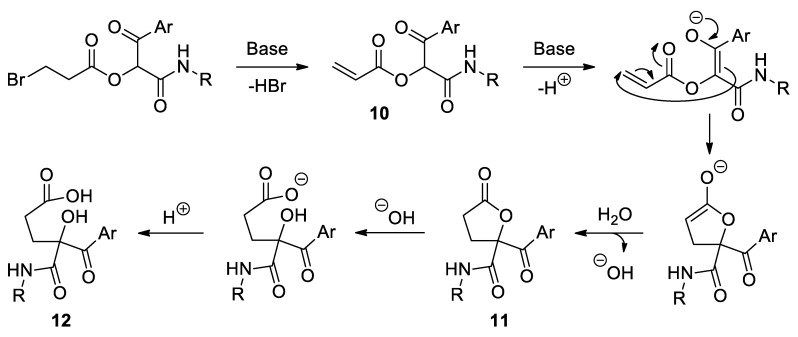
Proposed mechanism in the synthesis of Passerini derivatives.

The study of the reaction progress over time by NMR spectroscopy allowed to conclude that in the course of the reaction elimination product **10** was generated and eventually developed into the cyclization product. This seems to support the fact that the cyclization reaction does not take place through an intramolecular nucleophilic substitution, but via an elimination mechanism followed by a nucleophilic conjugate addition of the enolate to the α,β-unsaturated system (Scheme 6). This was confirmed by treating the elimination product, isolated from the reaction of the Passerini adduct with triethylamine, with cesium carbonate in THF, which led again to γ-lactone **11**.

**Scheme 7:**
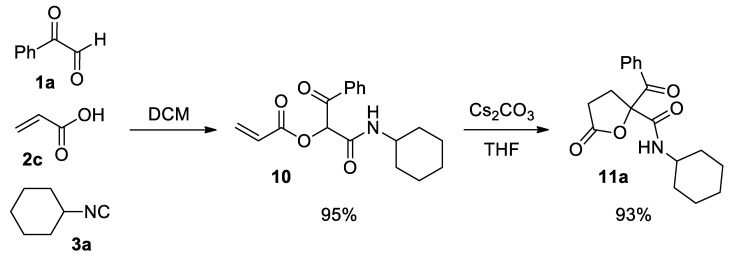
Synthesis of γ-lactones from acrylic acid.

This result opened the door to exploring the synthesis of γ-lactones **11** through a new synthetic route, with a complete atom economy, as acrylate **10** could be synthesized directly from acrylic acid **2c** in combination with phenylglyoxal **1a** and cyclohexyl isocyanide **3a** through the Passerini reaction. In this way, by a two-step sequence and with a complete atom economy, we synthesized γ-lactone **11a** (Scheme 7).

**Scheme 8:**
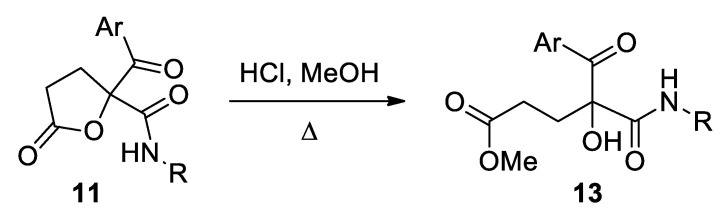
γ-Lactone opening in methanol and acidic medium.

Finally, in order to increase the synthetic potential of the Passerini reaction, hydrolysis of γ-lactones **11** in acidic medium was attempted. Thus, the corresponding γ-lactone was dissolved in methanol and subjected to acidic conditions (HCl 0.5 M, 4 equiv.), and the mixture was refluxed for 2–3 h. The hydrolysis products were reached, as methyl esters **13**, resulting from a transesterification reaction (Scheme 8, Table 3). γ-Lactone opening can also be performed in alkaline conditions (catalytic sodium methoxide in methanol) at room temperature. However, in this case the process must be conducted in dry methanol under nitrogen atmosphere, since the reaction mixture is moisture-sensitive. Thus, besides transesterification compounds **13**, in the presence of wetness the formation of saponification derivatives **12** as secondary products is observed.

In summary, it is easy to see the great versatility of the Passerini reaction when employing doubly functionalized systems as starting materials. The use of arylglyoxals and 3-bromopropionic acid or acrylic acid, besides an isocyanide, leads to Passserini adducts, which, after being treated with commercially available, inexpensive and ecofriendly reagents, drive to highly functionalized systems, with a clear interest from the synthetic point of view.

## 3. Materials and Methods

### 3.1. General Methods

Melting points were not corrected. ^1^H, ^19^F and ^13^C NMR spectra were recorded in CDCl_3_, CD_3_CN or DMSO-*d*_6_ on Varian Mercury Plus 300 (at 300 and 75 MHz, respectively) or Varian Unity Inova 400 (at 400 and 100 MHz, respectively) spectrometers (Varian, Inc., Palo Alto, United States of America); DEPT-135 experiments were performed to assign carbon-13 signals. Chemical shifts are reported in parts per million with respect to residual solvent protons and coupling constants in hertz. High-resolution mass spectra were recorded on a 6545 Q-TOF Agilent LC-MS mass spectrometer (electrospray, positive ionization mode, ESI(+); Agilent Technologies, Inc., Santa Clara, United States of America). Single-crystal X-ray diffraction analyses were conducted on a Bruker D8 Venture diffractometer equipped with a Photon III area detector (Bruker Corporation, Billerica, United States of America)

### 3.2. Synthesis and Characterization

#### 3.2.1. Passerini Adducts

Method A: The corresponding arylglyoxal hydrate **1** (1 mmol) was dissolved in dichloromethane (10 mL), after which the corresponding acid **2a-c** (1 mmol) and the corresponding isocyanide **3** (1 mmol) were added. The reaction mixture was stirred at room temperature for 24 h. A hydrochloric acid aqueous solution (1 mL) was added to neutralize the unreacted isocyanide and the mixture was washed with a sodium carbonate aqueous solution (1 × 50 mL). The organic phase was dried over anhydrous sodium sulfate, filtered and concentrated to dryness. Slow evaporation of a solution of compound **5** in a 1:1 chloroform-hexane mixture provided colorless single crystals, suitable for X-ray diffraction analysis.

Method B: compound **10** was also prepared following a different procedure: Passerini adduct **7a** (1 mmol) was dissolved in acetonitrile (10 mL) and triethylamine (1.1 mmol) was added. The reaction mixture was stirred at room temperature for 24 h and, after that, concentrated to dryness. The crude was dissolved in dichloromethane (20 mL) and this solution washed with a hydrochloric acid aqueous solution (1 × 50 mL). The organic phase was dried over anhydrous sodium sulfate, filtered and concentrated to dryness. The residue was purified by column chromatography, employing silica gel as a stationary phase and a 4:1 hexane-ethyl acetate mixture as an eluent.

*1-(Cyclohexylamino)-1,3-dioxo-3-phenylpropan-2-yl propionate* (**5**). White solid. Yield: 82%. M. p. 110–111 °C. ^1^H NMR (300 MHz, CDCl_3_) δ (ppm): 8.17–8.11 (m, 2H), 7.63–7.57 (m, 1H), 7.51–7.45 (m, 2H), 6.29 (s, 1H), 6.21 (d, *J* = 7.7 Hz, 1H, NH), 3.79–3.67 (m, 1H), 2.55 (qd, *J* = 15.2, 7.6 Hz, 1H), 2.54 (qd, *J* = 15.2, 7.6 Hz, 1H), 1.97–1.54 (m, 4H), 1.42–1.09 (m, 6H), 1.20 (t, *J* = 7.5 Hz, 3H). ^13^C NMR {DEPT-135} (75 MHz, CDCl_3_) δ (ppm): 191.9 (C_q_), 172.2 (C_q_), 162.7 (C_q_), 134.4 (C_q_), 133.8 (CH_Ar_), 129.5 (CH_Ar_), 128.4 (CH_Ar_), 76.1 (CH), 48.5 (CH), 32.5 (CH_2_), 32.4 (CH_2_), 27.0 (CH_2_), 25.2 (CH_2_), 24.6 (CH_2_), 24.6 (CH_2_), 8.7 (CH_3_). HRMS (ESI): calculated for C_18_H_24_NO_4_ [MH^+^] 318.1700, found 318.1703.

*1-(Cyclohexylamino)-1,3-dioxo-3-phenylpropan-2-yl 3-bromopropanoate* (**7a**). Pale yellow solid. Yield: 93%. M. p. 114–115 °C. ^1^H NMR (300 MHz, CDCl_3_) δ (ppm): 8.16–8.09 (m, 2H), 7.63–7.57 (m, 1H), 7.52–7.45 (m, 2H), 6.36 (s, 1H), 6.30 (d, *J* = 7.7 Hz, 1H, NH), 3.78–3.66 (m, 1H), 3.61 (t, *J* = 6.8 Hz, 2H), 3.14 (t, *J* = 6.6 Hz, 2H), 1.98–1.06 (m, 10H). ^13^C NMR {DEPT-135} (75 MHz, CDCl_3_) δ (ppm): 191.5 (C_q_), 168.6 (C_q_), 162.4 (C_q_), 134.4 (C_q_), 134.3 (CH_Ar_), 129.8 (CH_Ar_), 128.7 (CH_Ar_), 76.6 (CH), 48.9 (CH), 37.4 (CH_2_), 32.8 (CH_2_), 32.7 (CH_2_), 25.4 (CH_2_), 25.2 (CH_2_), 24.8 (CH_2_), 24.8 (CH_2_). HRMS (ESI): calculated for C_18_H_23_BrNO_4_ [MH^+^] 396.0805, found 396.0805.

*1-(Butylamino)-1,3-dioxo-3-phenylpropan-2-yl 3-bromopropanoate* (**7b**). Yellow oil. Yield: 84%. ^1^H NMR (300 MHz, CDCl_3_) δ (ppm): 8.10–8.04 (m, 2H), 7.57–7.52 (m, 1H), 7.46–7.38 (m, 2H), 6.66 (t, *J* = 5.5 Hz, 1H, NH), 6.33 (s, 1H), 3.56–3.50 (m, 2H), 3.27–3.09 (m, 2H), 3.08–3.02 (m, 2H), 1.45–1.36 (m, 2H), 1.29–1.17 (m, 2H), 0.82 (t, *J* = 7.3 Hz, 3H). ^13^C NMR {DEPT-135} (75 MHz, CDCl_3_) δ (ppm): 191.3 (C_q_), 168.6 (C_q_), 163.2 (C_q_), 134.2 (C_q_), 134.1 (CH_Ar_), 129.5 (CH_Ar_), 128.5 (CH_Ar_), 76.3 (CH), 39.2 (CH_2_), 37.1 (CH_2_), 31.1 (CH_2_), 25.2 (CH_2_), 19.8 (CH_2_), 13.5 (CH_3_). HRMS (ESI): calculated for C_16_H_21_BrNO_4_ [MH^+^] 370.0648, found 370.0651.

*1-(tert-Butylamino)-1,3-dioxo-3-phenylpropan-2-yl 3-bromopropanoate* (**7c**). Yellow oil. Yield: 87%. ^1^H NMR (300 MHz, CDCl_3_) δ (ppm): 8.10–8.04 (m, 2H), 7.57–7.52 (m, 1H), 7.46–7.40 (m, 2H), 6.25 (s_b_, 1H, NH), 6.23 (s, 1H), 3.55 (t, *J* = 6.5 Hz, 1H), 3.54 (t, *J* = 6.5 Hz, 1H), 3.06 (t, *J* = 6.5 Hz, 2H), 1.29 (s, 9H). ^13^C NMR {DEPT-135} (75 MHz, CDCl_3_) δ (ppm): 191.7 (C_q_), 168.5 (C_q_), 162.4 (C_q_), 134.4 (C_q_), 134.1 (CH_Ar_), 129.6 (CH_Ar_), 128.6 (CH_Ar_), 76.4 (CH), 52.0 (C_q_), 37.2 (CH_2_), 28.4 (CH_3_), 25.2 (CH_2_). HRMS (ESI): calculated for C_16_H_21_BrNO_4_ [MH^+^] 370.0648, found 370.0650.

*1-(Cyclohexylamino)-3-(4-fluorophenyl)-1,3-dioxopropan-2-yl 3-bromopropanoate* (**7d**). Yellow oil. Yield: 86%. ^1^H NMR (300 MHz, CDCl_3_) δ (ppm): 8.22–8.15 (m, 2H), 7.20–7.12 (m, 2H), 6.35–6.28 (m, 1H, NH), 6.31 (s, 1H), 3.78–3.66 (m, 1H), 3.62 (t, *J* = 6.7 Hz, 1H), 3.61 (t, *J* = 6.7 Hz, 1H), 3.14 (t, *J* = 6.7 Hz, 2H), 1.97–1.08 (m, 10H). ^13^C NMR {DEPT-135} (75 MHz, CDCl_3_) δ (ppm): 189.6 (C_q_), 168.5 (C_q_), 166.2 (C_q_, d, ^1^*J* = 257 Hz), 162.2 (C_q_), 132.5 (CH_Ar_, d, ^3^*J* = 9.6 Hz), 130.6 (C_q_, d, ^4^*J* = 2.8 Hz), 115.7 (CH_Ar_, d, ^2^*J* = 22 Hz), 76.2 (CH), 48.7 (CH), 37.1 (CH_2_), 32.5 (CH_2_), 32.4 (CH_2_), 25.2 (CH_2_), 24.6 (CH_2_), 24.6 (CH_2_). ^19^F NMR (300 MHz, CDCl_3_) δ: −102.8 (tt, *J* = 8.3, 5.4 Hz). HRMS (ESI): calculated for C_18_H_22_BrFNO_4_ [MH^+^] 414.0711, found 414.0714.

*1-(Cyclohexylamino)-1,3-dioxo-3-phenylpropan-2-yl acrylate* (**10**). Pink oil. Yield: 95% (by both method A and B). ^1^H NMR (300 MHz, CDCl_3_) δ (ppm): 8.18–8.13 (m, 2H), 7.64–7.57 (m, 1H), 7.52–7.46 (m, 2H), 6.53 (dd, *J* = 17.3, 1.3 Hz, 1H), 6.37 (s, 1H), 6.30 (dd, *J* = 17.3, 10.4 Hz, 1H), 6.23 (s_b_, 1H, NH), 5.99 (dd, *J* = 10.4, 1.3 Hz, 1H), 3.80–3.68 (m, 1H), 1.98–1.07 (m, 10H). ^13^C NMR {DEPT-135} (75 MHz, CDCl_3_) δ (ppm): 191.8 (C_q_), 164.0 (C_q_), 162.7 (C_q_), 134.5 (C_q_), 134.2 (CH_Ar_), 133.4 (CH_2_), 129.8 (CH_Ar_), 128.7 (CH_Ar_), 126.8 (CH), 76.4 (CH), 48.8 (CH), 32.8 (CH_2_), 32.7 (CH_2_), 25.4 (CH_2_), 24.8 (CH_2_), 24.8 (CH_2_). HRMS (ESI): calculated for C_18_H_22_NO_4_ [MH^+^] 316.1543, found 316.1549.

#### 3.2.2. Ugi Adducts/Lactames

Phenylglyoxal hydrate **1a** (1 mmol) was dissolved in methanol (10 mL), after which benzylamine **4** (1 mmol), the corresponding acid **2a-b** (1 mmol) and cyclohexyl isocyanide **3a** (1 mmol) were added. The reaction mixture was stirred at room temperature for 24 h. The solvent was removed under reduced pressure and the residue dissolved in dichloromethane (20 mL). This solution was washed with a sodium hydroxide aqueous solution (2 × 50 mL) and with a hydrochloric acid aqueous solution (2 × 50 mL). The organic phase was dried over anhydrous sodium sulfate, filtered and concentrated to dryness. In the case of **6**, the product was purified by column chromatography, employing silica gel as a stationary phase and a 4:1 hexane-ethyl acetate mixture as an eluent. In the case of **9**, addition of ethyl acetate drove to the formation of a precipitate, which was isolated by vacuum filtration and dried in vacuo. Slow evaporation of a solution of **6** in diisopropyl ether and of a solution of **9** in acetone gave colorless single crystals, suitable for X-ray diffraction analysis.

*(E)-2-(N-Benzylpropionamido)-N-cyclohexyl-3-hydroxy-3-phenylacrylamide* (**6**). Brown solid. M. p. 90–92 °C. Yield: 82%. ^1^H NMR (300 MHz, CDCl_3_) δ (ppm): 15.26 (s, 1H, OH), 7.63–7.57 (m, 2H), 7.47–7.42 (m, 3H), 7.36–7.28 (m, 5H), 5.45 (d, *J* = 13.7 Hz, 1H), 5.02 (d, *J* = 8.1 Hz, 1H, NH), 3.52–3.40 (m, 1H), 3.31 (d, *J* = 13.7 Hz, 1H), 2.49 (dq, *J* = 16.7, 7.5 Hz, 1H), 2.27 (dq, *J* = 16.7, 7.5 Hz, 1H), 1.72–1.07 (m, 7H), 1.14 (t, *J* = 7.4 Hz, 3H), 1.07–0.71 (m, 2H), 0.36–0.23 (m, 1H). ^13^C NMR {DEPT-135} (75 MHz, CDCl_3_) δ (ppm): 176.6 (C_q_), 170.3 (C_q_), 167.9 (C_q_), 137.9 (C_q_), 133.6 (C_q_), 130.8 (CH_Ar_), 129.7 (CH_Ar_), 129.2 (CH_Ar_), 128.9 (CH_Ar_), 128.2 (CH_Ar_), 127.3 (CH_Ar_), 108.1 (C_q_), 52.9 (CH_2_), 48.1 (CH), 32.8 (CH_2_), 31.8 (CH_2_), 26.9 (CH_2_), 25.3 (CH_2_), 24.7 (CH_2_), 24.6 (CH_2_), 9.5 (CH_3_). HRMS (ESI): calculated for C_25_H_31_N_2_O_3_ [MH^+^] 407.2329, found 407.2333.

*2-Benzoyl-1-benzyl-N-cyclohexyl-5-oxopyrrolidine-2-carboxamide* (**9**). White solid. Yield: 73%. M. p. 184–186 °C. ^1^H NMR (300 MHz, CDCl_3_) δ (ppm): 7.83–7.78 (m, 2H), 7.60–7.53 (m, 1H), 7.46–7.38 (m, 2H), 7.24–7.11 (m, 5H), 5.83 (d, *J* = 7.8 Hz, 1H, NH), 5.04 (d, *J* = 16.2 Hz, 1H), 4.24 (d, *J* = 16.2 Hz, 1H), 3.42–3.28 (m, 1H), 2.92–2.81 (m, 1H), 2.79–2.67 (m, 1H), 2.56–2.40 (m, 2H), 1.57–0.62 (m, 10H). ^13^C NMR {DEPT-135} (75 MHz, CDCl_3_) δ (ppm): 196.9 (C_q_), 176.5 (C_q_), 167.2 (C_q_), 136.9 (C_q_), 133.9 (C_q_), 133.8 (CH_Ar_), 129.3 (CH_Ar_), 128.7 (CH_Ar_), 128.5 (CH_Ar_), 127.8 (CH_Ar_), 127.3 (CH_Ar_), 76.7 (C_q_), 49.2 (CH), 46.7 (CH_2_), 32.1 (CH_2_), 31.8 (CH_2_), 28.9 (CH_2_), 25.4 (CH_2_), 24.6 (CH_2_), 24.6 (CH_2_). HRMS (ESI): calculated for C_25_H_29_N_2_O_3_ [MH^+^] 405.2173, found 405.2176.

#### 3.2.3. Lactones

Method A: The corresponding Passerini adduct **7** (1 mmol) was dissolved in slightly wet (3 Å-MS) tetrahydrofuran (10 mL) and cesium carbonate (1.2 equiv.) was added. The reaction mixture was heated to reflux with stirring until TLC revealed the total consumption of the starting material (1–1.5 h). Subsequently, the unreacted salt was filtered off and the solvent of the filtrate removed under reduced pressure. The crude was dissolved in dichloromethane (20 mL) and the solution washed with water (2 × 20 mL). The organic phase was dried over anhydrous sodium sulfate, filtered and concentrated to dryness. The residue was purified by column chromatography, employing silica gel as a stationary phase and a 4:1 hexane-ethyl acetate mixture as an eluent. Hexane was added to the resulting oil and, in some cases, a precipitate was observed. This solid was isolated by vacuum filtration and dried in vacuo. Slow evaporation of a solution of compound **11b** in a 1:1 chloroform-hexane mixture provided colorless single crystals, suitable for X-ray diffraction analysis.

Method B: compound **11a** was also prepared starting from Passerini adduct **10**. Reaction conditions, as well as the work-up and purification steps, are similar to those described in the previous method. In this case, TLC revealed the total consumption of the starting material after 10 min.

*2-Benzoyl-N-cyclohexyl-5-oxotetrahydrofuran-2-carboxamide* (**11a**). White solid. Yield: 71% by method A and 93% by method B. M. p. 105–106 °C. ^1^H NMR (400 MHz, CDCl_3_) δ (ppm): 8.03–7.99 (m, 2H), 7.59–7.55 (m, 1H), 7.46–7.40 (m, 2H), 6.49 (d, *J* = 8.2 Hz, 1H, NH), 3.80–3.71 (m, 1H), 3.34–3.27 (m, 1H), 2.64 (dd, *J* = 7.0, 1.1 Hz, 1H), 2.62 (d, *J* = 7.0 Hz, 1H), 2.49–2.41 (m, 1H), 1.90–1.08 (m, 10H). ^13^C NMR {DEPT-135} (100 MHz, CDCl_3_) δ (ppm): 191.2 (C_q_), 174.9 (C_q_), 167.1 (C_q_), 134.1 (CH_Ar_), 133.5 (C_q_), 129.7 (CH_Ar_), 128.7 (CH_Ar_), 89.1 (C_q_), 48.7 (CH), 32.8 (CH_2_), 32.7 (CH_2_), 29.1 (CH_2_), 27.9 (CH_2_), 25.4 (CH_2_), 24.9 (CH_2_), 24.8 (CH_2_). HRMS (ESI): calculated for C_18_H_22_NO_4_ [MH^+^] 316.1543, found 316.1547.

*2-Benzoyl-N-butyl-5-oxotetrahydrofuran-2-carboxamide* (**11b**). White solid. Yield: 68%. M. p. 89–90 °C. ^1^H NMR (300 MHz, CDCl_3_) δ (ppm): 8.09–7.99 (m, 2H), 7.61–7.55 (m, 1H), 7.48–7.40 (m, 2H), 6.65–6.50 (m, 1H, NH), 3.42–3.18 (m, 3H), 2.67–2.61 (m, 2H), 2.56–2.46 (m, 1H), 1.53–1.43 (m, 2H), 1.36–1.23 (m, 2H), 0.89 (t, *J* = 7.3 Hz, 3H). ^13^C NMR {DEPT-135} (75 MHz, CDCl_3_) δ (ppm): 191.3 (C_q_), 175.0 (C_q_), 167.9 (C_q_), 134.0 (CH_Ar_), 133.5 (C_q_), 129.7 (CH_Ar_), 128.6 (CH_Ar_), 89.3 (C_q_), 39.4 (CH_2_), 31.2 (CH_2_), 29.1 (CH_2_), 27.8 (CH_2_), 20.0 (CH_2_), 13.7 (CH_3_). HRMS (ESI): calculated for C_16_H_20_NO_4_ [MH^+^] 290.1387, found 290.1391.

*2-Benzoyl-N-(tert-butyl)-5-oxotetrahydrofuran-2-carboxamide* (**11c**). Pale yellow oil. Yield: 70%. ^1^H NMR (300 MHz, CDCl_3_) δ (ppm): 8.03–7.99 (m, 2H), 7.60–7.54 (m, 1H), 7.47–7.41 (m, 2H), 6.33 (s, 1H, NH), 3.32–3.23 (m, 1H), 2.65–2.60 (m, 2H), 2.50–2.39 (m, 1H), 1.32 (s, 9H). ^13^C NMR {DEPT-135} (75 MHz, CDCl_3_) δ (ppm): 191.4 (C_q_), 174.9 (C_q_), 167.1 (C_q_), 134.0 (CH_Ar_), 133.5 (C_q_), 129.7 (CH_Ar_), 128.6 (CH_Ar_), 89.1 (C_q_), 52.2 (C_q_), 29.0 (CH_2_), 28.5 (CH_3_), 27.9 (CH_2_). HRMS (ESI): calculated for C_16_H_20_NO_4_ [MH^+^] 290.1387, found 290.1393.

*N-Cyclohexyl-2-(4-fluorobenzoyl)-5-oxotetrahydrofuran-2-carboxamide* (**11d**). Pale yellow oil. Yield: 73%. ^1^H NMR (300 MHz, CDCl_3_) δ (ppm): 8.10–8.03 (m, 2H), 7.15–7.07 (m, 2H), 6.45 (d, *J* = 8.2 Hz, 1H, NH), 3.82–3.69 (m, 1H), 3.36–3.27 (m, 1H), 2.66–2.61 (m, *2*H), 2.48–2.38 (m, 1H), 1.93–1.07 (m, 10H). ^13^C NMR {DEPT-135} (75 MHz, CDCl_3_) δ (ppm): 189.6 (C_q_), 174.7 (C_q_), 167.0 (C_q_), 166.3 (C_q_, d, ^1^*J* = 257 Hz), 132.6 (CH_Ar_, d, ^3^*J* = 9.5 Hz), 129.9 (C_q_, d, ^4^*J* = 3.0 Hz), 116.0 (CH_Ar_, d, ^2^*J* = 22 Hz), 89.0 (C_q_), 48.8 (CH), 32.8 (CH_2_), 32.8 (CH_2_), 29.1 (CH_2_), 27.8 (CH_2_), 25.4 (CH_2_), 24.9 (CH_2_), 24.8 (CH_2_). ^19^F NMR (300 MHz, CDCl_3_) δ: −102.9 (tt, *J* = 8.2, 5.3 Hz). HRMS (ESI): calculated for C_18_H_21_FNO_4_ [MH^+^] 334.1449, found 334.1458.

#### 3.2.4. Hydrolysis Products

##### Carboxylic Acids

The corresponding Passerini adduct **7** (0.5 mmol) was dissolved in a tetrahydrofuran–water mixture (6 mL; 5 mL of the former and 1 mL of the latter) and cesium carbonate (1.2 equiv.) was added. The reaction mixture was heated to reflux with stirring until TLC revealed the total consumption of the starting material and the formed lactone (around 4 h). Subsequently, the unreacted salt was filtered off and the organic solvent of the filtrate removed under reduced pressure. Water (10 mL) was added to the crude and this solution washed with dichloromethane (2 × 20 mL). The organic phase was discarded and a hydrochloric acid aqueous solution (10 mL) was added. The aqueous phase was extracted with dichloromethane (2 × 20 mL) and the organic phase dried over anhydrous sodium sulfate, filtered and concentrated to dryness. The residue was purified by column chromatography, employing silica gel as a stationary phase and an increasingly polar eluent (from a 4:1 hexane-ethyl acetate mixture to pure ethyl acetate). Slow evaporation of a solution of compound **12c** in acetonitrile provided colorless single crystals, suitable for X-ray diffraction analysis.

*4-Benzoyl-5-(cyclohexylamino)-4-hydroxy-5-oxopentanoic acid* (**12a**). White solid. Yield: 80%. M. p. 137–138 °C. ^1^H NMR (300 MHz, CDCl_3_) δ (ppm): 8.26–8.21 (m, 2H), 7.60–7.53 (m, 1H), 7.47–7.40 (m, 2H), 6.71 (d, *J* = 8.1 Hz, 1H, NH), 5.30 (s_b_, 1H, OH), 3.77–3.65 (m, 1H), 2.63–2.36 (m, 4H), 1.93–1.06 (m, 10H). ^13^C NMR {DEPT-135} (75 MHz, CDCl_3_) δ (ppm): 199.6 (C_q_), 178.8 (C_q_), 168.7 (C_q_), 134.0 (C_q_), 133.9 (CH_Ar_), 130.7 (CH_Ar_), 128.5 (CH_Ar_), 83.3 (C_q_), 49.2 (CH), 33.4 (CH_2_), 32.8 (CH_2_), 32.8 (CH_2_), 28.7 (CH_2_), 25.5 (CH_2_), 24.8 (CH_2_). HRMS (ESI): calculated for C_18_H_24_NO_5_ [MH^+^] 334.1649, found 334.1654.

*4-Benzoyl-5-(butylamino)-4-hydroxy-5-oxopentanoic acid* (**12b**). White solid. Yield: 99%. M. p. 135–136 °C. ^1^H NMR (300 MHz, DMSO-*d*_6_) δ (ppm): 12.15 (s_b_, 1H, OH), 8.36 (t, *J* = 5.8 Hz, 1H, NH), 8.05–8.00 (m, 2H), 7.61–7.54 (m, 1H), 7.48–7.40 (m, 2H), 6.75 (s_b_, 1H, OH), 3.18–2.99 (m, 2H), 2.40–2.02 (m, 4H), 1.45–1.33 (m, 2H), 1.29–1.17 (m, 2H), 0.83 (t, *J* = 7.3 Hz, 3H). ^13^C NMR {DEPT-135} (75 MHz, DMSO-*d*_6_) δ (ppm): 196.0 (C_q_), 174.4 (C_q_), 170.4 (C_q_), 134.8 (C_q_), 132.9 (CH_Ar_), 129.3 (CH_Ar_), 128.2 (CH_Ar_), 81.3 (C_q_), 38.2 (CH_2_), 31.3 (CH_2_), 31.0 (CH_2_), 28.2 (CH_2_), 19.6 (CH_2_), 13.7 (CH_3_). HRMS (ESI): calculated for C_16_H_22_NO_5_ [MH^+^] 308.1492, found 308.1480.

*4-Benzoyl-5-(tert-butylamino)-4-hydroxy-5-oxopentanoic acid* (**12c**). White solid. Yield: 99%. M. p. 125–127 °C. ^1^H NMR (300 MHz, CDCl_3_) δ (ppm): 8.21–8.15 (m, 2H), 7.60–7.53 (m, 1H), 7.46–7.41 (m, 2H), 6.60 (s, 1H, NH), 5.24 (s_b_, 1H, OH), 2.55–2.35 (m, 4H), 1.32 (s, 9H). ^13^C NMR {DEPT-135} (75 MHz, CDCl_3_) δ (ppm): 200.3 (C_q_), 168.7 (C_q_), 134.4 (C_q_), 133.8 (CH_Ar_), 130.5 (CH_Ar_), 128.5 (CH_Ar_), 83.4 (C_q_), 52.0 (C_q_), 33.4 (CH_2_), 28.5 (CH_3_). HRMS (ESI): calculated for C_16_H_22_NO_5_ [MH^+^] 308.1492, found 308.1494.

*5-(Cyclohexylamino)-4-(4-fluorobenzoyl)-4-hydroxy-5-oxopentanoic acid* (**12d**). White solid. Yield: 98%. M. p. 136–137 °C. ^1^H NMR (300 MHz, CD_3_CN) δ (ppm): 8.16–8.11 (m, 2H), 7.20–7.14 (m, 3H), 3.62 (s_b_, 1H), 2.40–2.25 (m, 4H), 1.85–1.07 (m, 10H). ^13^C NMR {DEPT-135} (75 MHz, CD_3_CN) δ (ppm): 196.5 (C_q_), 175.5 (C_q_), 170.3 (C_q_), 166.5 (C_q_, d, ^1^*J* = 253 Hz), 133.5 (CH_Ar_, d, ^3^*J* = 9.4 Hz), 132.4 (C_q_, d, ^4^*J* = 3.0 Hz), 116.2 (CH_Ar_, d, ^2^*J* = 21 Hz), 83.2 (C_q_), 49.6 (CH), 33.2 (CH_2_), 33.0 (CH_2_), 32.3 (CH_2_), 28.8 (CH_2_), 26.2 (CH_2_), 25.7 (CH_2_), 25.7 (CH_2_). ^19^F NMR (300 MHz, CD_3_CN) δ: −107.1 (tt, *J* = 8.9, 5.4 Hz). HRMS (ESI): calculated for C_18_H_23_FNO_5_ [MH^+^] 352.1555, found 352.1559.

##### Methyl Esters

The corresponding lactone **11** (1 mmol) was dissolved in methanol (10 mL) and a solution of hydrochloric acid in methanol (0.5 M; 4 equiv.) was added. The reaction mixture was heated to reflux with stirring until TLC revealed the total consumption of the starting material (2–3 h). Subsequently, the solvent was removed under reduced pressure and the crude was dissolved in dichloromethane (20 mL). This solution was washed with a sodium carbonate aqueous solution (2 × 20 mL) and the organic phase dried over anhydrous sodium sulfate, filtered and concentrated to dryness. The residue was purified by column chromatography, employing silica gel as a stationary phase and a hexane-ethyl acetate mixture as an eluent. In the case of compound **13c**, addition of hexane led to the formation of a precipitate, which was isolated by vacuum filtration and dried in vacuo.

*Methyl 4-benzoyl-5-(cyclohexylamino)-4-hydroxy-5-oxopentanoate* (**13a**). Yellow oil. Yield: 91%. ^1^H NMR (300 MHz, CDCl_3_) δ (ppm): 8.24–8.19 (m, 2H), 7.57–7.51 (m, 1H), 7.45–7.38 (m, 2H), 6.75 (d, *J* = 8.0 Hz, 1H, NH), 5.34 (s, 1H, OH), 3.78–3.62 (m, 1H), 3.63 (s, 3H), 2.60–2.30 (m, 4H), 1.96–1.04 (m, 10H). ^13^C NMR {DEPT-135} (75 MHz, CDCl_3_) δ (ppm): 199.5 (C_q_), 173.9 (C_q_), 168.8 (C_q_), 134.1 (C_q_), 133.7 (CH_Ar_), 130.6 (CH_Ar_), 128.4 (CH_Ar_), 83.3 (C_q_), 51.9 (CH_3_), 49.1 (CH), 33.6 (CH_2_), 32.8 (CH_2_), 32.7 (CH_2_), 28.7 (CH_2_), 25.4 (CH_2_), 24.8 (CH_2_). HRMS (ESI): calculated for C_19_H_26_NO_5_ [MH^+^] 348.1805, found 348.1816.

*Methyl 4-benzoyl-5-(butylamino)-4-hydroxy-5-oxopentanoate* (**13b**). Pale yellow oil. Yield: 93%. ^1^H NMR (300 MHz, CDCl_3_) δ (ppm): 8.26–8.20 (m, 2H), 7.58–7.52 (m, 1H), 7.46–7.38 (m, 2H), 6.88 (t, *J* = 5.5 Hz, 1H, NH), 5.34 (s, 1H, OH), 3.63 (s, 3H), 3.28–3.20 (m, 2H), 2.65–2.29 (m, 4H), 1.50–1.40 (m, 2H), 1.33–1.21 (m, 2H), 0.86 (t, *J* = 7.3 Hz, 3H). ^13^C NMR {DEPT-135} (75 MHz, CDCl_3_) δ (ppm): 199.5 (C_q_), 173.8 (C_q_), 169.7 (C_q_), 134.1 (C_q_), 133.8 (CH_Ar_), 130.7 (CH_Ar_), 128.4 (CH_Ar_), 83.5 (C_q_), 51.9 (CH_3_), 39.9 (CH_2_), 33.7 (CH_2_), 31.4 (CH_2_), 28.7 (CH_2_), 20.0 (CH_2_), 13.7 (CH_3_). HRMS (ESI): calculated for C_17_H_24_NO_5_ [MH^+^] 322.1649, found 322.1652.

*Methyl 4-benzoyl-5-(tert-butylamino)-4-hydroxy-5-oxopentanoate* (**13c**). White solid. Yield: 90%. M. p. 135–136 °C. ^1^H NMR (300 MHz, CDCl_3_) δ (ppm): 8.18–8.11 (m, 2H), 7.57–7.48 (m, 1H), 7.45–7.35 (m, 2H), 6.63 (s, 1H, NH), 5.29 (s, 1H, OH), 3.61 (s, 3H), 2.52–2.30 (m, 4H), 1.29 (s, 9H). ^13^C NMR {DEPT-135} (75 MHz, CDCl_3_) δ (ppm): 200.1 (C_q_), 173.8 (C_q_), 168.7 (C_q_), 134.4 (C_q_), 133.6 (CH_Ar_), 130.4 (CH_Ar_), 128.3 (CH_Ar_), 83.3 (C_q_), 51.8 (CH_3_), 33.5 (CH_2_), 28.6 (CH_2_), 28.5 (CH_3_). HRMS (ESI): calculated for C_17_H_24_NO_5_ [MH^+^] 322.1649, found 322.1654.

*Methyl 5-(cyclohexylamino)-4-(4-fluorobenzoyl)-4-hydroxy-5-oxopentanoate* (**13d**). Yellow oil. Yield: 62%. ^1^H NMR (300 MHz, CDCl_3_) δ (ppm): 8.33–8.25 (m, 2H), 7.10–7.01 (m, 2H), 6.80 (d, *J* = 8.1 Hz, 1H, NH), 5.38 (s, 1H, OH), 3.73–3.59 (m, 1H), 3.60 (s, 3H), 2.54–2.28 (m, 4H), 1.91–1.03 (m, 10H). ^13^C NMR {DEPT-135} (75 MHz, CDCl_3_) δ (ppm): 197.5 (C_q_), 173.8 (C_q_), 168.7 (C_q_), 166.0 (C_q_, d, ^1^*J* = 257 Hz), 133.7 (CH_Ar_, d, ^3^*J* = 9.4 Hz), 130.3 (C_q_, d, ^4^*J* = 3.0 Hz), 115.5 (CH_Ar_, d, ^2^*J* = 22 Hz), 83.2 (C_q_), 51.9 (CH_3_), 49.0 (CH), 33.5 (CH_2_), 32.7 (CH_2_), 32.7 (CH_2_), 28.6 (CH_2_), 25.4 (CH_2_), 24.7 (CH_2_). ^19^F NMR (300 MHz, CDCl_3_) δ: −103.5 (tt, *J* = 8.3, 5.6 Hz). HRMS (ESI): calculated for C_19_H_25_FNO_5_ [MH^+^] 366.1711, found 366.1714.

## 4. Conclusions

Isocyanide-based multicomponent reactions, such as Ugi and Passerini reactions, constitute a powerful tool for the synthesis of highly functionalized systems. Obtention of β-ketoamides has allowed to study keto-enol tautomerism and to determine the influence of C2 substitution on the tautomeric equilibrium. Furthermore, we proved the versatility of Passerini adducts, given that depending on the conditions of the reactions performed on them different products, and not only γ-lactones, can be obtained.

## Figures and Tables

**Table 1:** Gibbs energies of tautomers and equilibrium constants.

Entry	Adduct (X)	Medium	ΔG_keto_ (kcal mol^−1^) ^a^	ΔG_enol_ (kcal mol^−1^) ^a^	K_eq_^b^
1	**5** (O)	Gas Chloroform	0.0 0.0	4.2 3.9	8.0 × 10^−4^ 1.4 × 10^−3^
2	**6**_NH_ (NH)	Gas Chloroform	0.0 0.0	2.0 2.5	3.4 × 10^−2^ 1.5 × 10^−2^
3	**6**_NMe_ (NCH_3_)	Gas Chloroform	6.7 5.6	0.0 0.0	8.1 × 10^4^ 1.2 × 10^4^
4	**6** (NCH_2_C_6_H_5_)	Gas Chloroform	7.2 6.3	0.0 0.0	1.9 × 10^5^ 4.0 × 10^4^
5	**6**_N*t*Bu_ (NC(CH_3_)_3_)	Gas Chloroform	15.1 13.4	0.0 0.0	1.2 × 10^11^ 7.0 × 10^9^

^a^ Gibbs energy referred to the most stable tautomer. ^b^ Equilibrium constant calculated using the Arrhenius equation.

**Table 2:** Synthesis of γ-lactones **11** and 2-hydroxyglutaric acid derivatives **12** from Passerini adducts **7**.

Entry	1 (Ar)	3 (R)	7 (%) ^a^	11 (%) ^a^	12 (%) ^a^
1	**1a** (C_6_H_5_)	**3a** (*c*C_6_H_11_)	**7a** (93)	**11a** (71)	**12a** (80)
2	**1a** (C_6_H_5_)	**3b** (*n*C_4_H_9_)	**7b** (84)	**11b** (68)	**12b** (99)
3	**1a** (C_6_H_5_)	**3c** (*t*C_4_H_9_)	**7c** (87)	**11c** (70)	**12c** (99)
4	**1b** (4-FC_6_H_4_)	**3a** (*c*C_6_H_11_)	**7d** (86)	**11d** (73)	**12d** (98)

^a^ Isolated yield.

**Table 3:** Synthesis of 2-hydroxyglutarate derivatives **13** from γ-lactones **11**.

Entry	11 (Ar, R)	13 (%) ^a^
1	**11a** (C_6_H_5_, *c*C_6_H_11_)	**13a** (91)
2	**11b** (C_6_H_5_, *n*C_4_H_9_)	**13b** (93)
3	**11c** (C_6_H_5_, *t*C_4_H_9_)	**13c** (90)
4	**11d** (4-FC_6_H_4_, *c*C_6_H_11_)	**13d** (62)

**^a^** Isolated yield.

## Data Availability

The data presented in this study is available in this article.

## Supplementary Materials

The following are available online at: ^1^H, ^19^F, ^13^C, DEPT-135 NMR spectra and high-resolution mass spectra of the compounds, and X-ray crystallographic files in CIF format for compounds **5** (CCDC 2052029), **6** (CCDC 2052030), **9** (CCDC 2052031), **11b** (CCDC 2052027) and **12c** (CCDC 2052028).
