# Supplementary material for: Keto-Enol Tautomerism in Passerini and Ugi Adducts
Source: Molecules. 2021 Feb 9;26(4):919. doi: 10.3390/molecules26040919 (PMC7916235; doi:10.3390/molecules26040919)
Supplement: Supplementary file 1 [file molecules-26-00919-s001.zip › Supporting Information.pdf]

Supporting Information

Keto-Enol Tautomerism in Passerini and Ugi Adducts

Pablo Pertejo, Andrea Sancho-Medina, Tomás Hermosilla, Beatriz González-Saiz, Javier Gómez-Ayuso, Roberto Quesada, Daniel Moreno, Israel Carreira-Barral* and María García-Valverde*

Contents

NMR and high-resolution mass spectra of the compounds
X-ray diffraction studies

NMR and high-resolution mass spectra of the compounds

<u>1-(Cyclohexylamino)-1,3-dioxo-3-phenylpropan-2-yl propionate (5)</u>

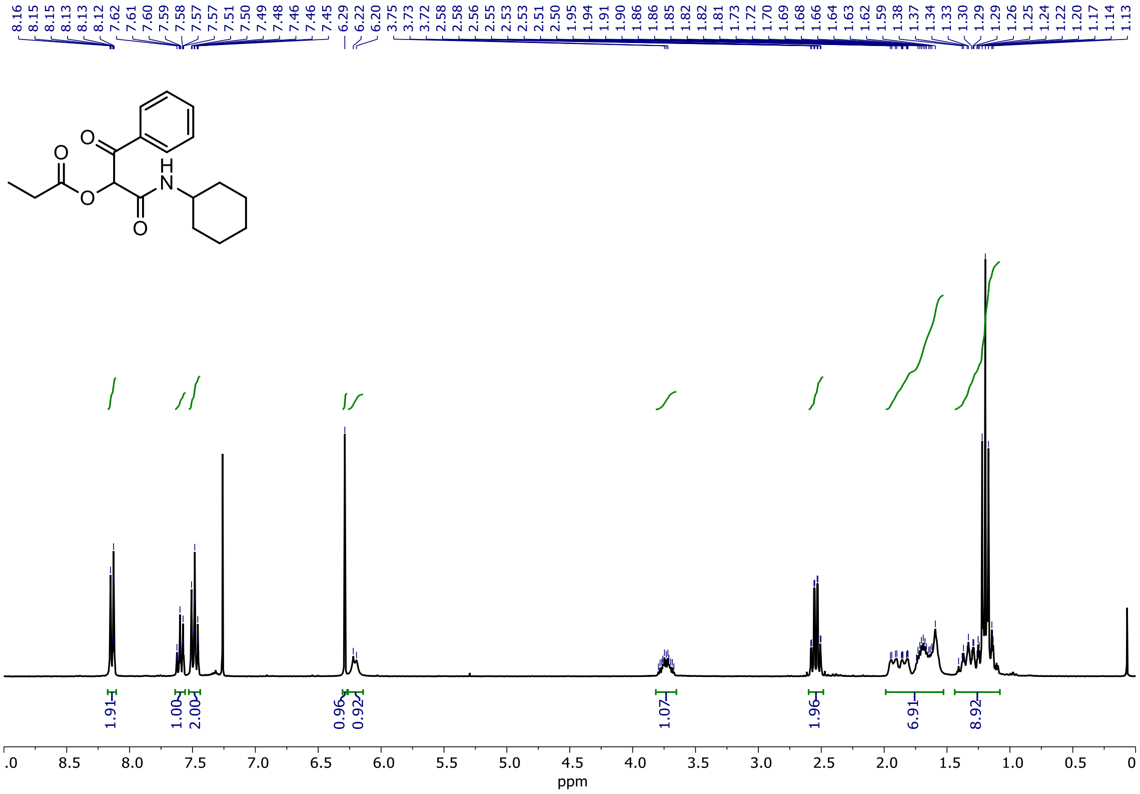

Figure 1. ^{1}H NMR spectrum of **5** (300 MHz, $CDCl_3$).

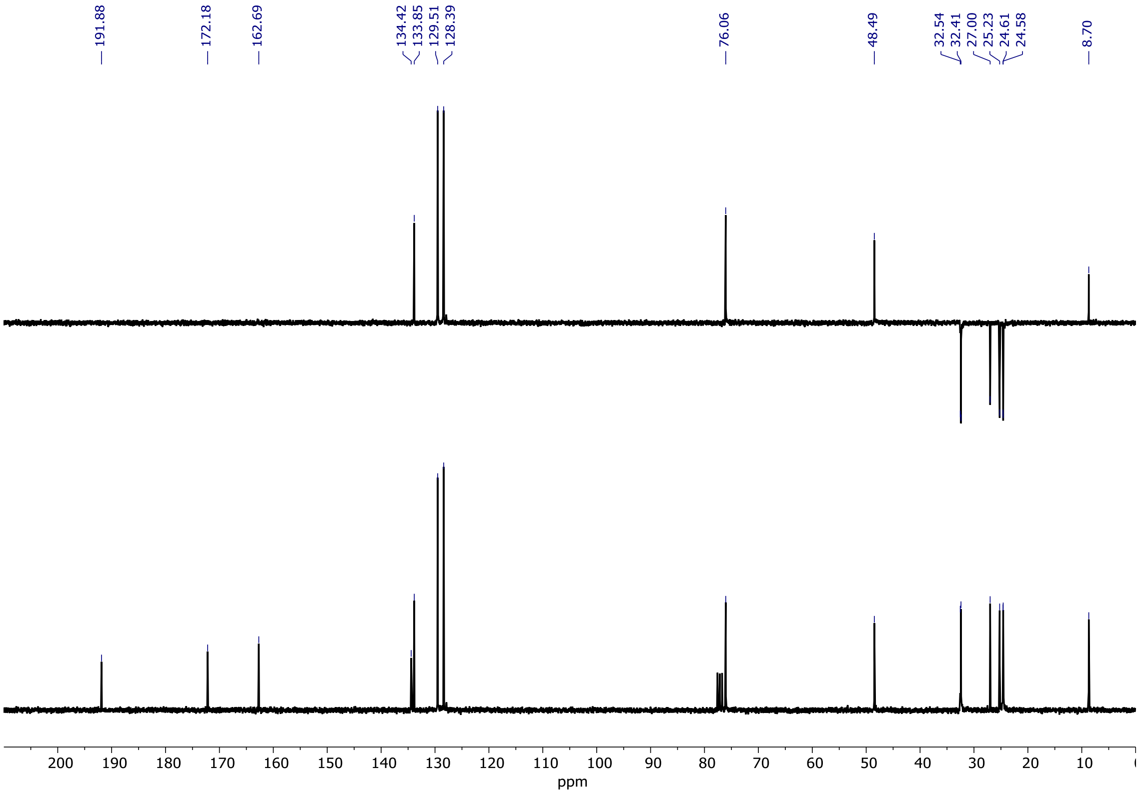

Figure 2. ^{13}C and DEPT-135 NMR spectra of **5** (75 MHz, $CDCl_3$).

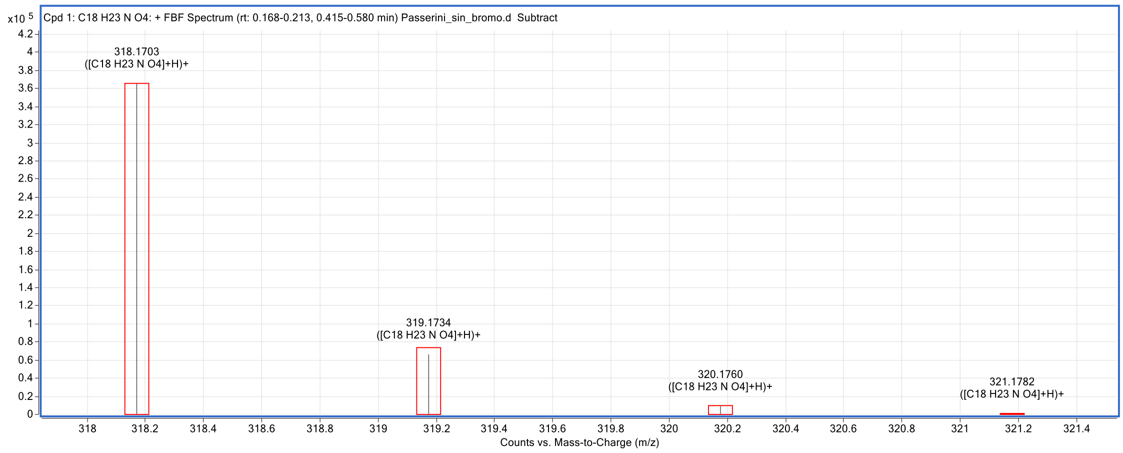

Figure 3. High-resolution mass spectrum of **5**.

(*E*)-2-(*N*-Benzylpropionamido)-*N*-cyclohexyl-3-hydroxy-3-phenylacrylamide (6)

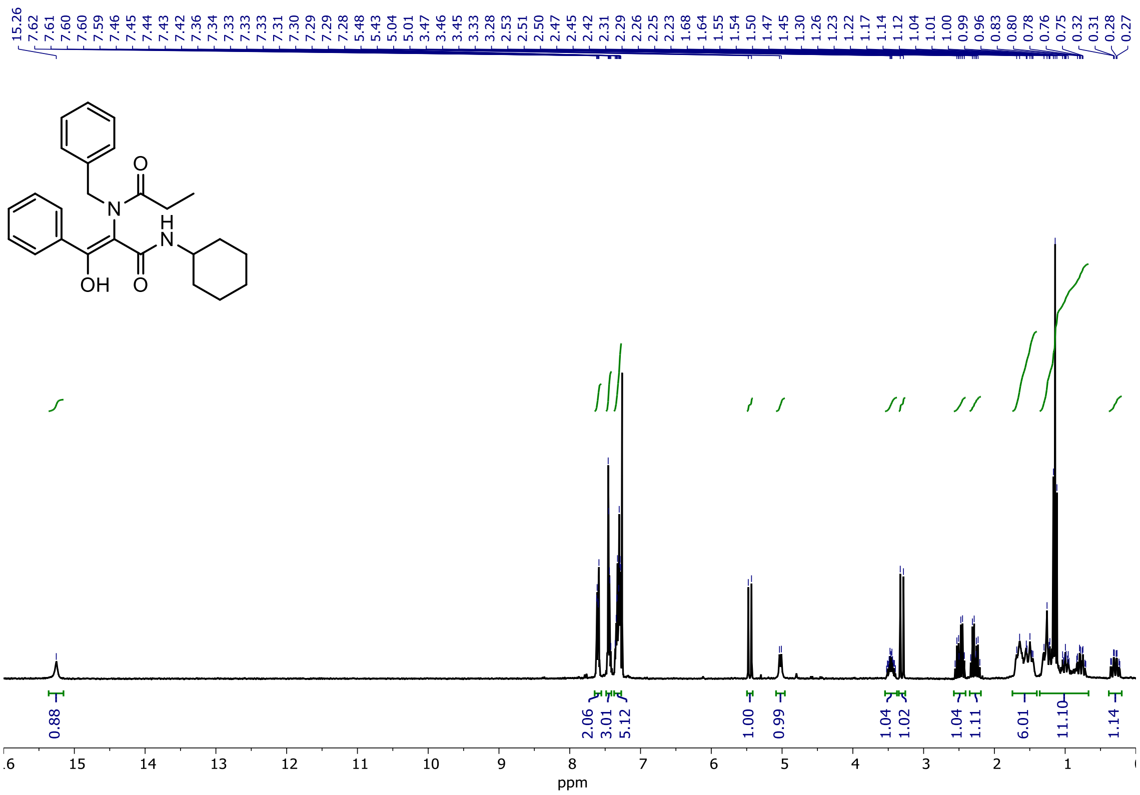

Figure 4. ^{1}H NMR spectrum of **6** (300 MHz, $CDCl_3$).

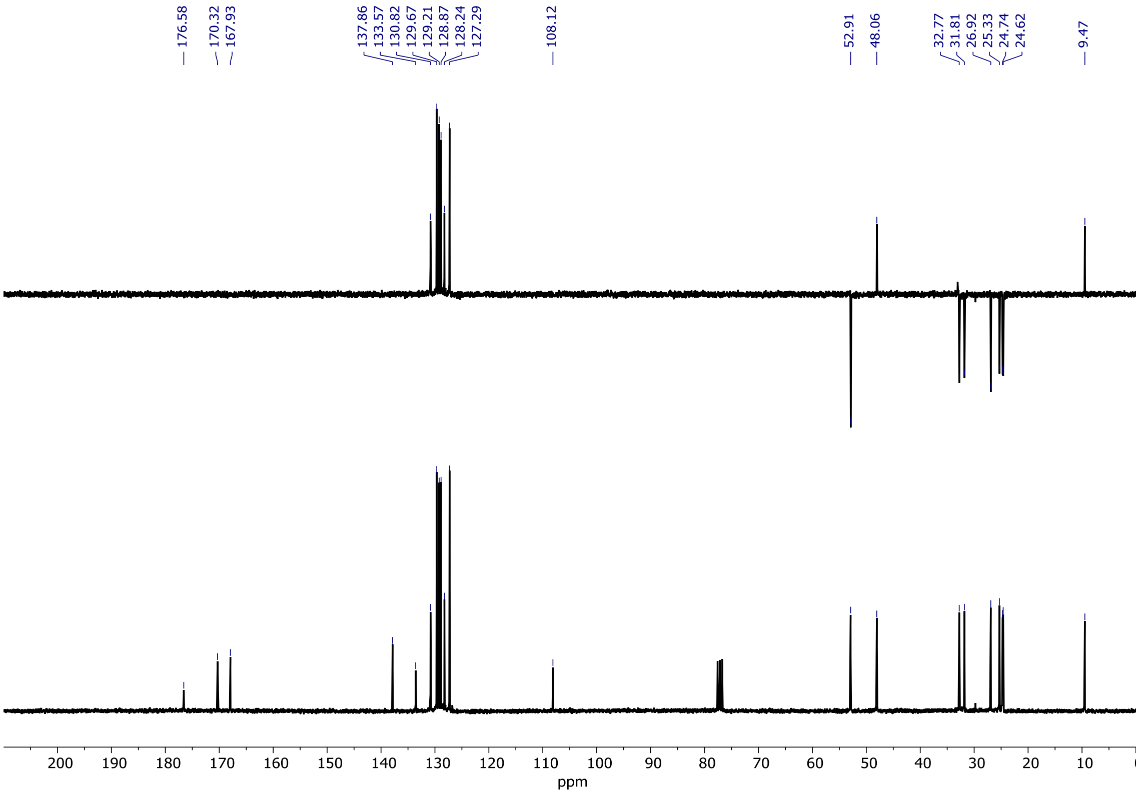

Figure 5. ^{13}C and DEPT-135 NMR spectra of **6** (75 MHz, $CDCl_3$).

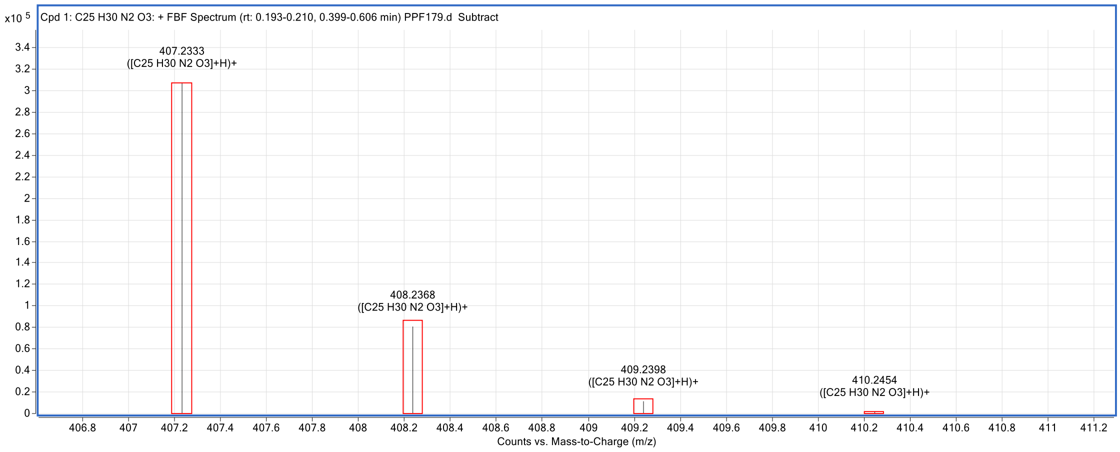

Figure 6. High-resolution mass spectrum of **6**.

1-(Cyclohexylamino)-1,3-dioxo-3-phenylpropan-2-yl 3-bromopropanoate (7a)

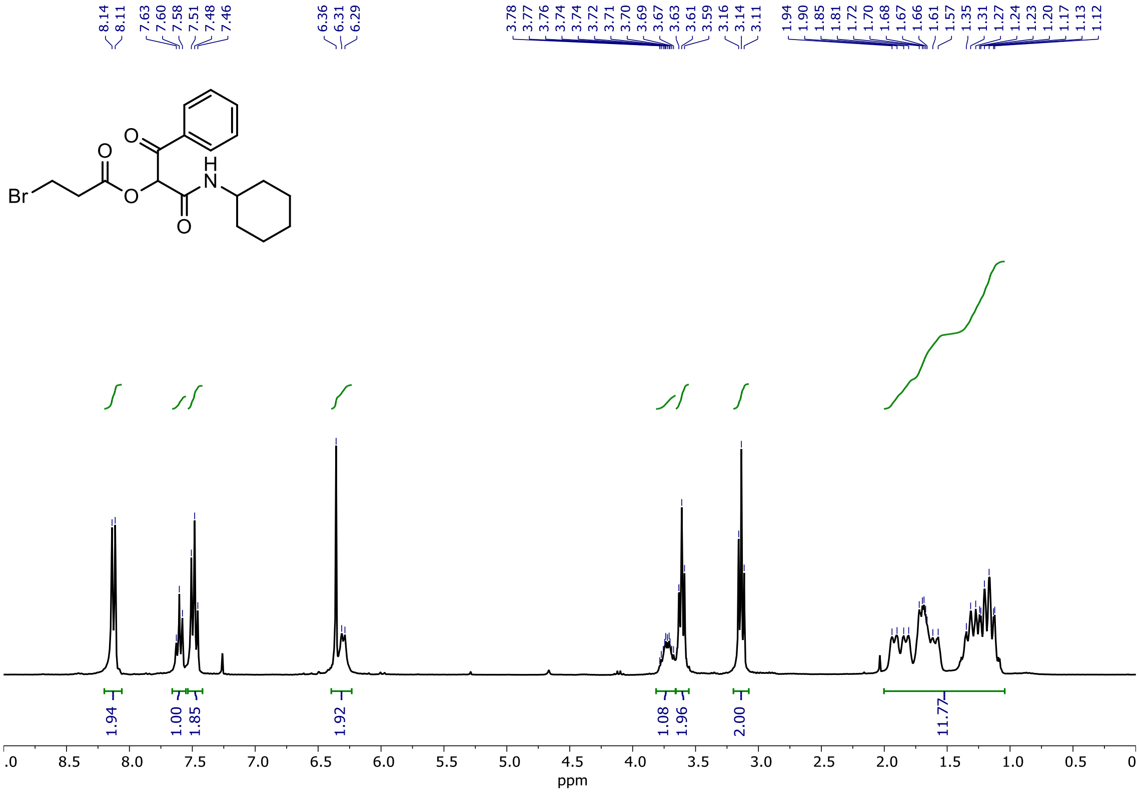

Figure 7. ^{1}H NMR spectrum of **7a** (300 MHz, $CDCl_3$).

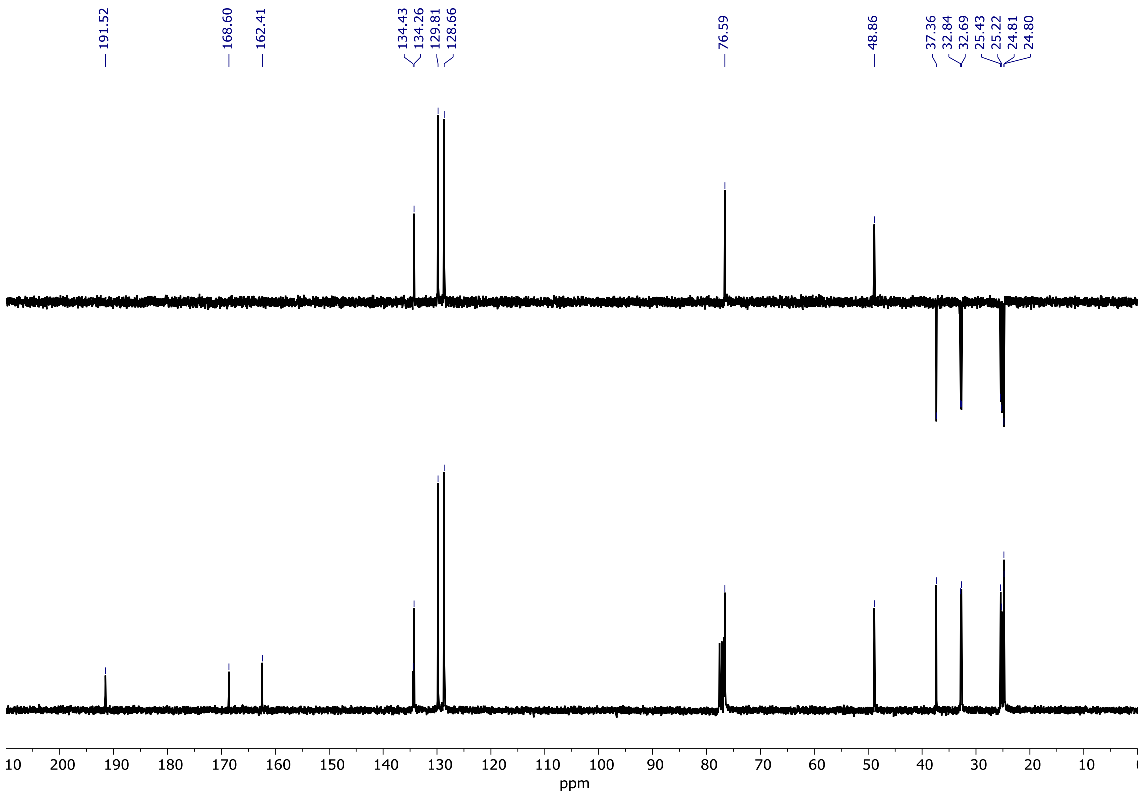

Figure 8. ^{13}C and DEPT-135 NMR spectra of **7a** (75 MHz, $CDCl_3$).

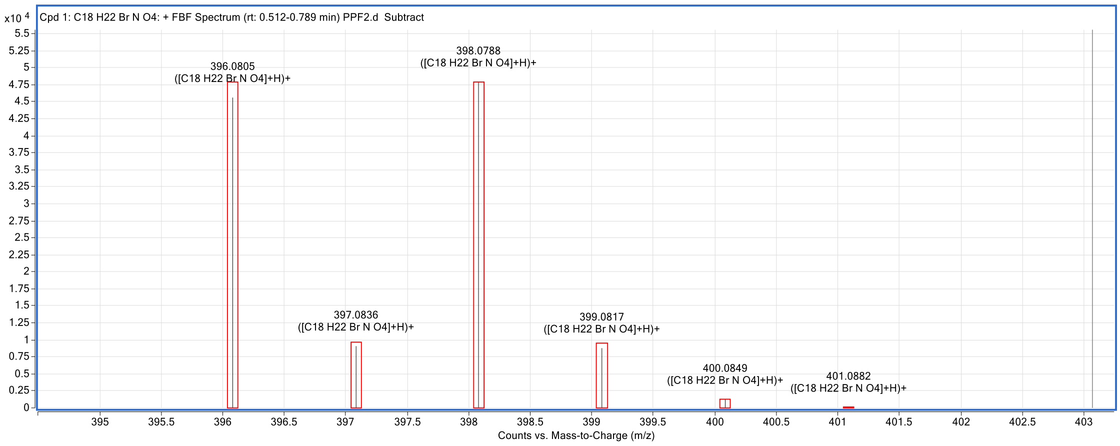

Figure 9. High-resolution mass spectrum of **7a**.

1-(Butylamino)-1,3-dioxo-3-phenylpropan-2-yl 3-bromopropanoate (7b)

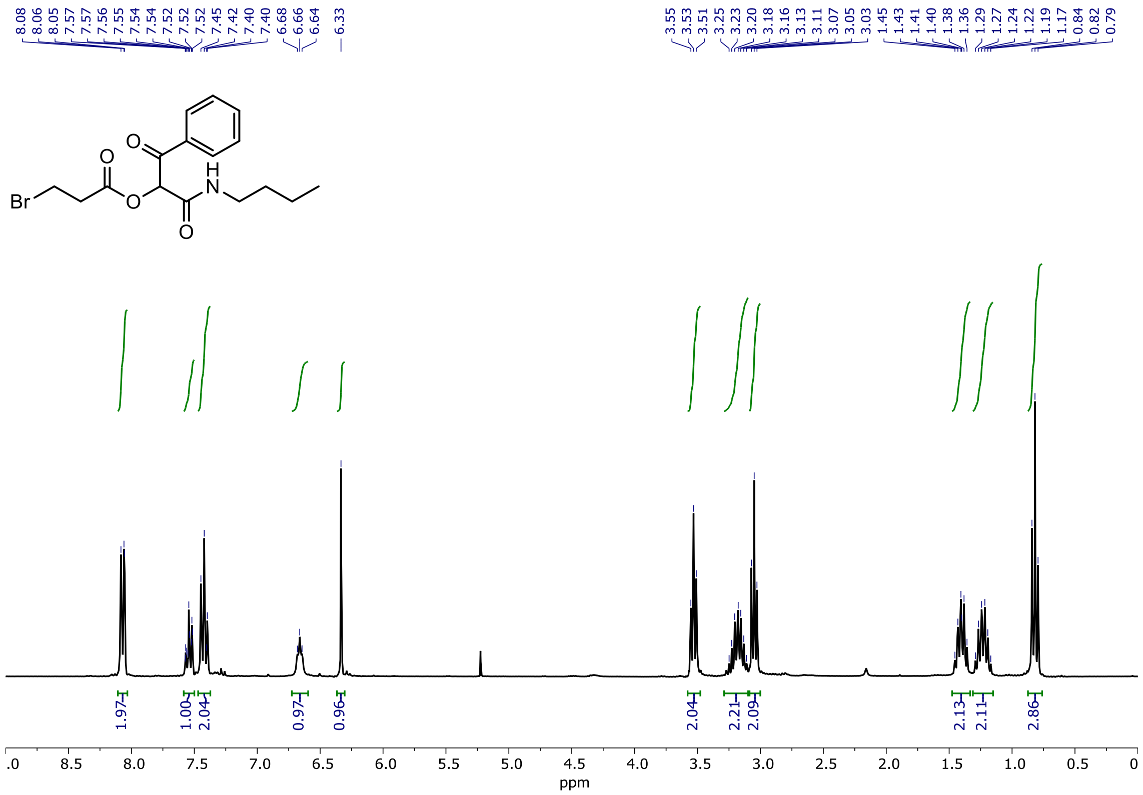

Figure 10. ^{1}H NMR spectrum of **7b** (300 MHz, $CDCl_3$).

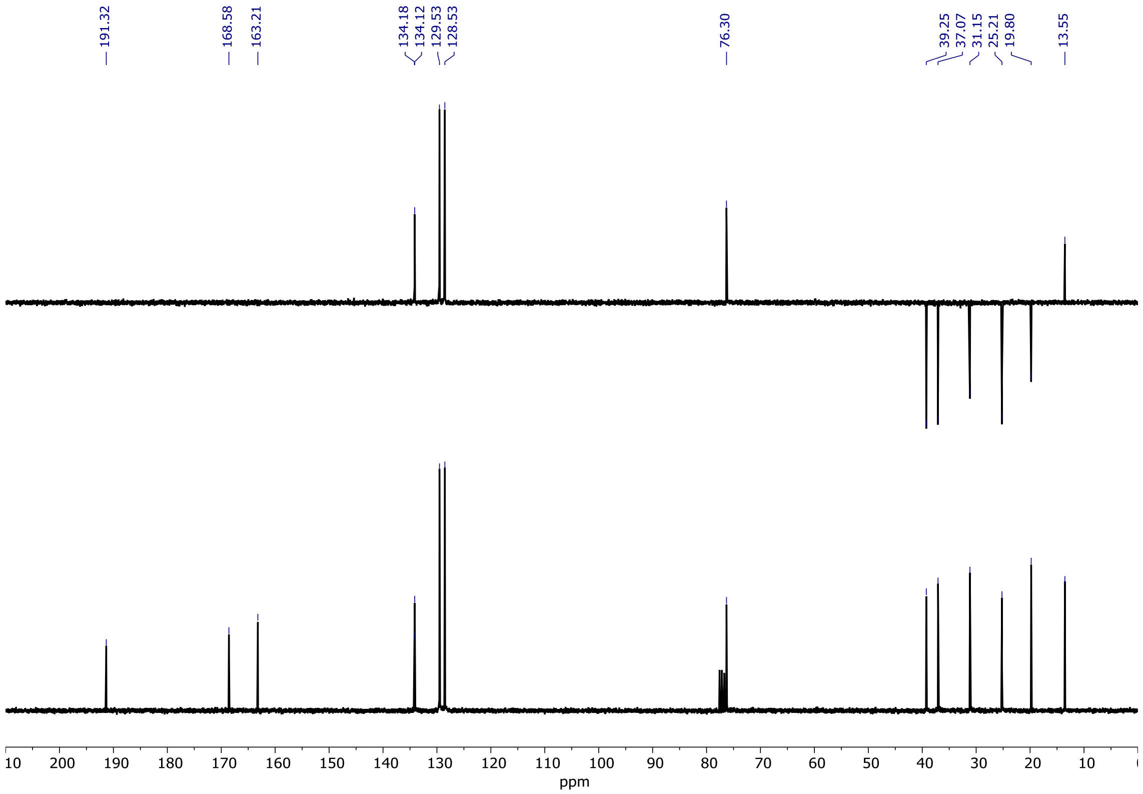

Figure 11. ^{13}C and DEPT-135 NMR spectra of **7b** (75 MHz, $CDCl_3$).

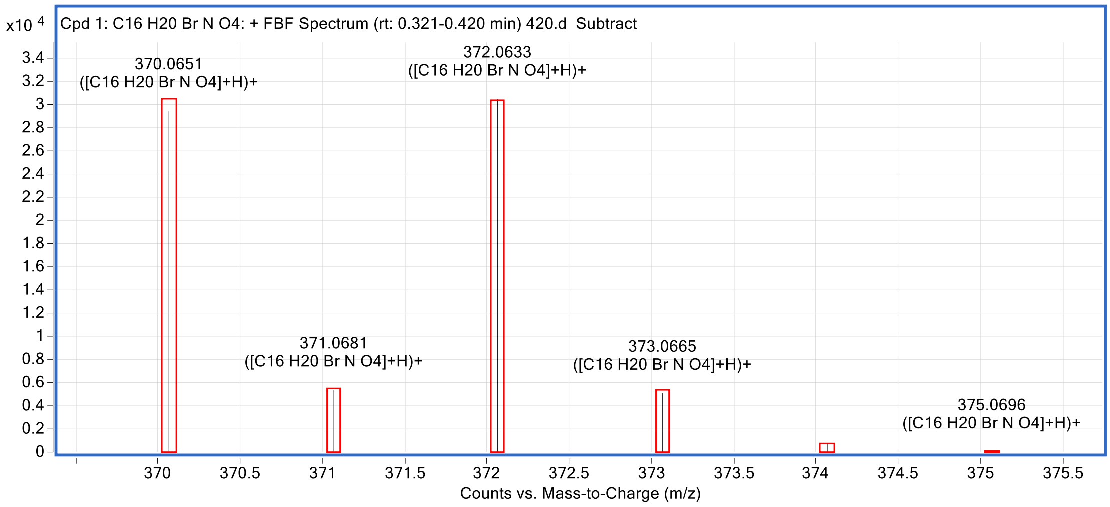

Figure 12. High-resolution mass spectrum of **7b**.

<u>1-(*tert*-Butylamino)-1,3-dioxo-3-phenylpropan-2-yl 3-bromopropanoate (7c)</u>

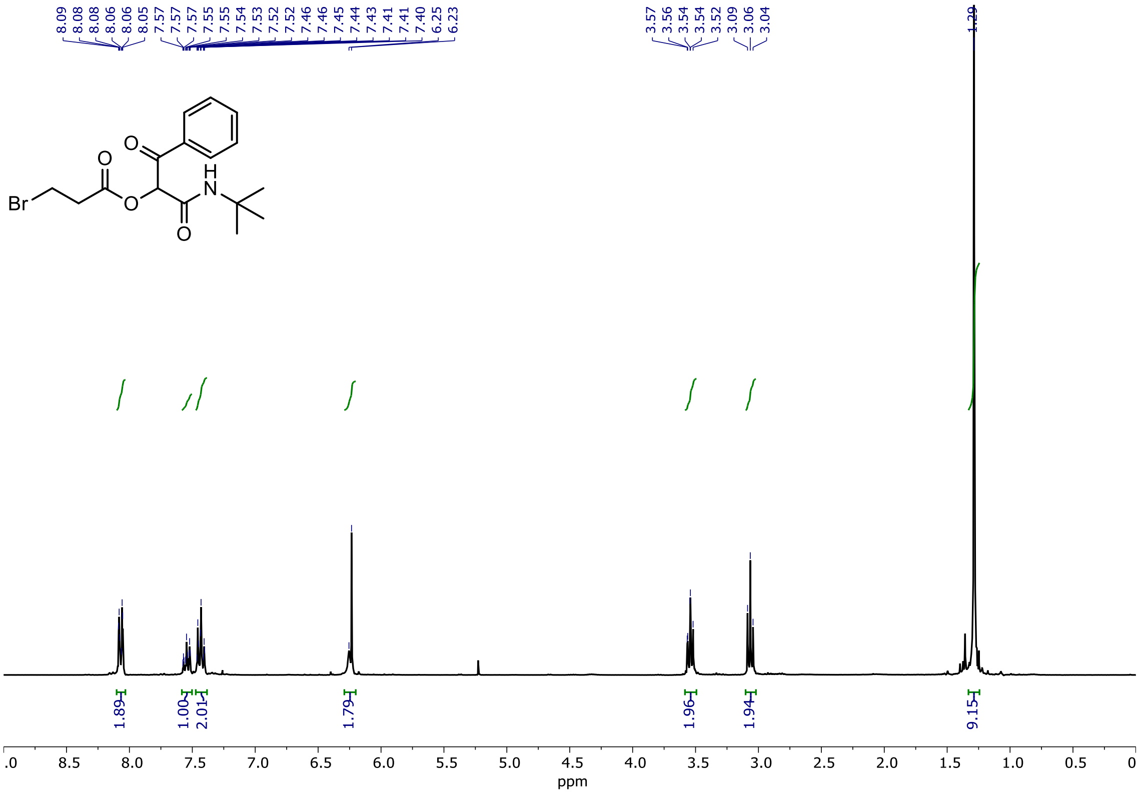

Figure 13. ¹H NMR spectrum of **7c** (300 MHz, $CDCl_3$).

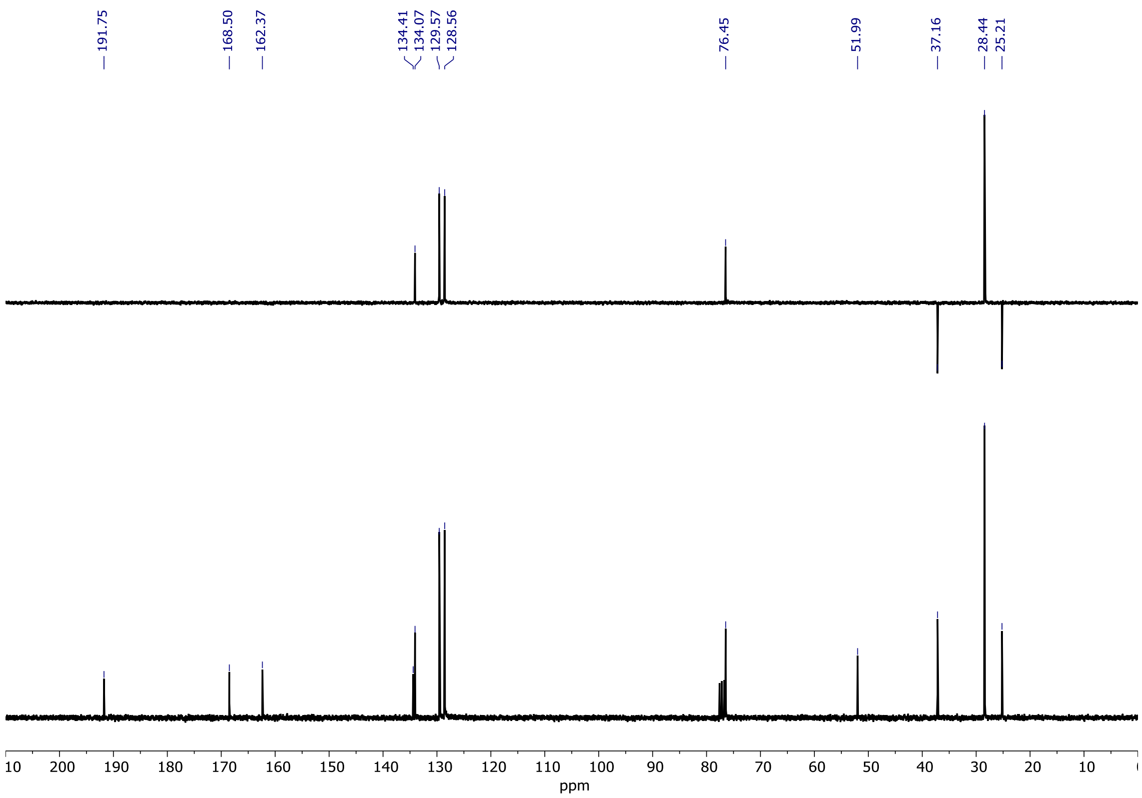

Figure 14. ^{13}C and DEPT-135 NMR spectra of **7c** (75 MHz, $CDCl_3$).

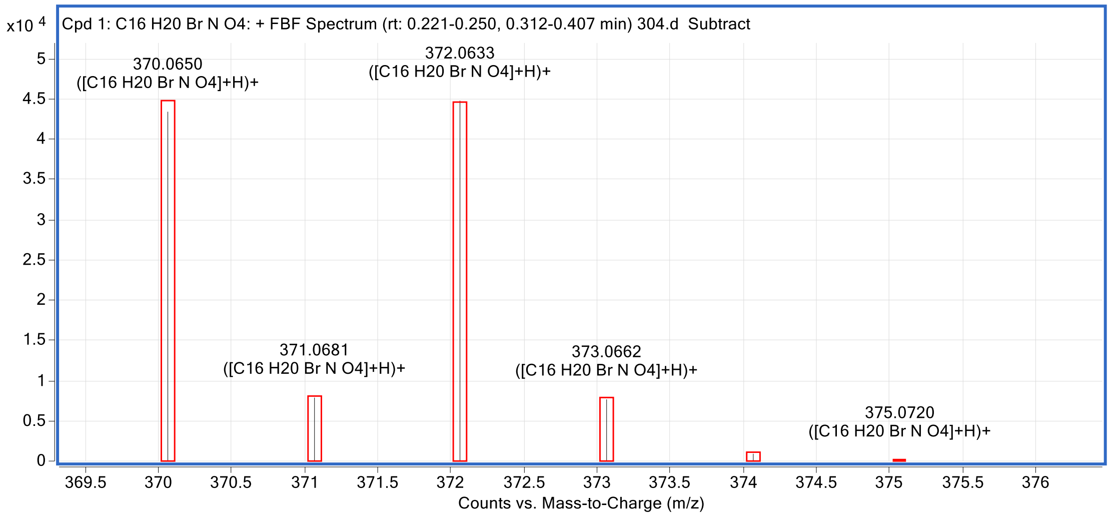

Figure 15. High-resolution mass spectrum of **7c**.

1-(Cyclohexylamino)-3-(4-fluorophenyl)-1,3-dioxopropan-2-yl 3-bromopropanoate (7d)

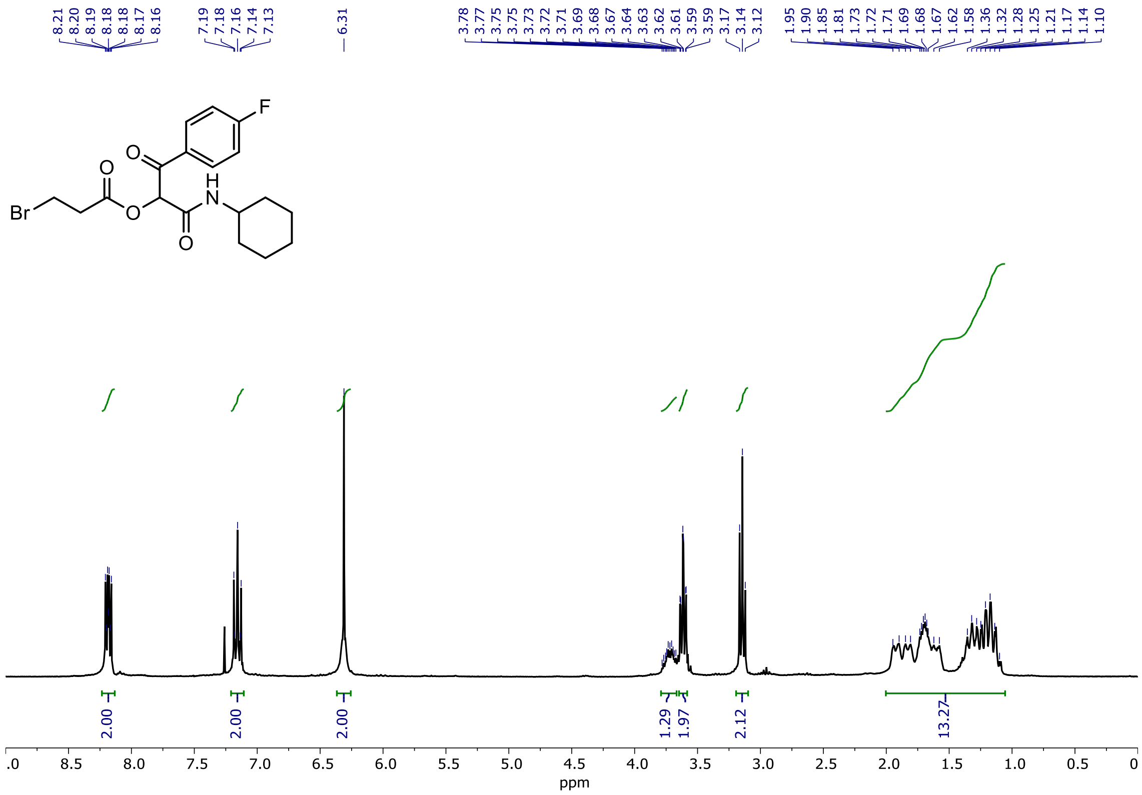

Figure 16. ^{1}H NMR spectrum of **7d** (300 MHz, $CDCl_3$).

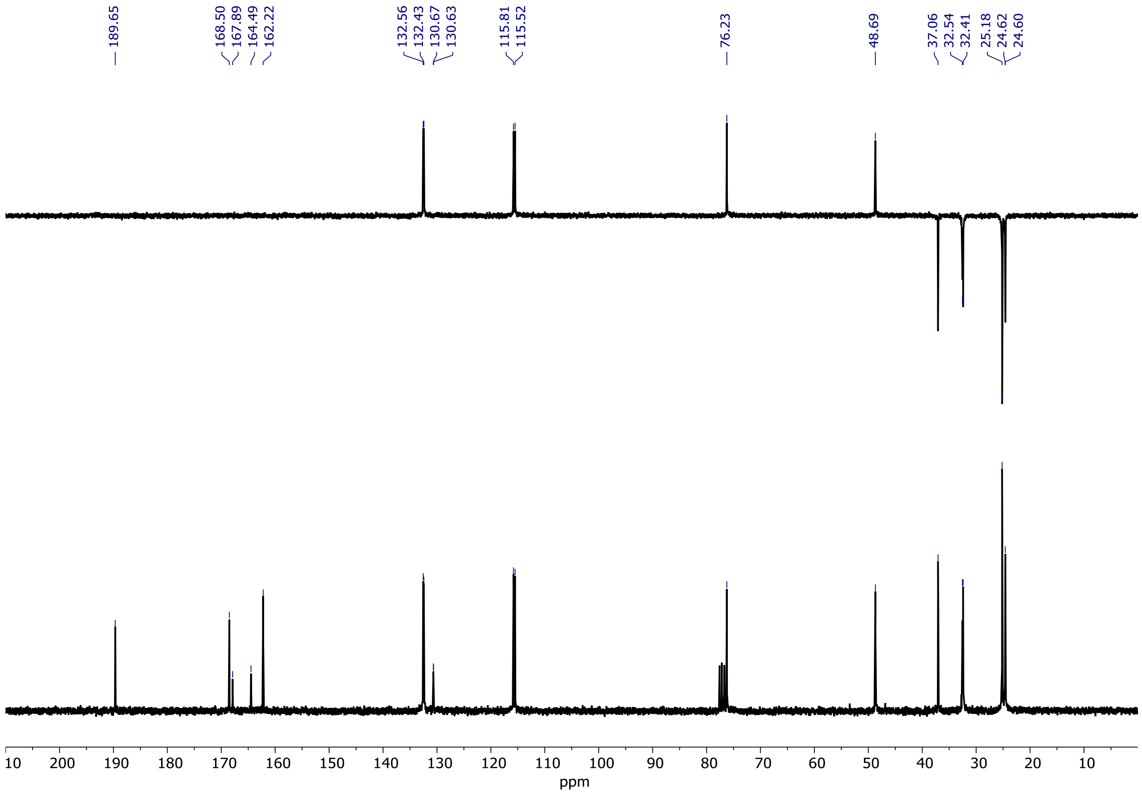

Figure 17. ^{13}C and DEPT-135 NMR spectra of **7d** (75 MHz, $CDCl_3$).

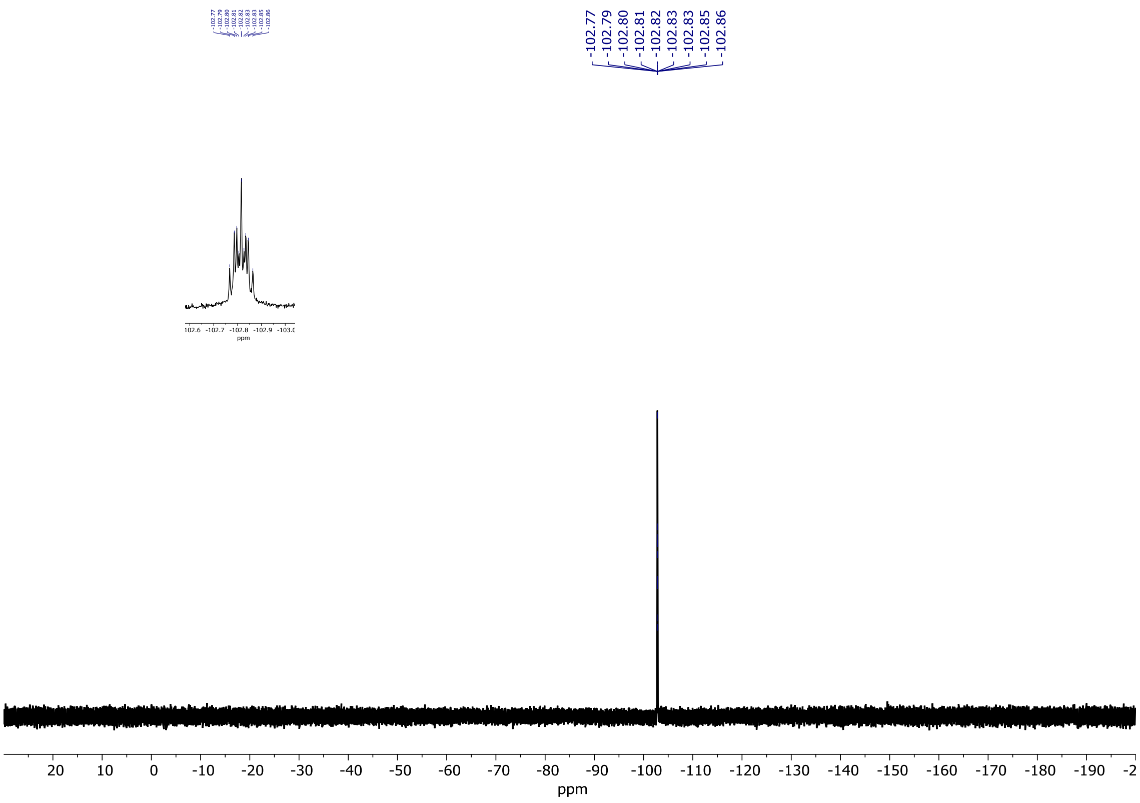

Figure 18. ^{19}F NMR spectrum of **7d** (300 MHz, $CDCl_3$).

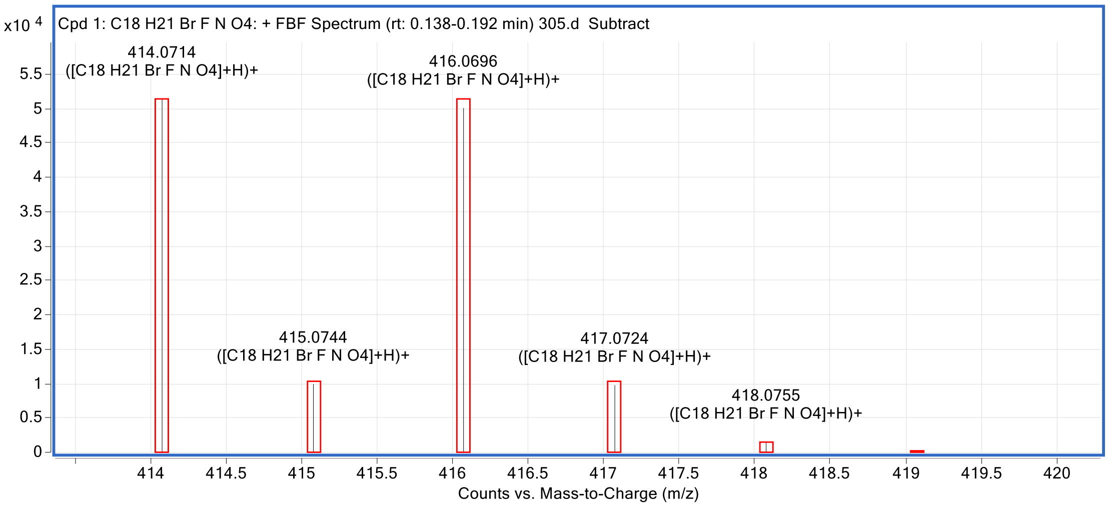

Figure 19. High-resolution mass spectrum of **7d**.

2-Benzoyl-1-benzyl-*N*-cyclohexyl-5-oxopyrrolidine-2-carboxamide (9)

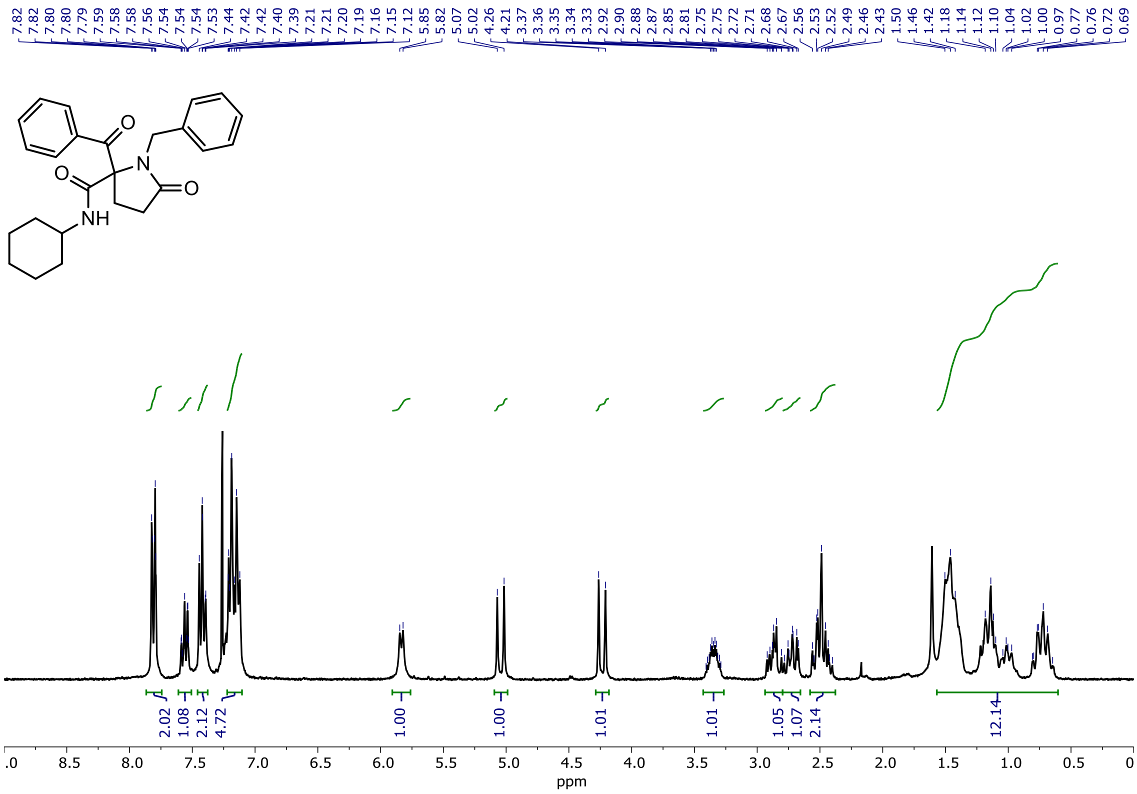

Figure 20. ^{1}H NMR spectrum of **9** (300 MHz, $CDCl_3$).

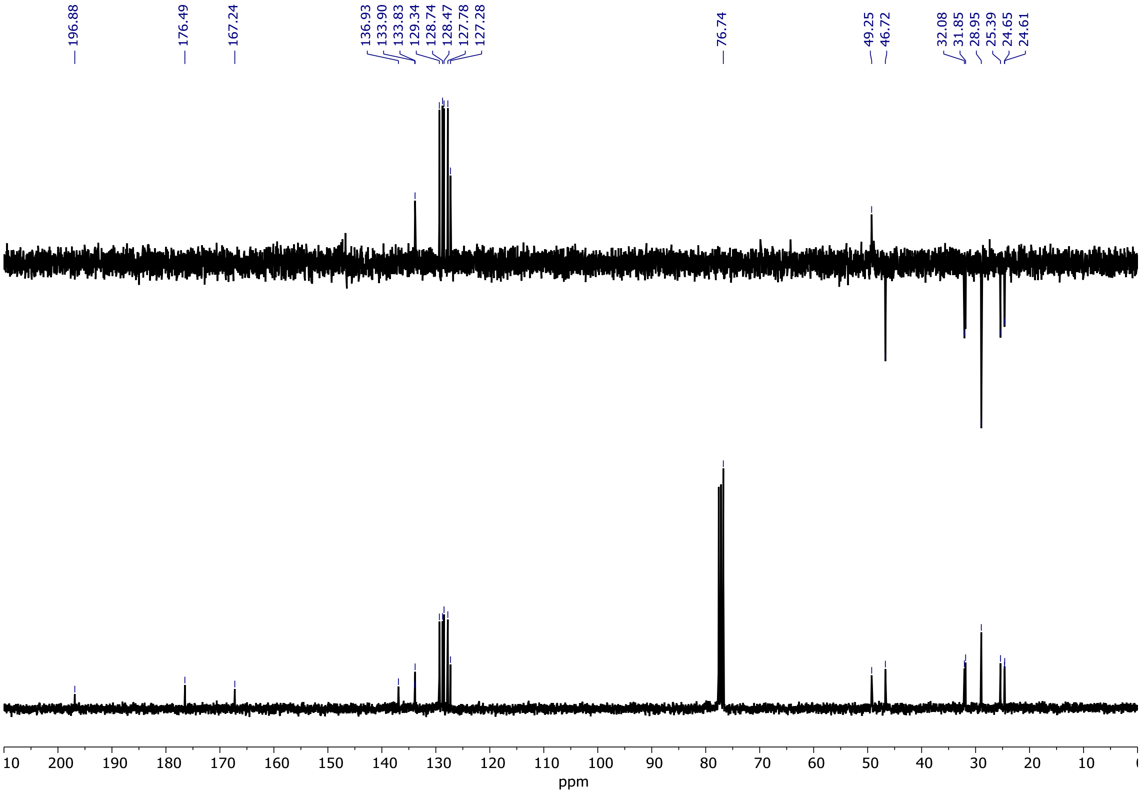

Figure 21. ^{13}C and DEPT-135 NMR spectra of **9** (75 MHz, $CDCl_3$).

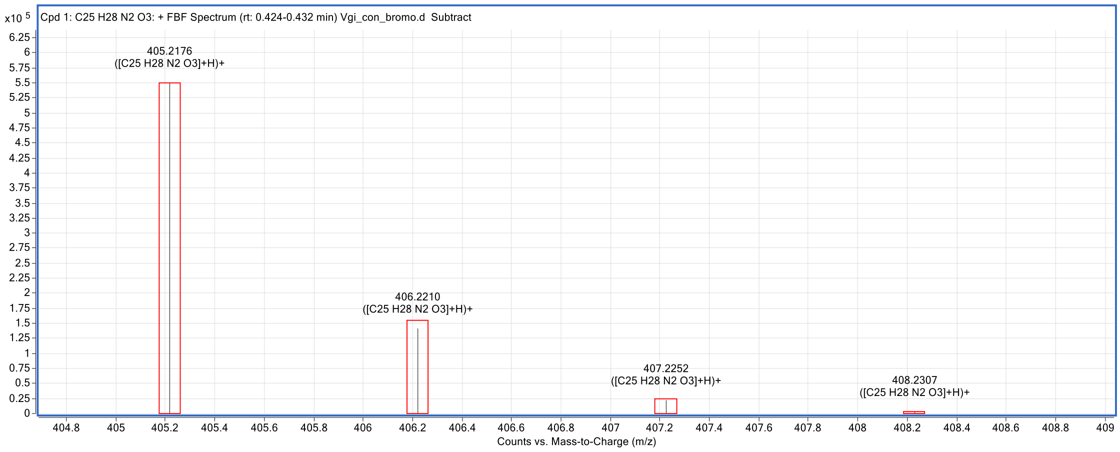

Figure 22. High-resolution mass spectrum of **9**.

1-(Cyclohexylamino)-1,3-dioxo-3-phenylpropan-2-yl acrylate (10)

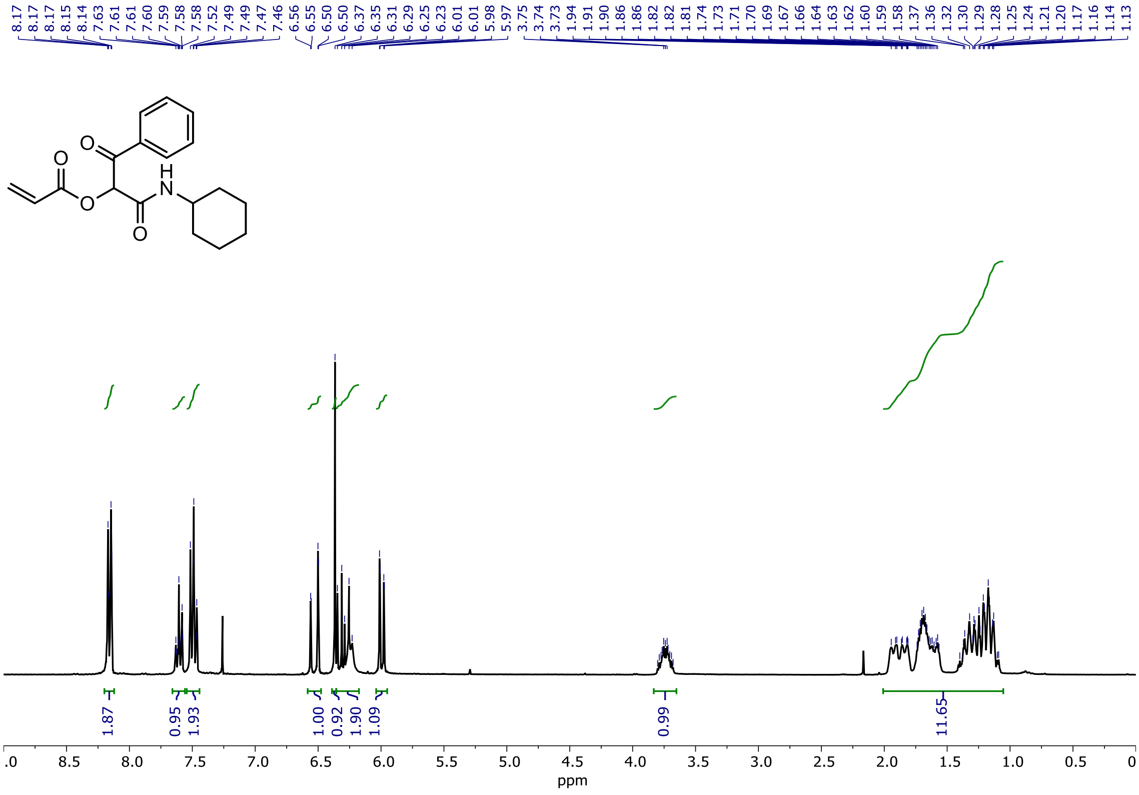

Figure 23. ^{1}H NMR spectrum of **10** (300 MHz, $CDCl_3$).

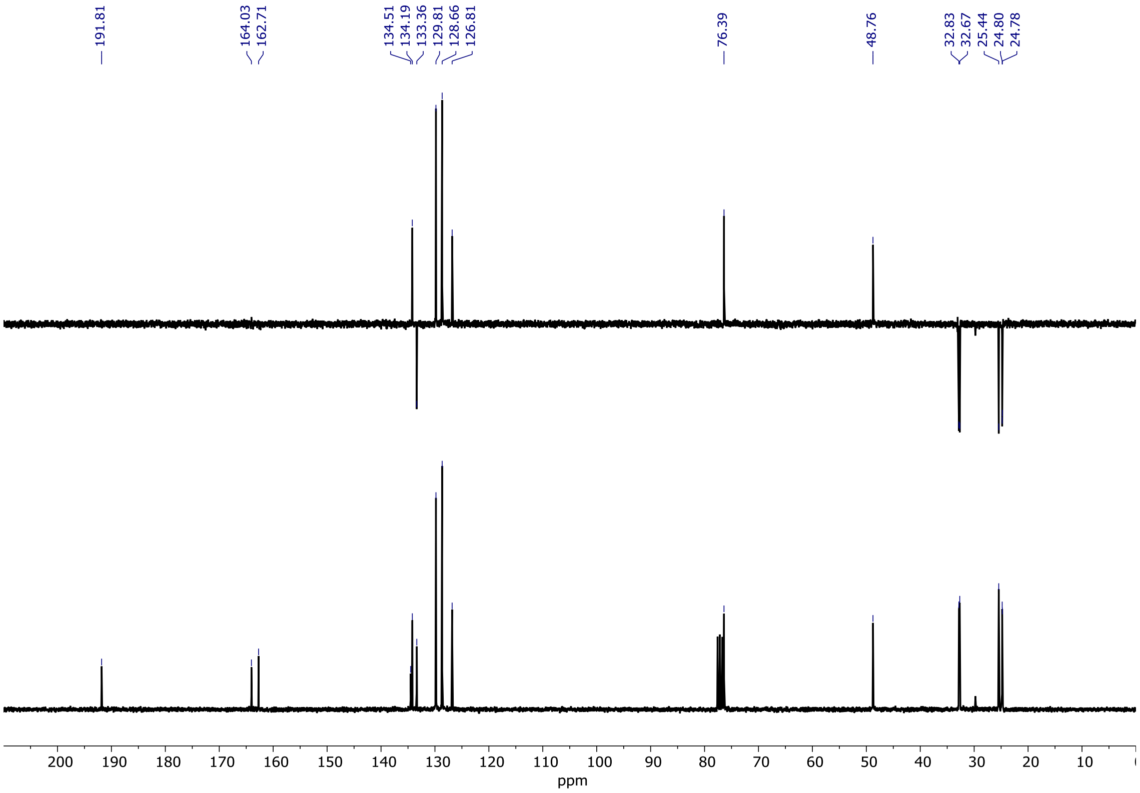

Figure 24. ^{13}C and DEPT-135 NMR spectra of **10** (75 MHz, $CDCl_3$).

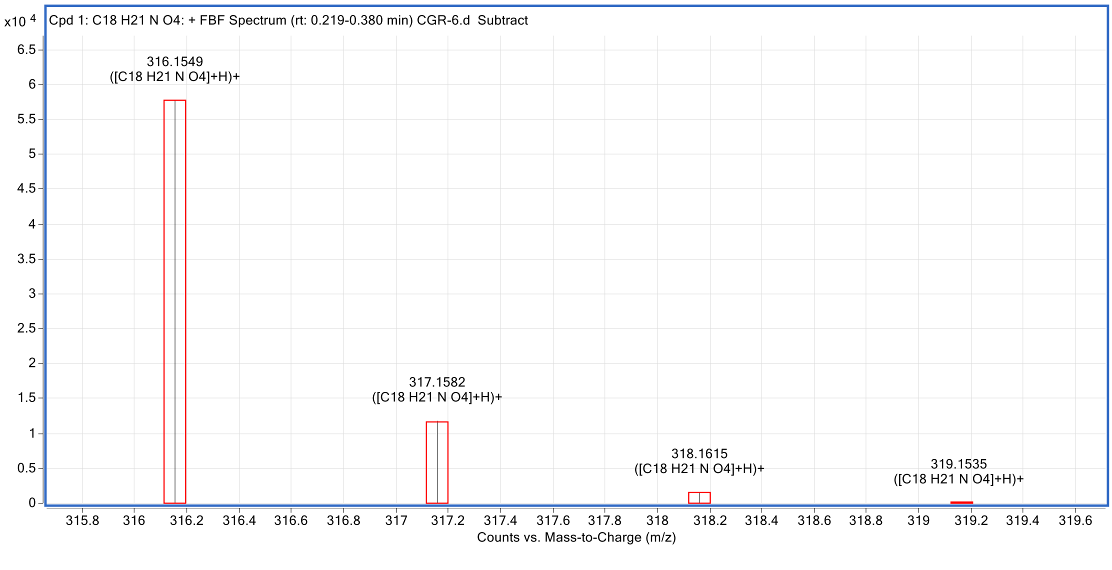

Figure 25. High-resolution mass spectrum of **10**.

2-Benzoyl-*N*-cyclohexyl-5-oxotetrahydrofuran-2-carboxamide (11a)

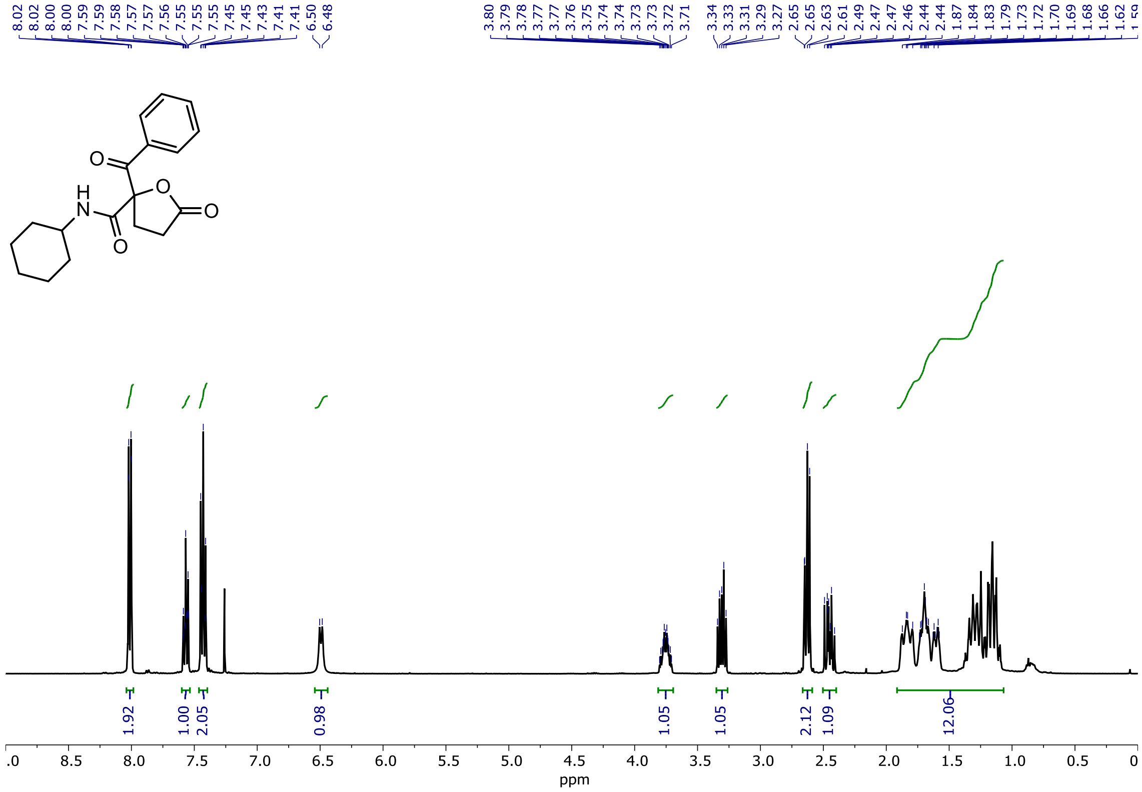

Figure 26. ^{1}H NMR spectrum of **11a** (400 MHz, $CDCl_3$).

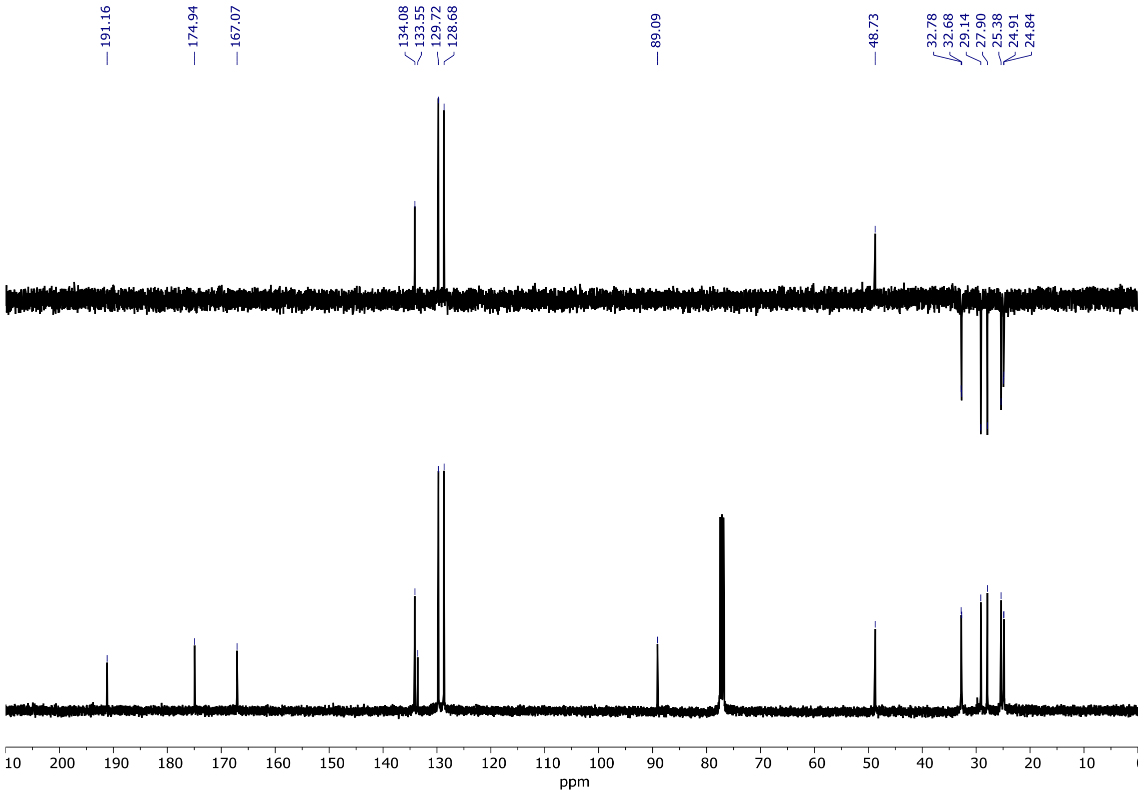

Figure 27. ^{13}C and DEPT-135 NMR spectra of **11a** (100 MHz, $CDCl_3$).

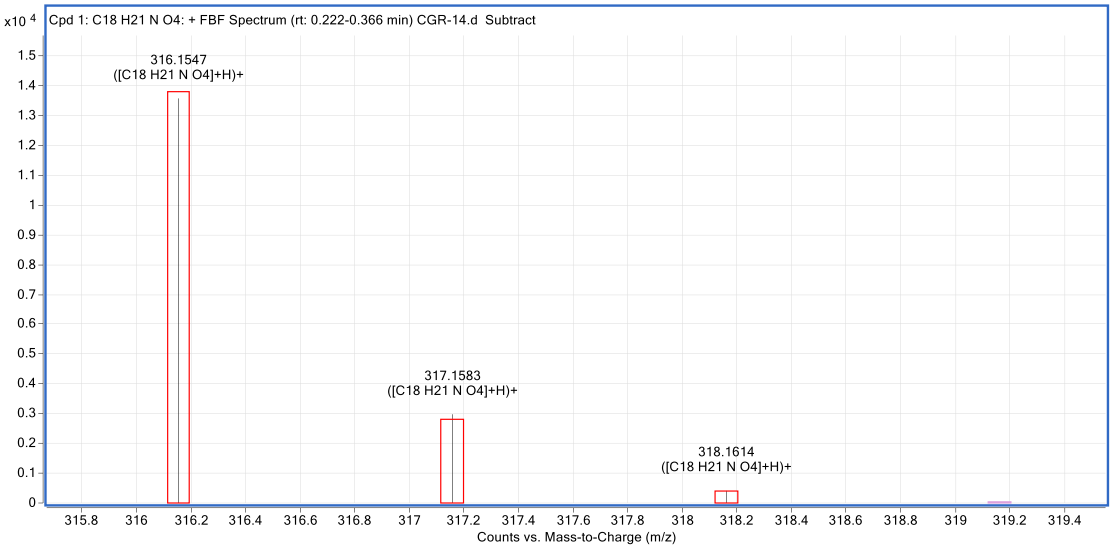

Figure 28. High-resolution mass spectrum of **11a**.

2-Benzoyl-*N*-butyl-5-oxotetrahydrofuran-2-carboxamide (11b)

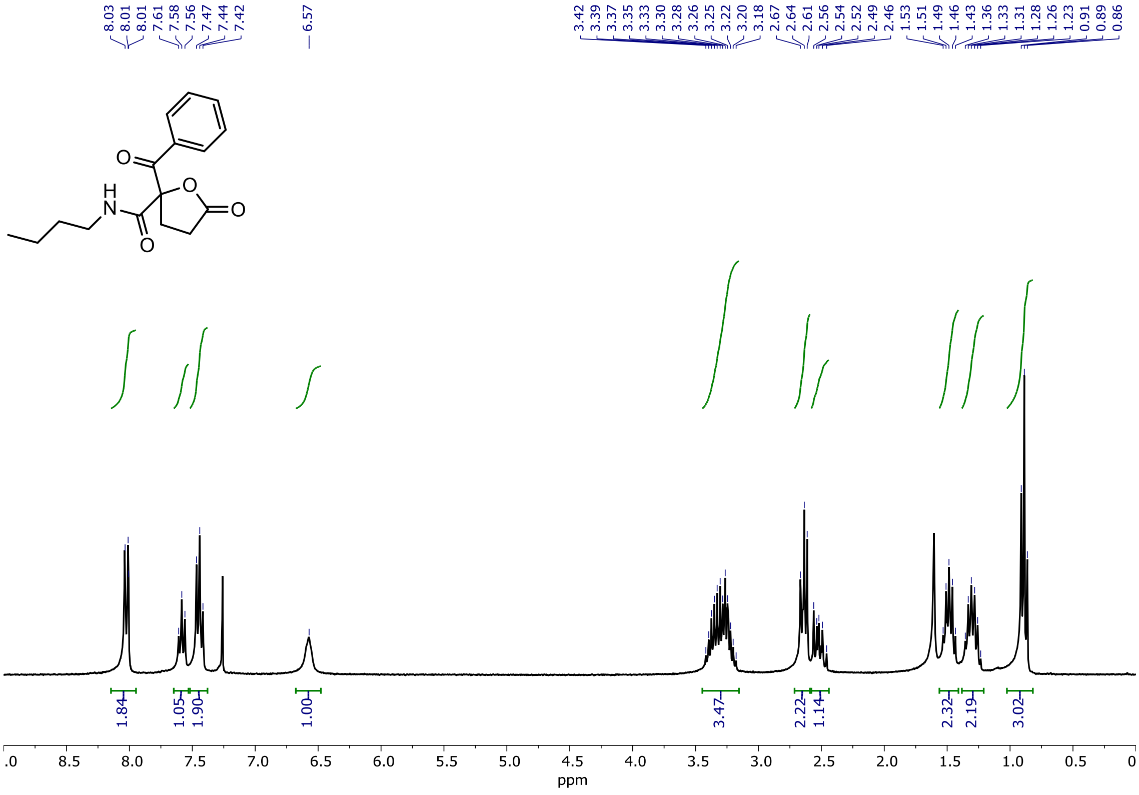

Figure 29. ^{1}H NMR spectrum of **11b** (300 MHz, $CDCl_3$).

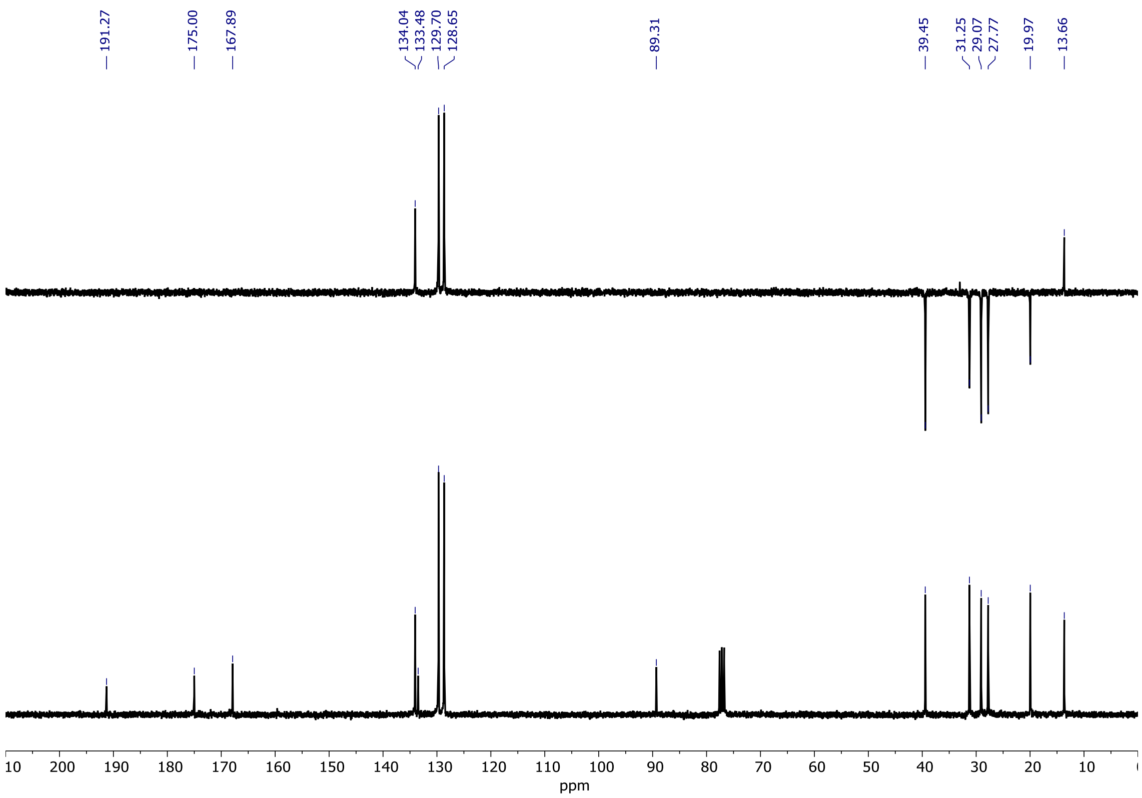

Figure 30. ^{13}C and DEPT-135 NMR spectra of **11b** (75 MHz, $CDCl_3$).

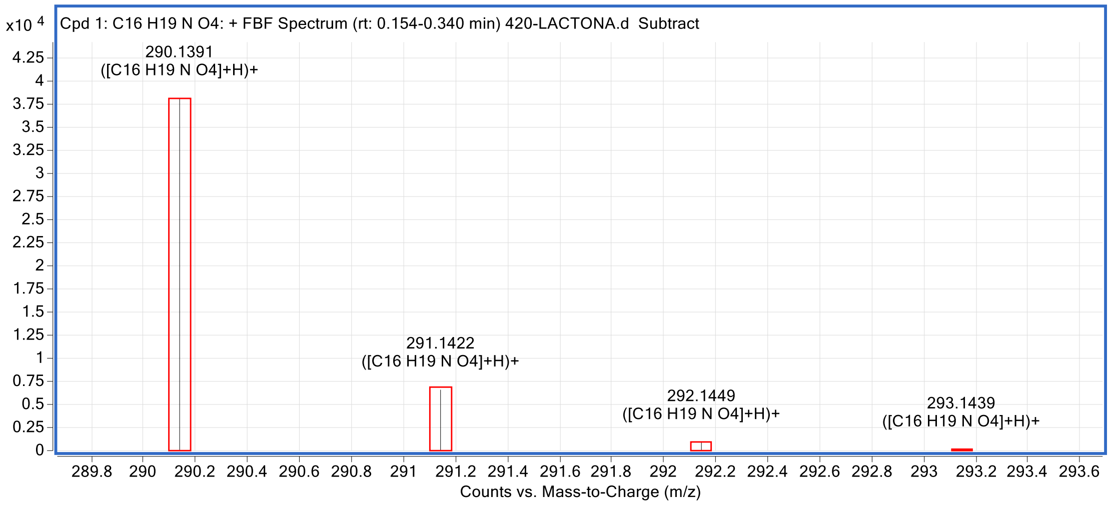

Figure 31. High-resolution mass spectrum of **11b**.

2-Benzoyl-*N*-(*tert*-butyl)-5-oxotetrahydrofuran-2-carboxamide (11c)

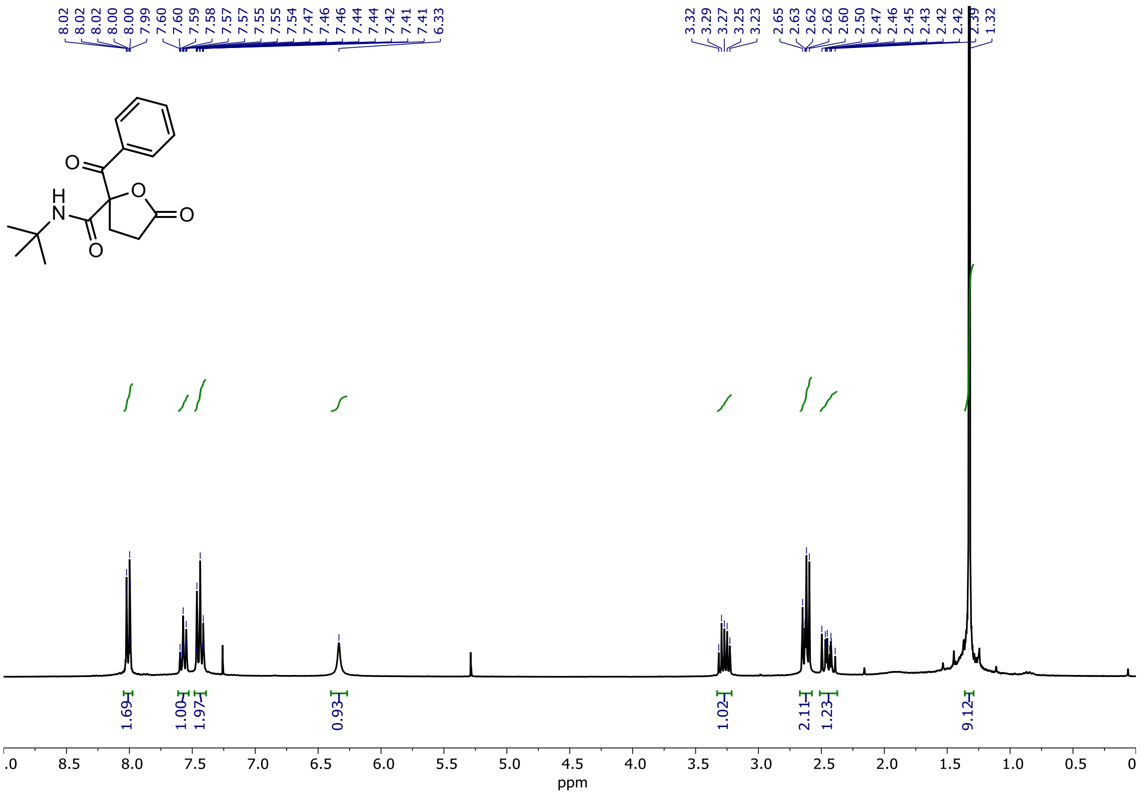

Figure 32. ^{1}H NMR spectrum of **11c** (300 MHz, $CDCl_3$).

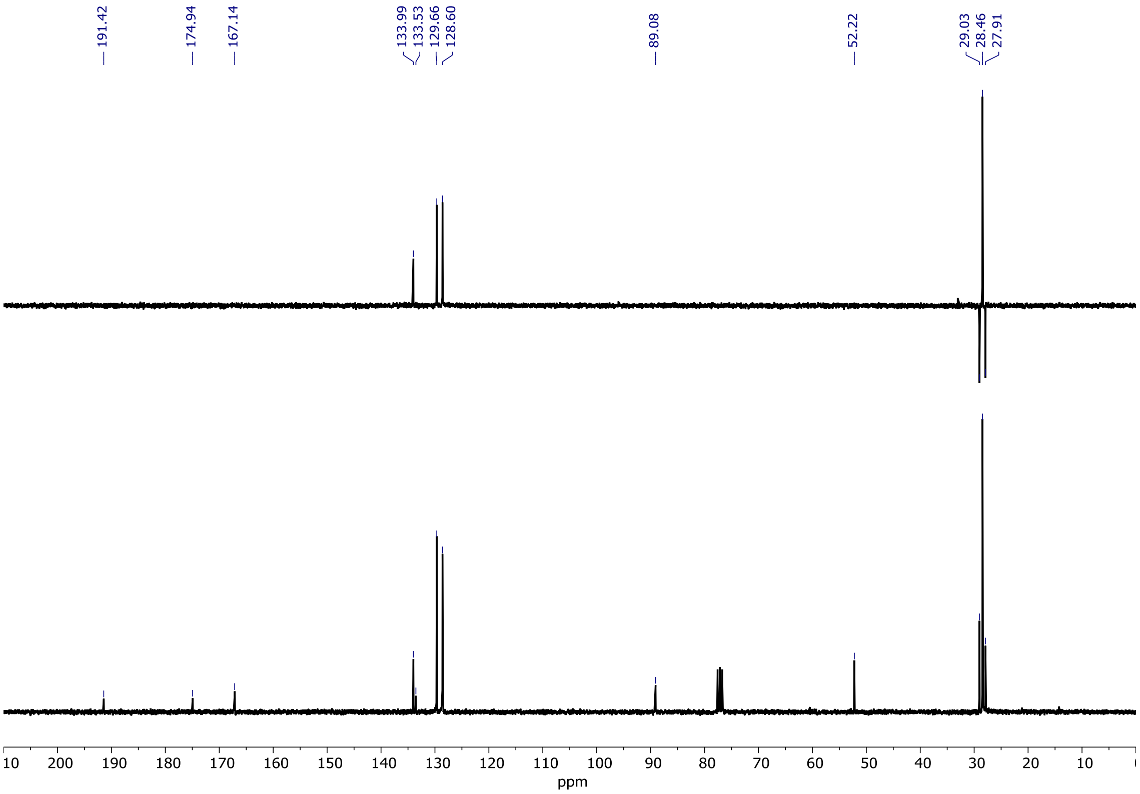

Figure 33. ^{13}C and DEPT-135 NMR spectra of **11c** (75 MHz, $CDCl_3$).

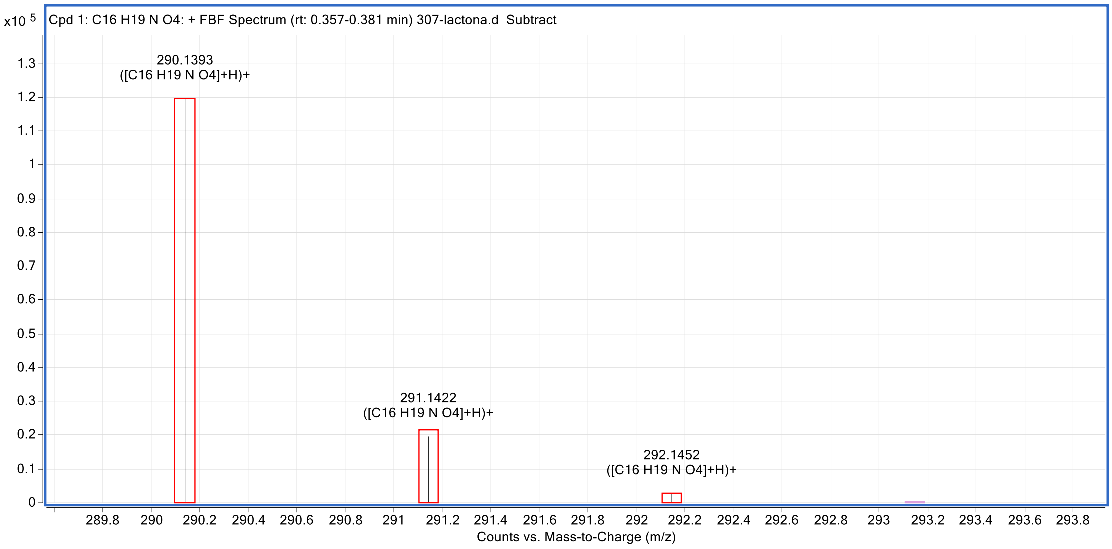

Figure 34. High-resolution mass spectrum of **11c**.

<u>*N*-Cyclohexyl-2-(4-fluorobenzoyl)-5-oxotetrahydrofuran-2-carboxamide (11d)</u>

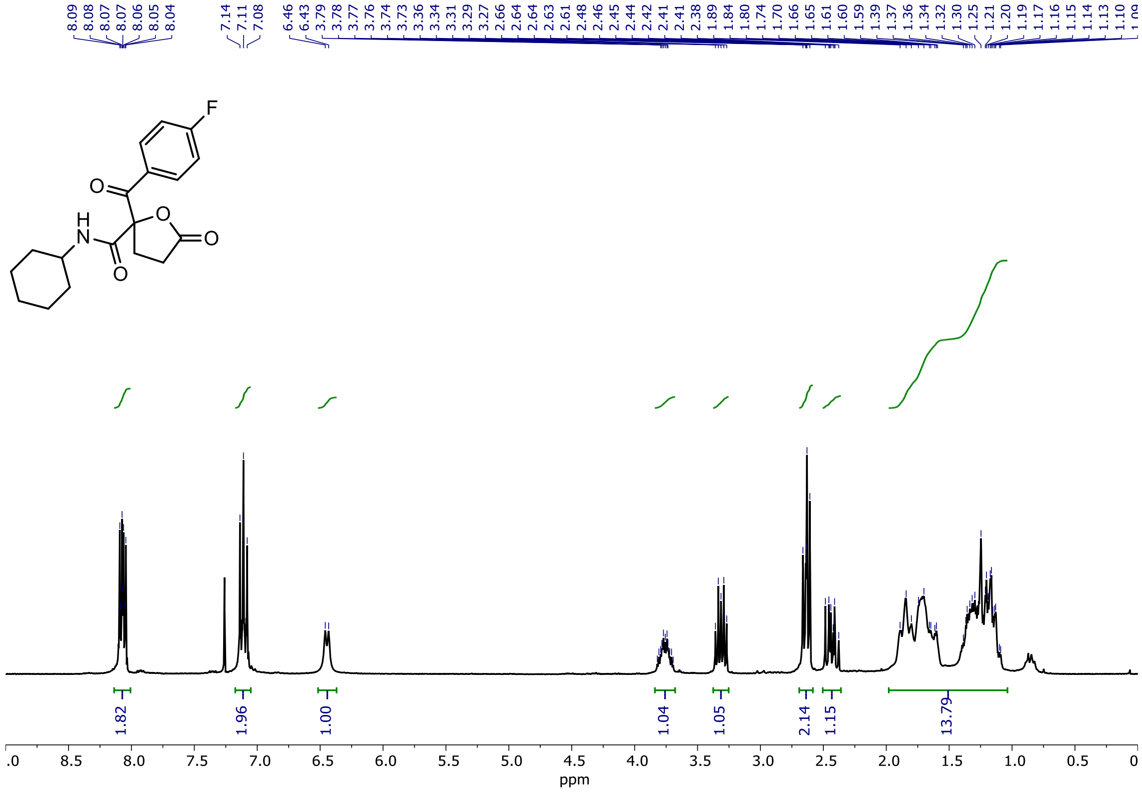

Figure 35. ^{1}H NMR spectrum of **11d** (300 MHz, $CDCl_3$).

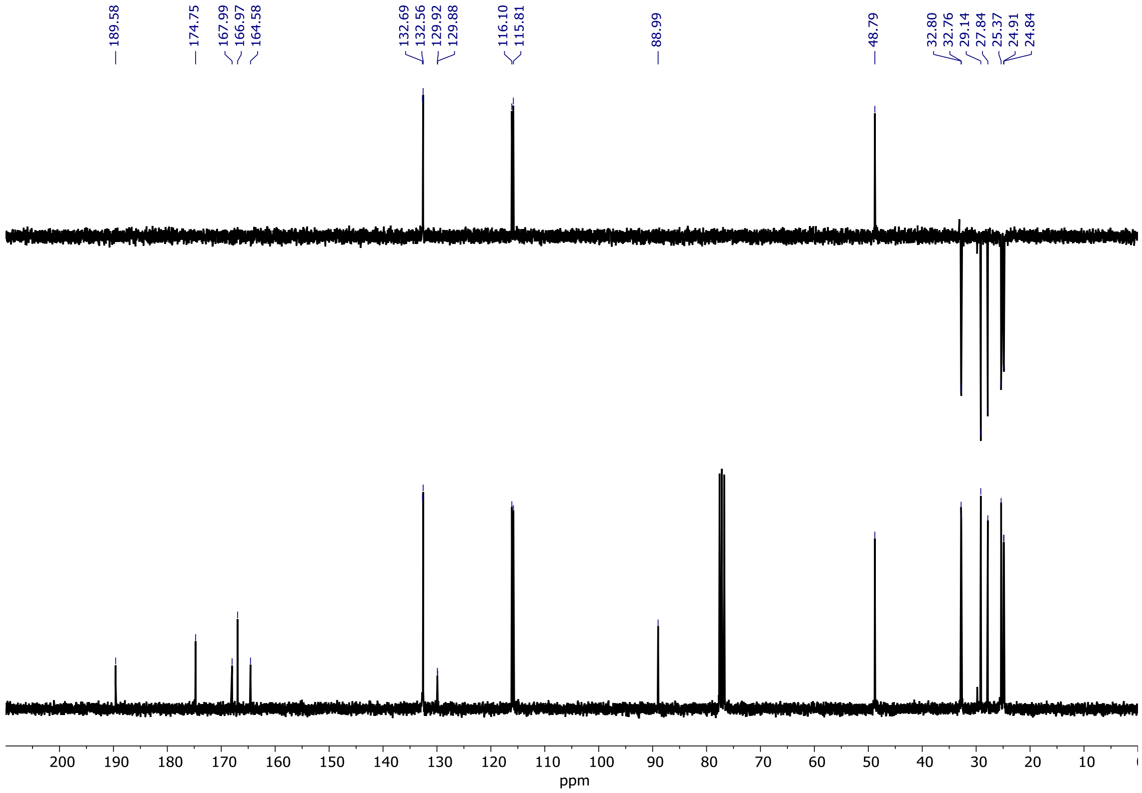

Figure 36. ^{13}C and DEPT-135 NMR spectra of **11d** (75 MHz, $CDCl_3$).

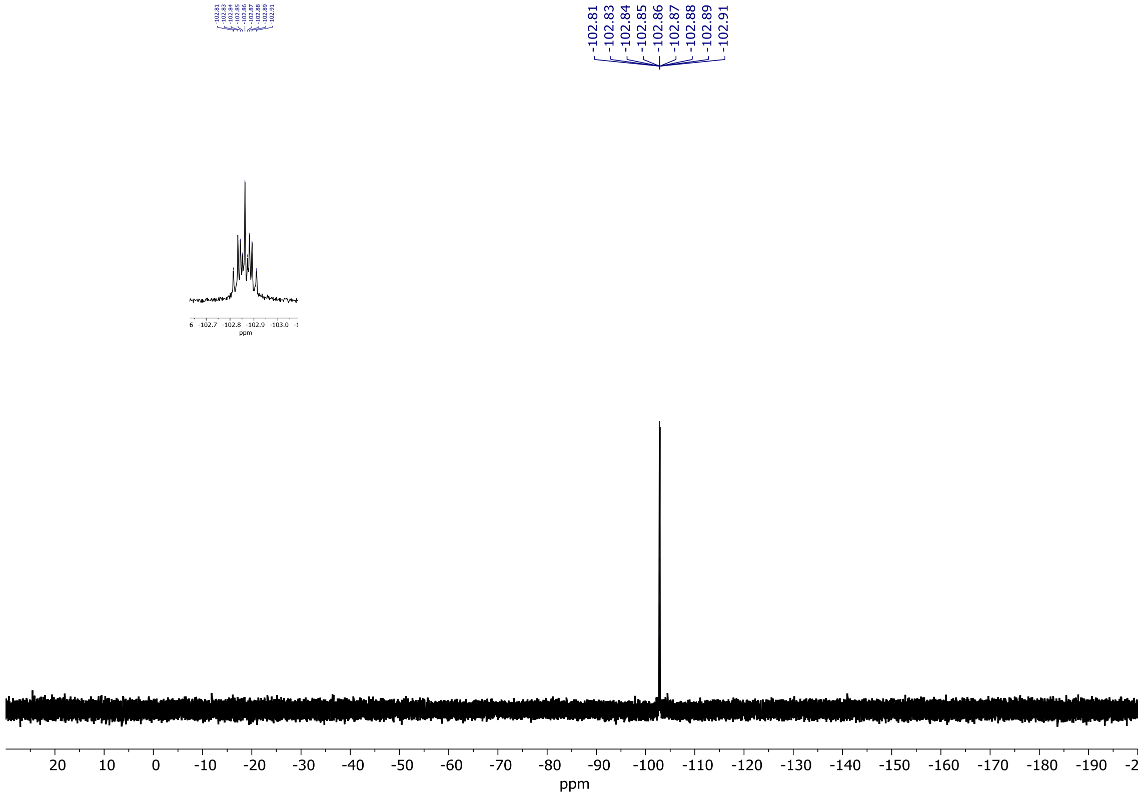

Figure 37. ^{19}F NMR spectrum of **11d** (300 MHz, $CDCl_3$).

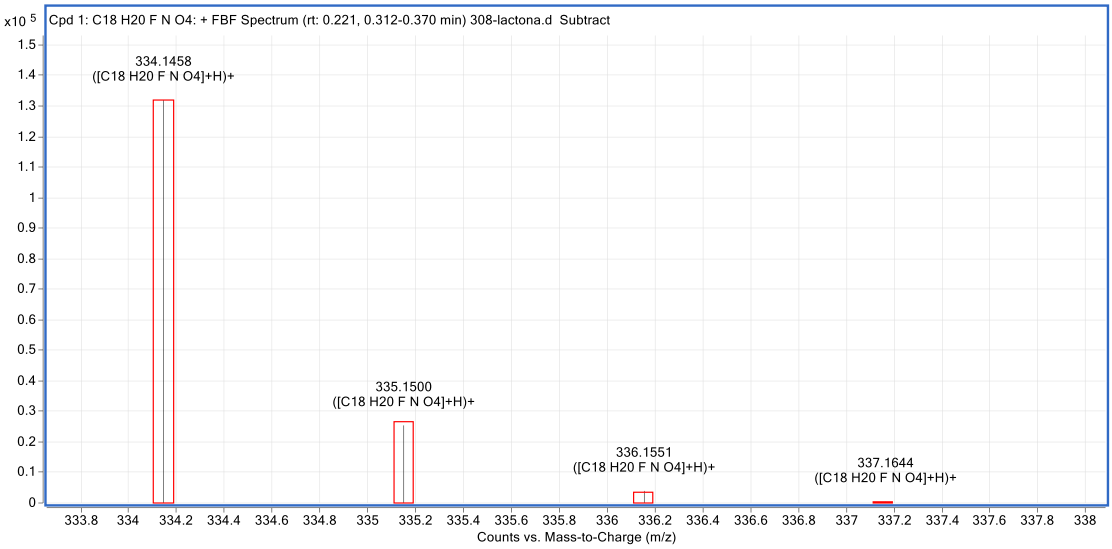

Figure 38. High-resolution mass spectrum of **11d**.

4-Benzoyl-5-(cyclohexylamino)-4-hydroxy-5-oxopentanoic acid (12a)

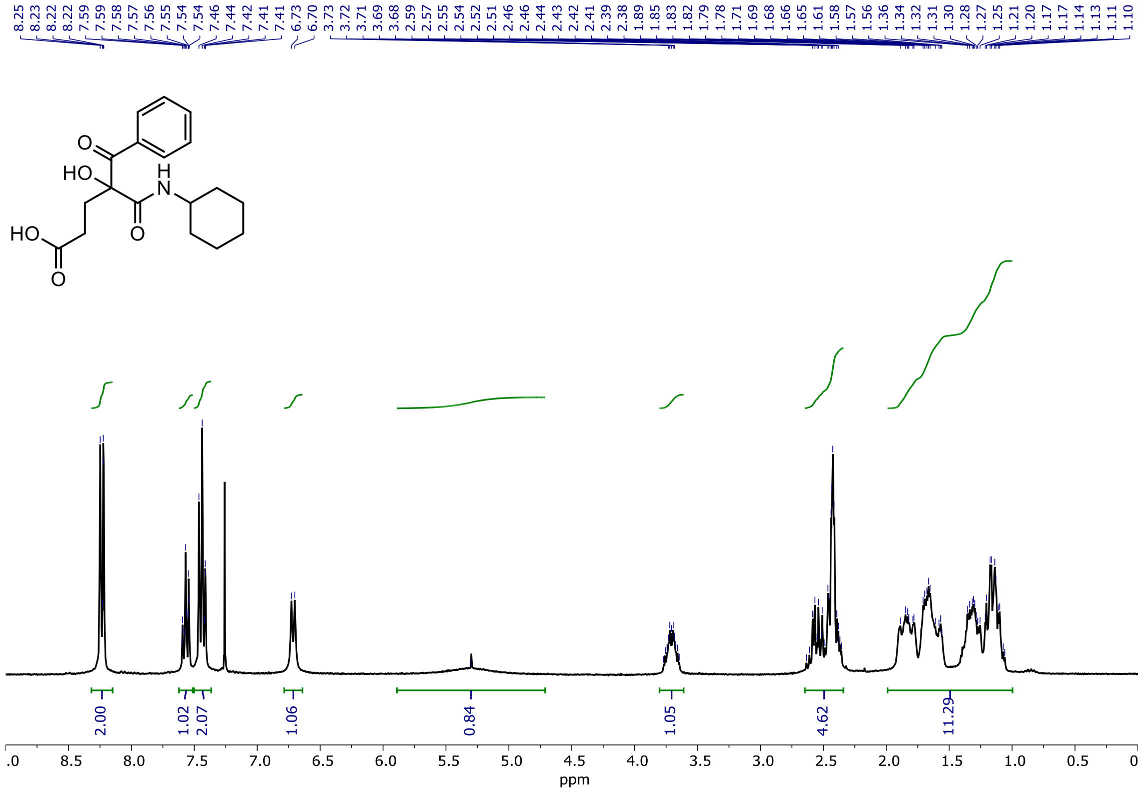

Figure 39. ^{1}H NMR spectrum of **12a** (300 MHz, $CDCl_3$).

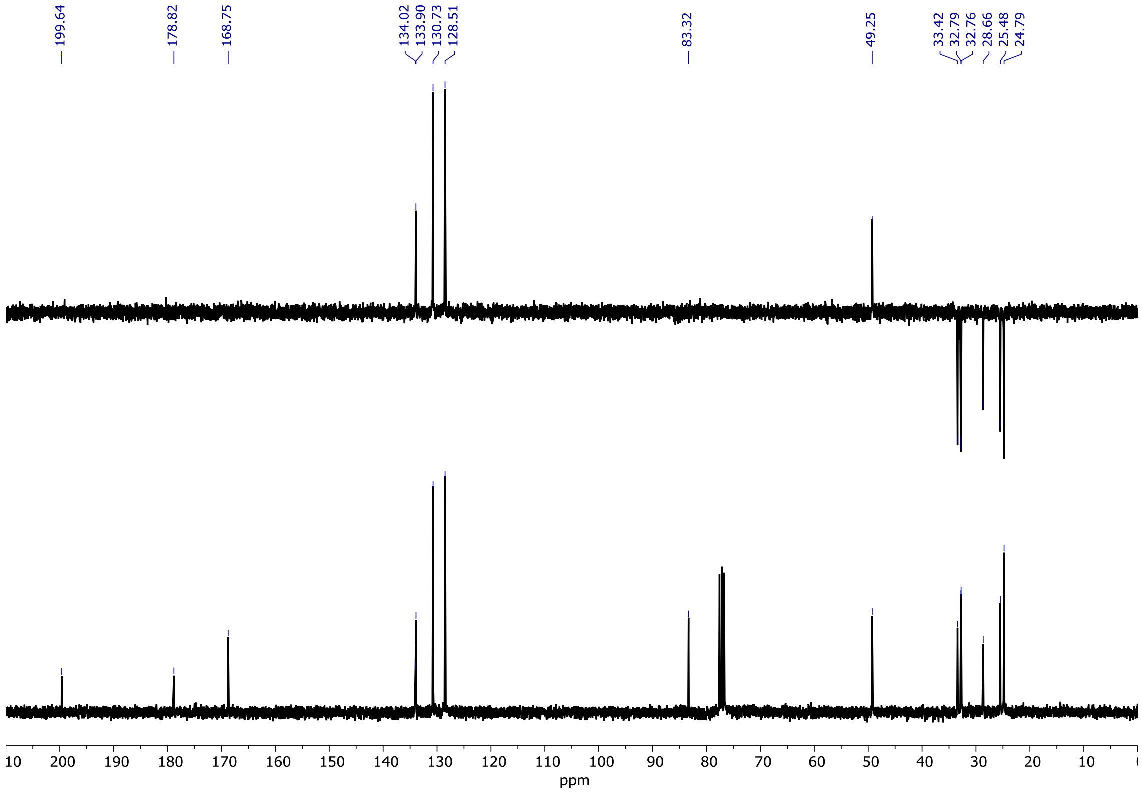

Figure 40. ^{13}C and DEPT-135 NMR spectra of **12a** (75 MHz, $CDCl_3$).

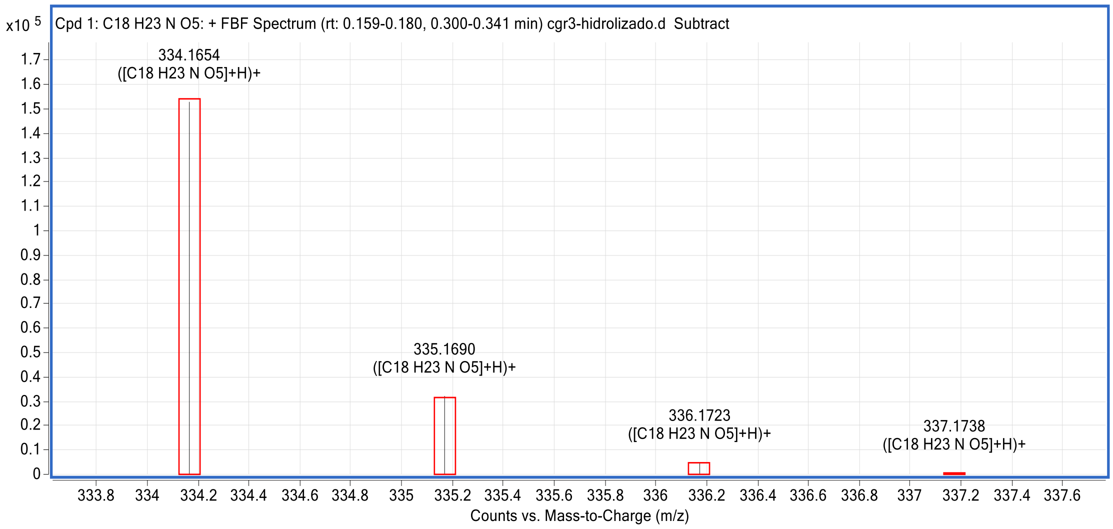

Figure 41. High-resolution mass spectrum of **12a**.

4-Benzoyl-5-(butylamino)-4-hydroxy-5-oxopentanoic acid (12b)

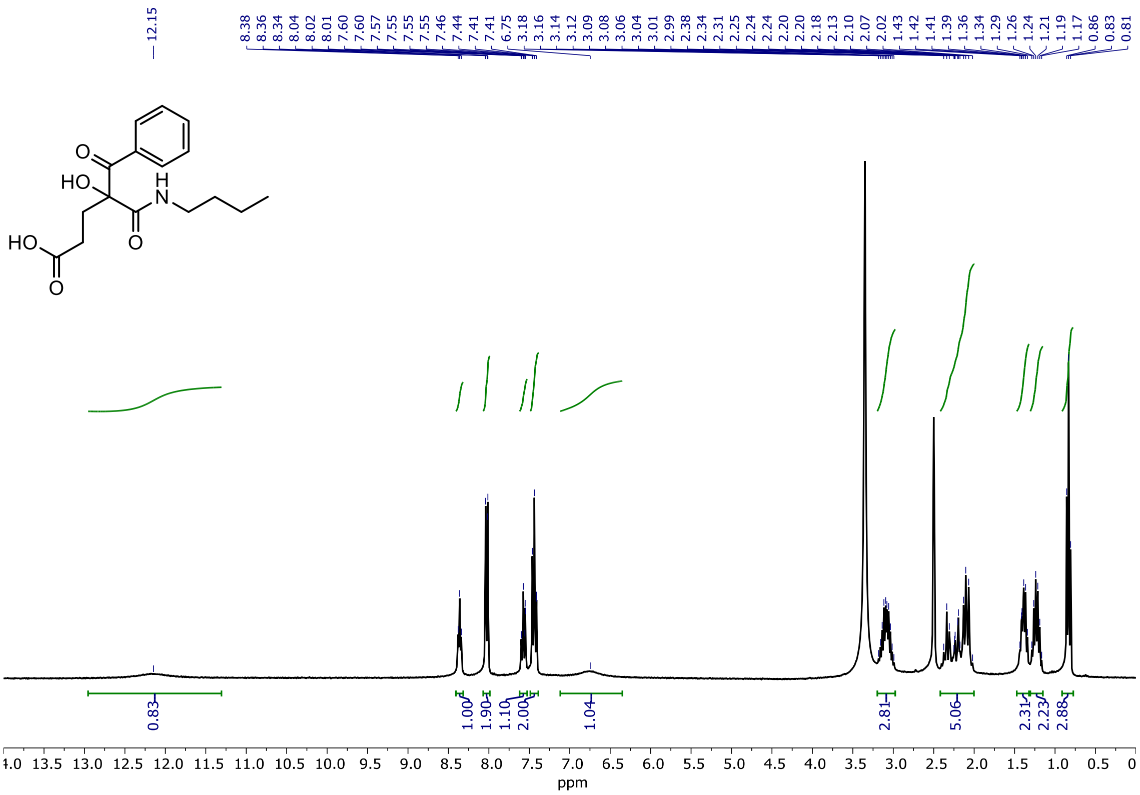

Figure 42. ^{1}H NMR spectrum of **12b** (300 MHz, DMSO-d_6).

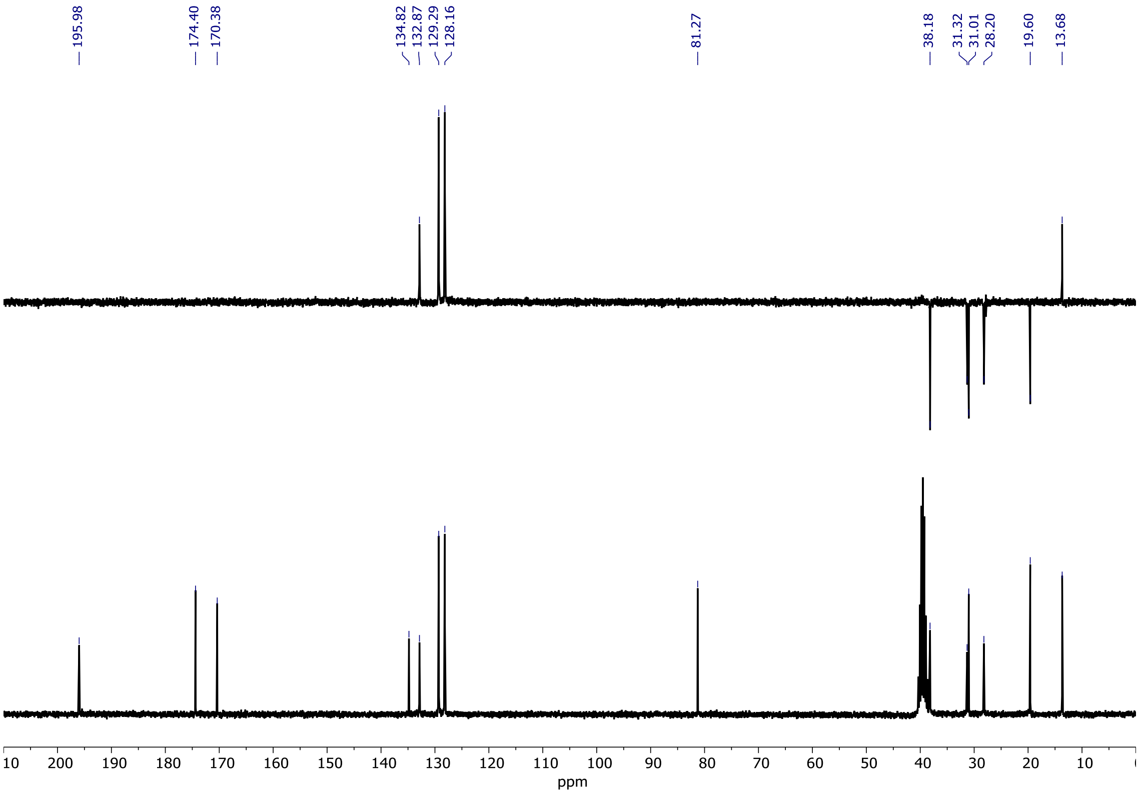

Figure 43. ^{13}C and DEPT-135 NMR spectra of **12b** (75 MHz, DMSO-d_6).

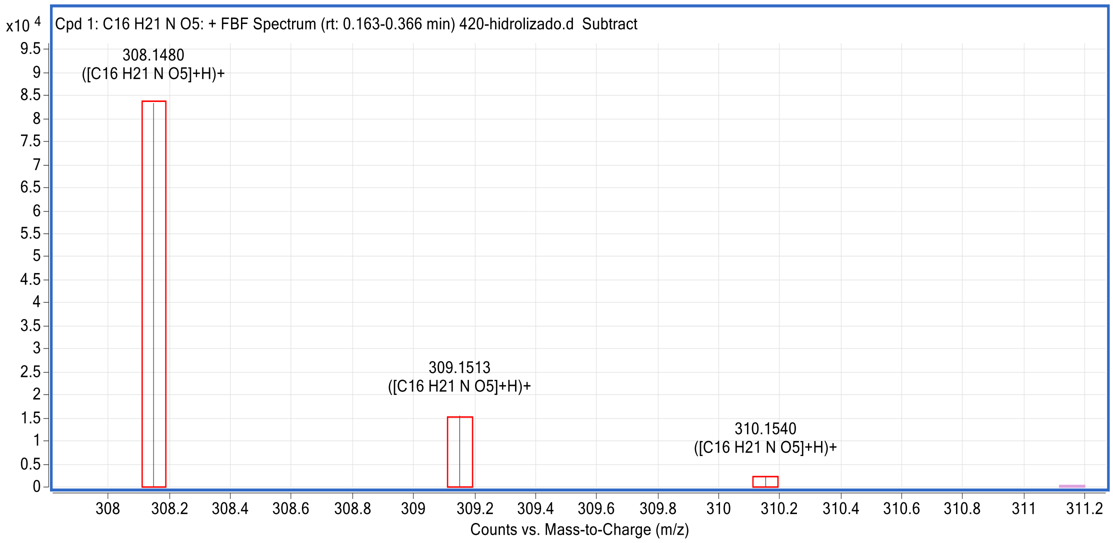

Figure 44. High-resolution mass spectrum of **12b**.

4-Benzoyl-5-(*tert*-butylamino)-4-hydroxy-5-oxopentanoic acid (12c)

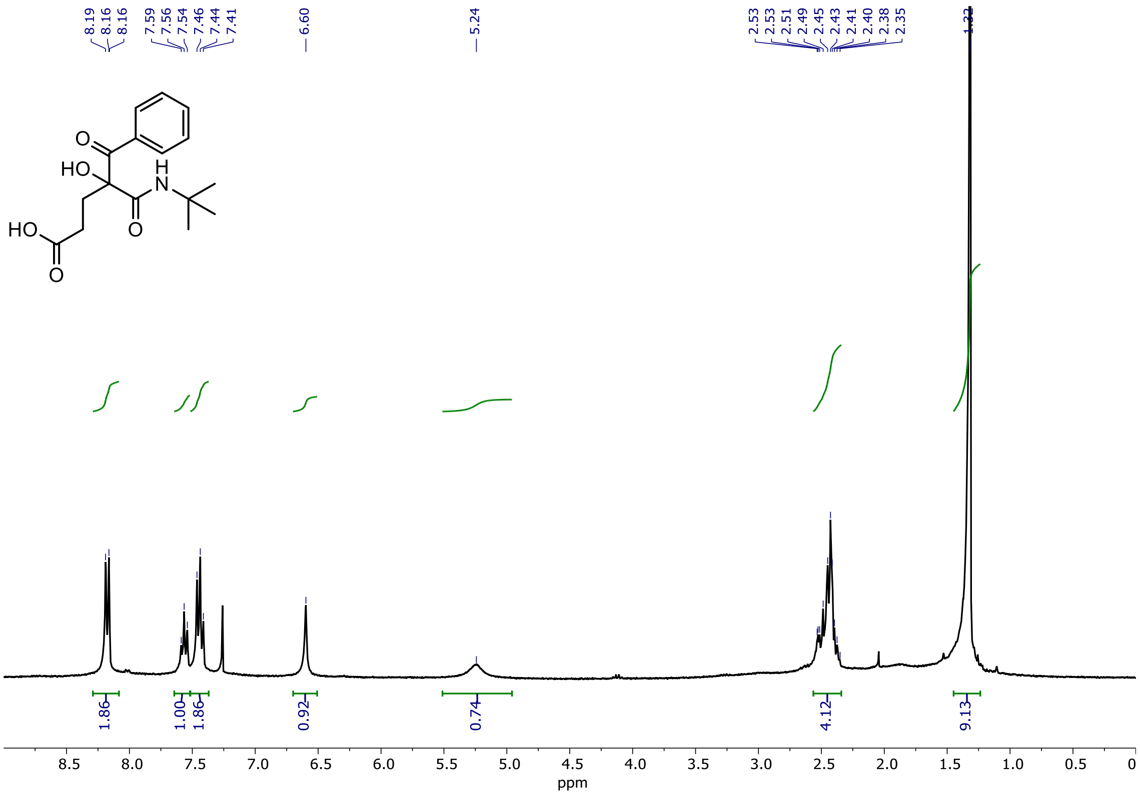

Figure 45. ^{1}H NMR spectrum of **12c** (300 MHz, $CDCl_3$).

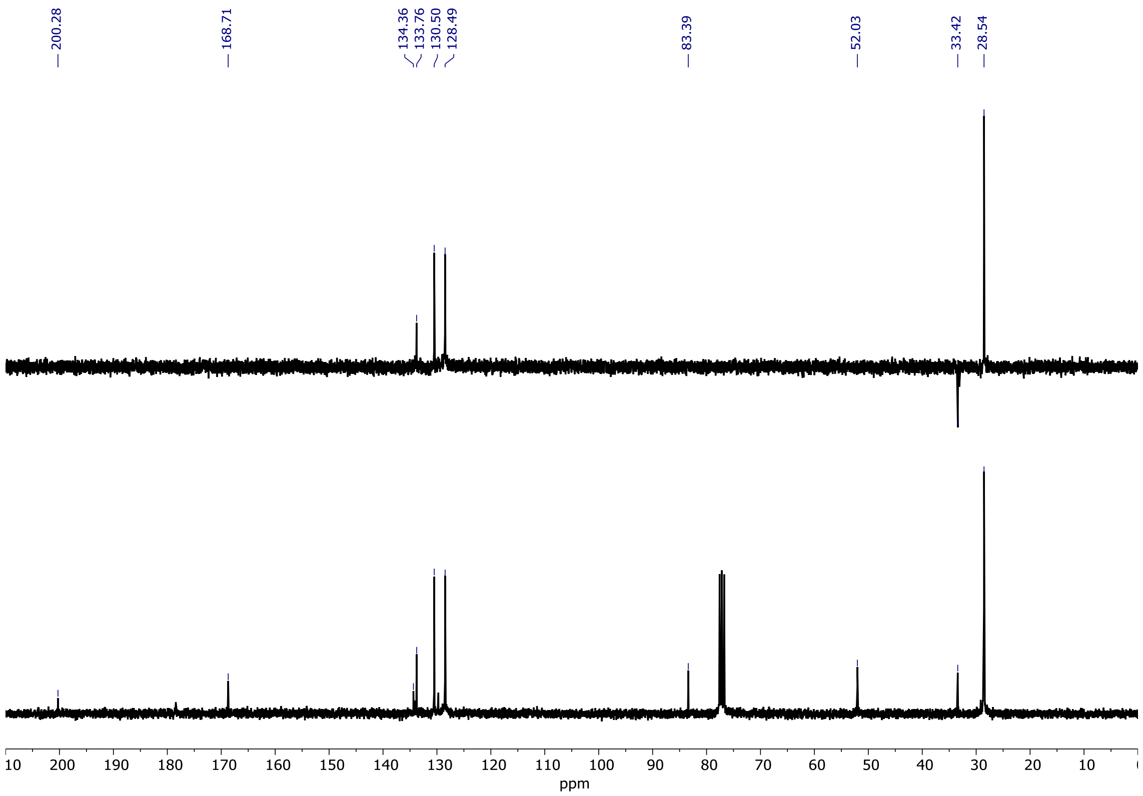

Figure 46. ^{13}C and DEPT-135 NMR spectra of **12c** (75 MHz, $CDCl_3$).

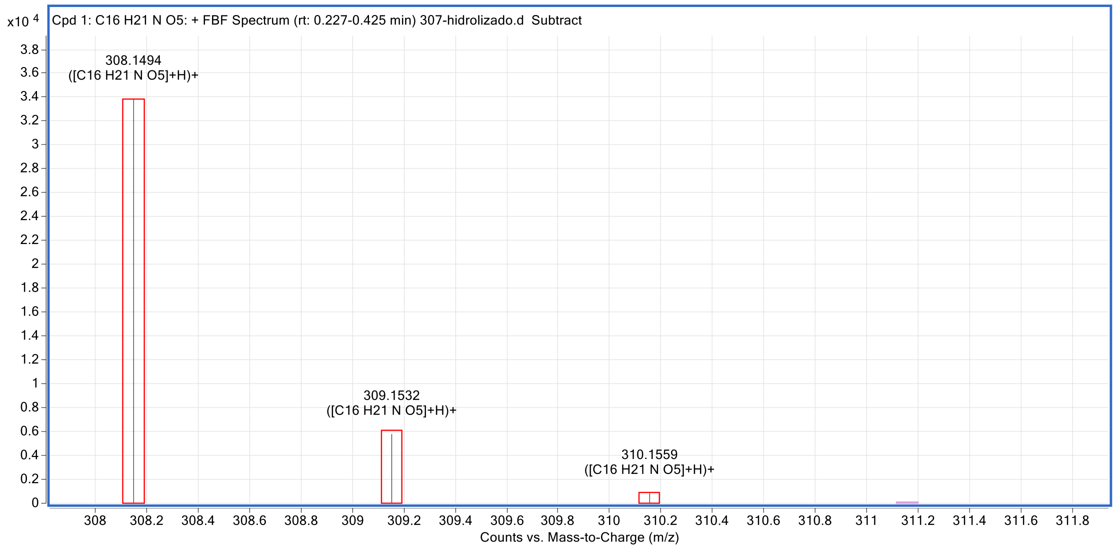

Figure 47. High-resolution mass spectrum of **12c**.

5-(Cyclohexylamino)-4-(4-fluorobenzoyl)-4-hydroxy-5-oxopentanoic acid (12d)

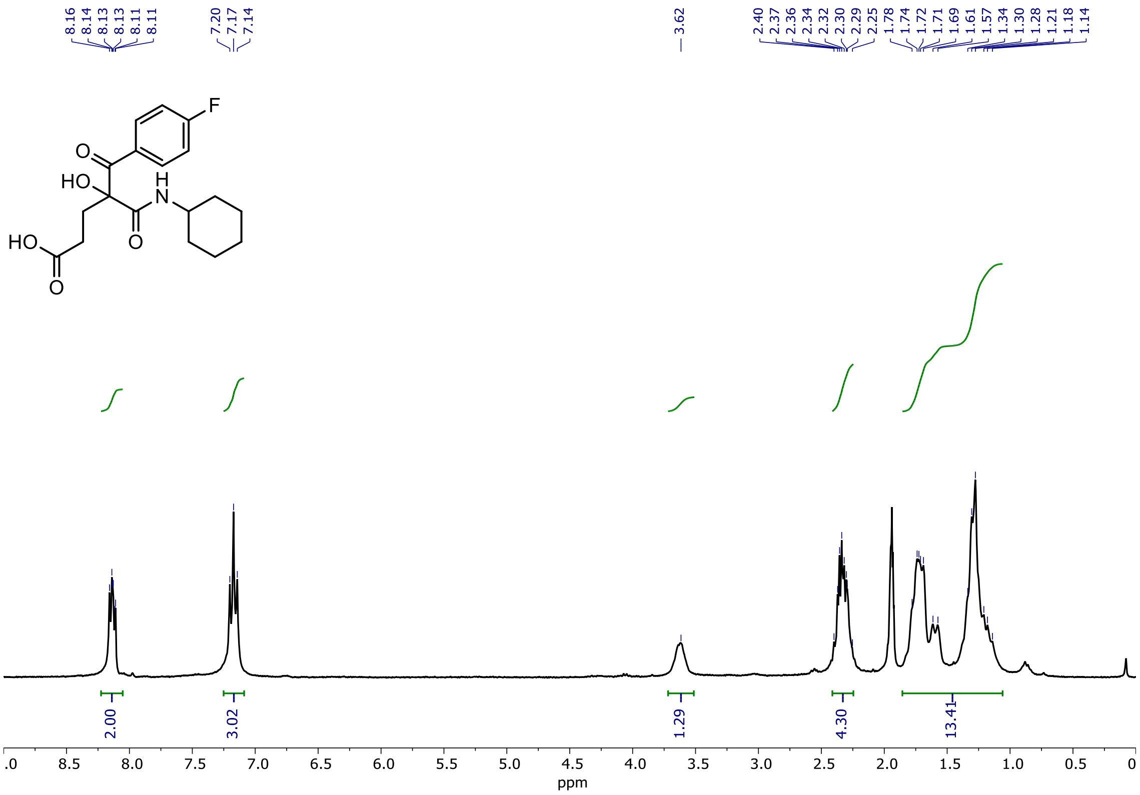

Figure 48. ^{1}H NMR spectrum of **12d** (300 MHz, CD_3CN).

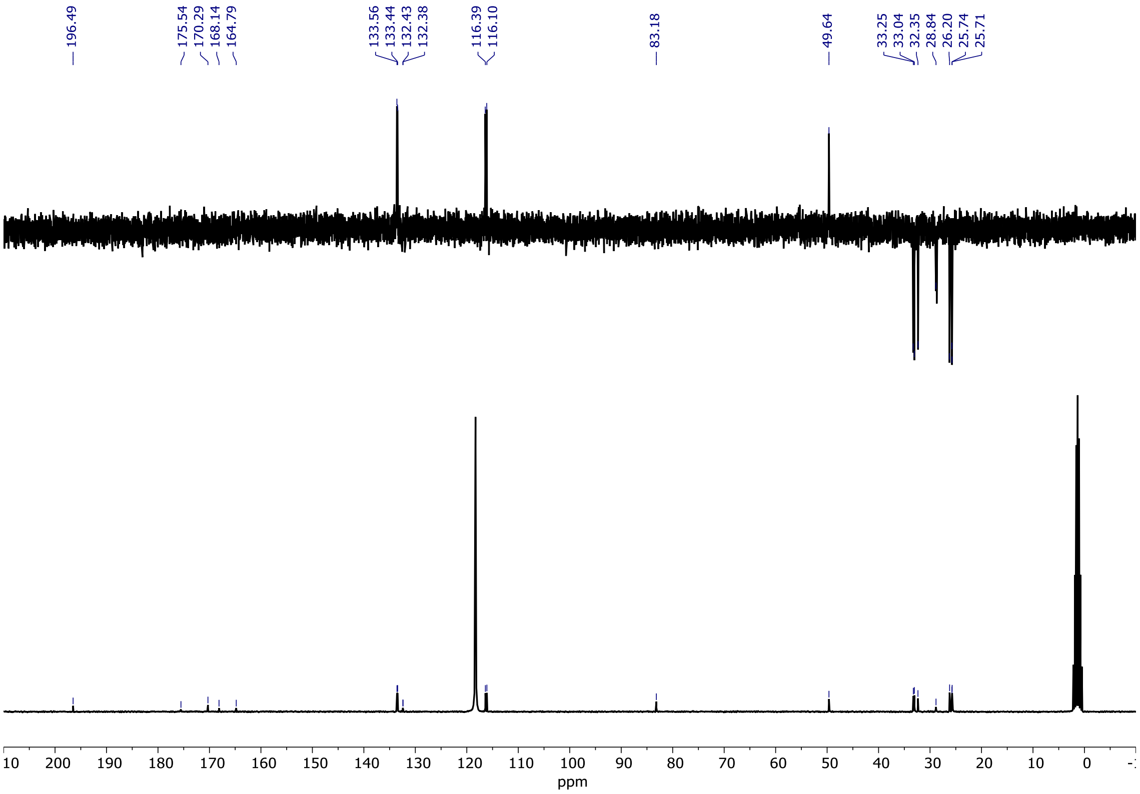

Figure 49. ^{13}C and DEPT-135 NMR spectra of **12d** (75 MHz, CD_3CN).

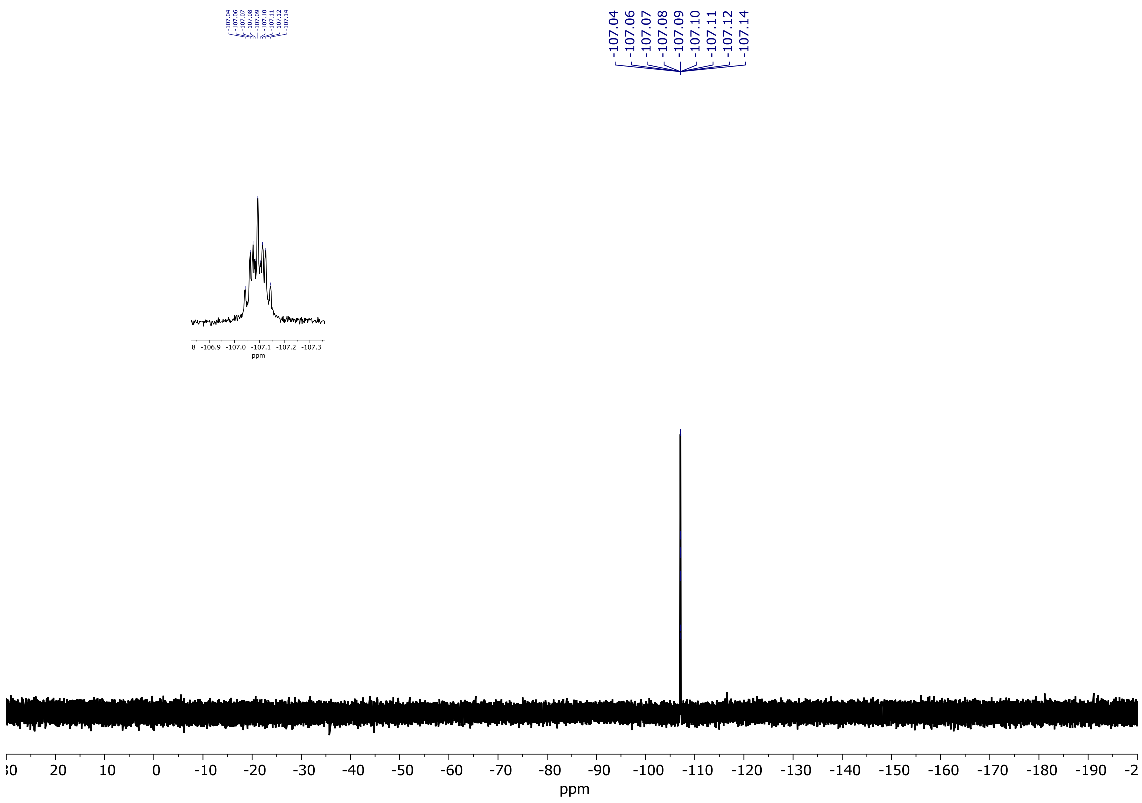

Figure 50. ^{19}F NMR spectrum of **12d** (300 MHz, CD_3CN).

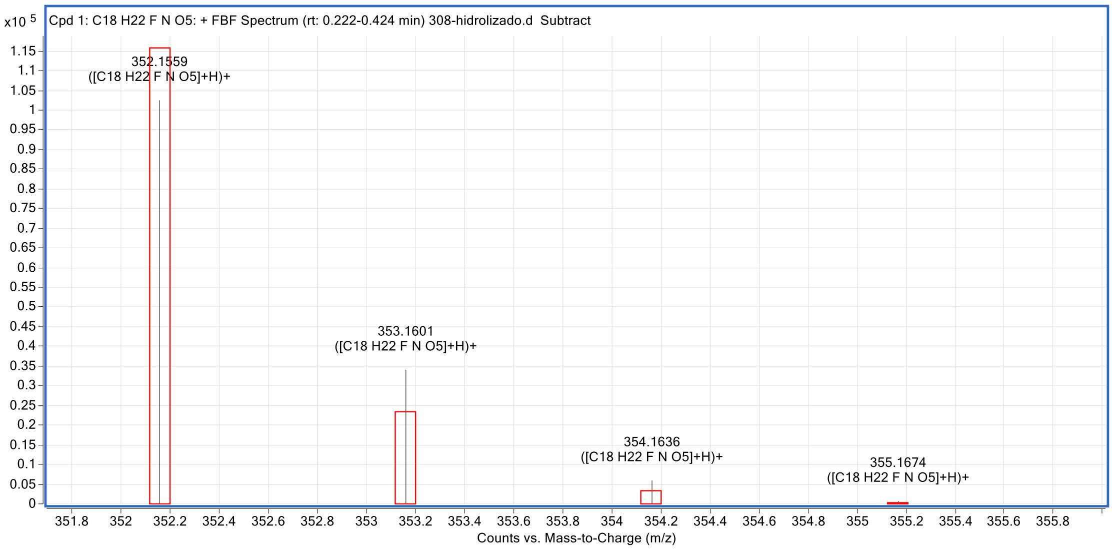

Figure 51. High-resolution mass spectrum of **12d**.

Methyl 4-benzoyl-5-(cyclohexylamino)-4-hydroxy-5-oxopentanoate (13a)

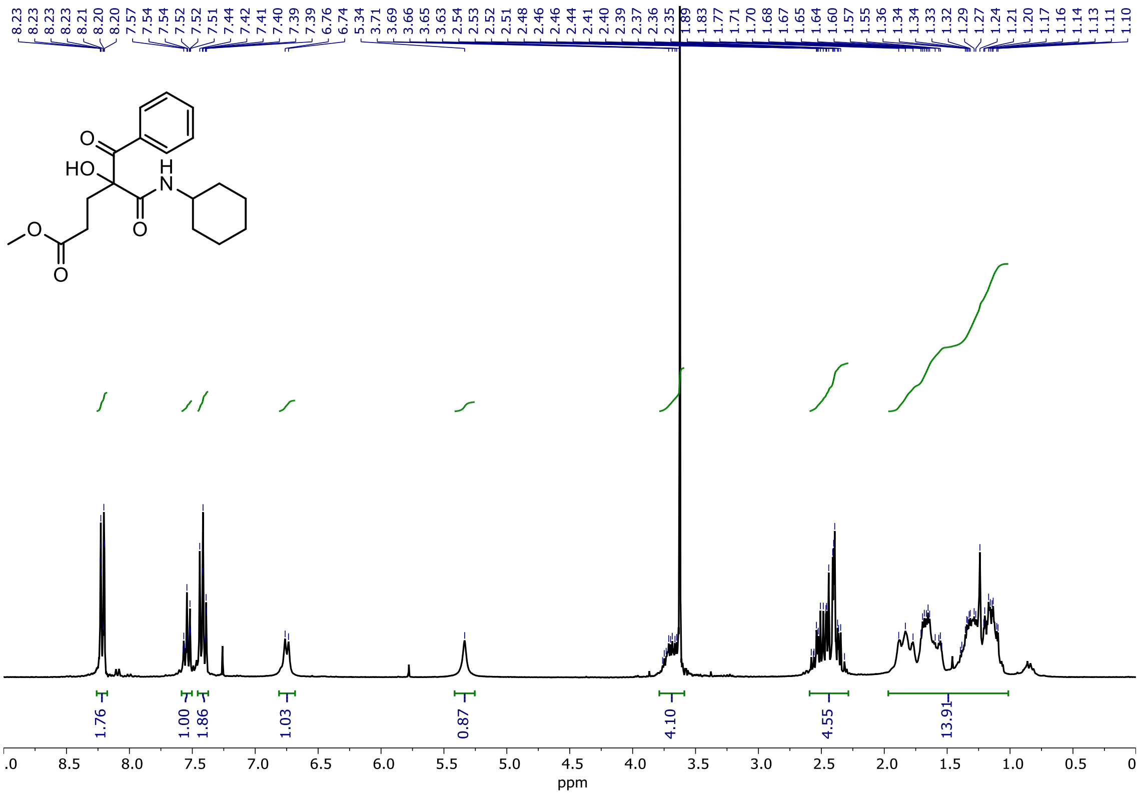

Figure 52. ^{1}H NMR spectrum of **13a** (300 MHz, $CDCl_3$).

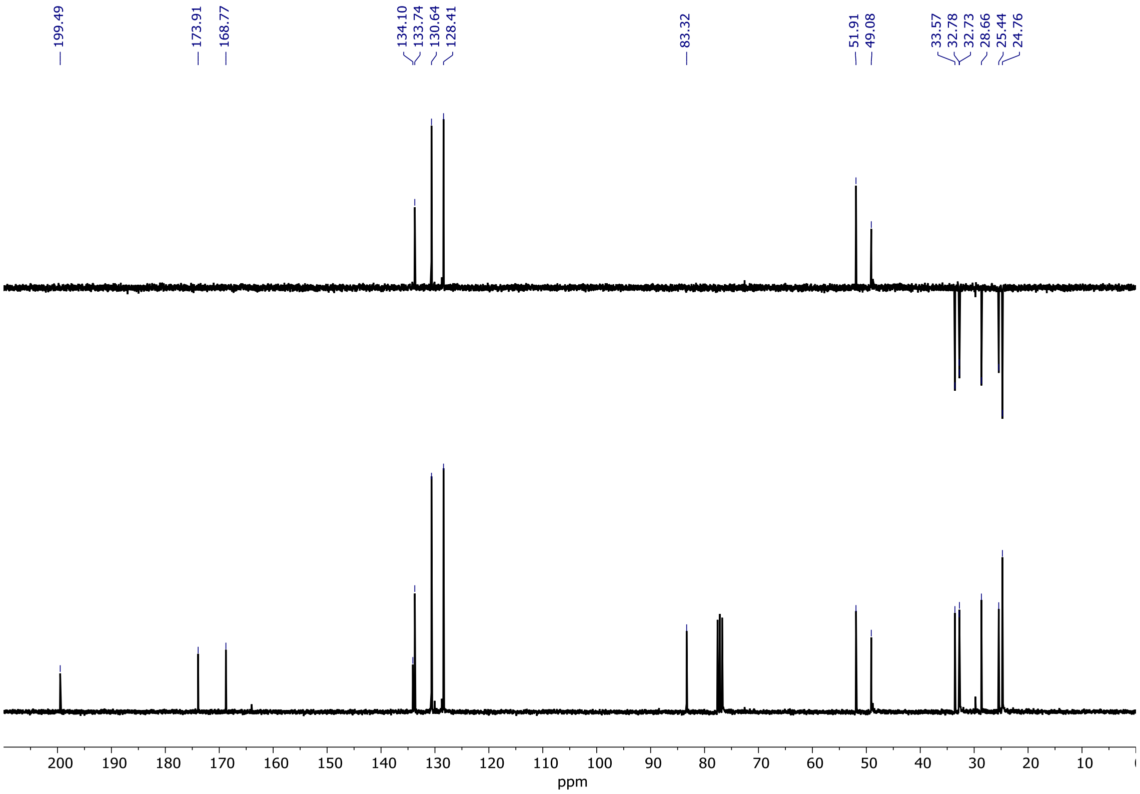

Figure 53. ^{13}C and DEPT-135 NMR spectra of **13a** (75 MHz, $CDCl_3$).

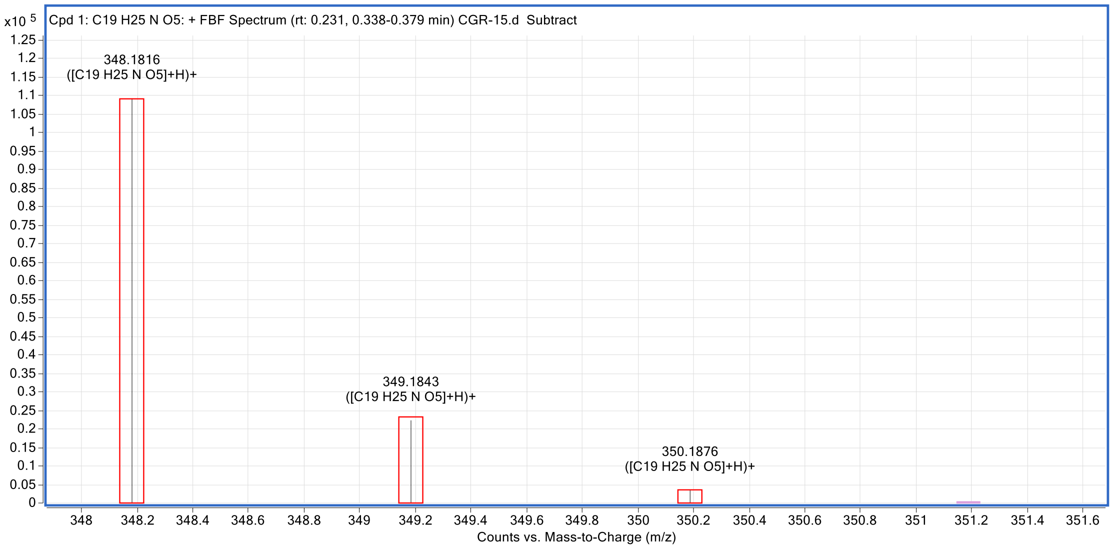

Figure 54. High-resolution mass spectrum of **13a**.

Methyl 4-benzoyl-5-(butylamino)-4-hydroxy-5-oxopentanoate (13b)

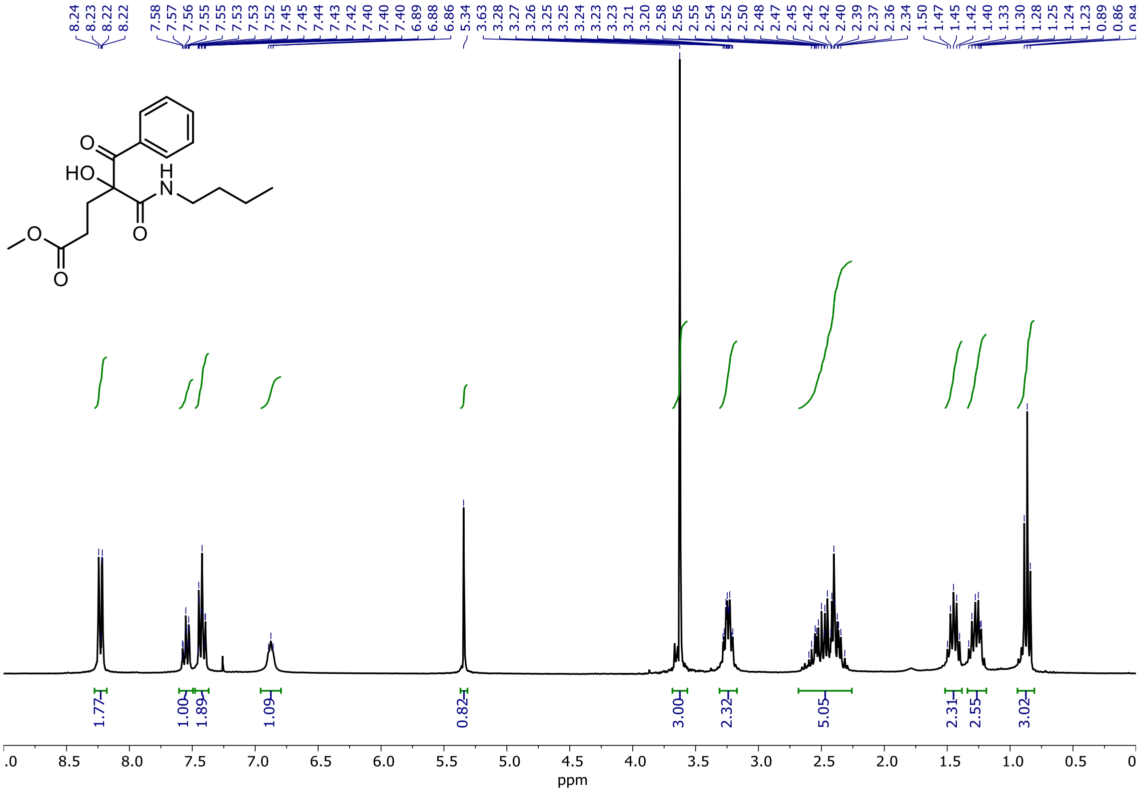

Figure 55. ^{1}H NMR spectrum of **13b** (300 MHz, $CDCl_3$).

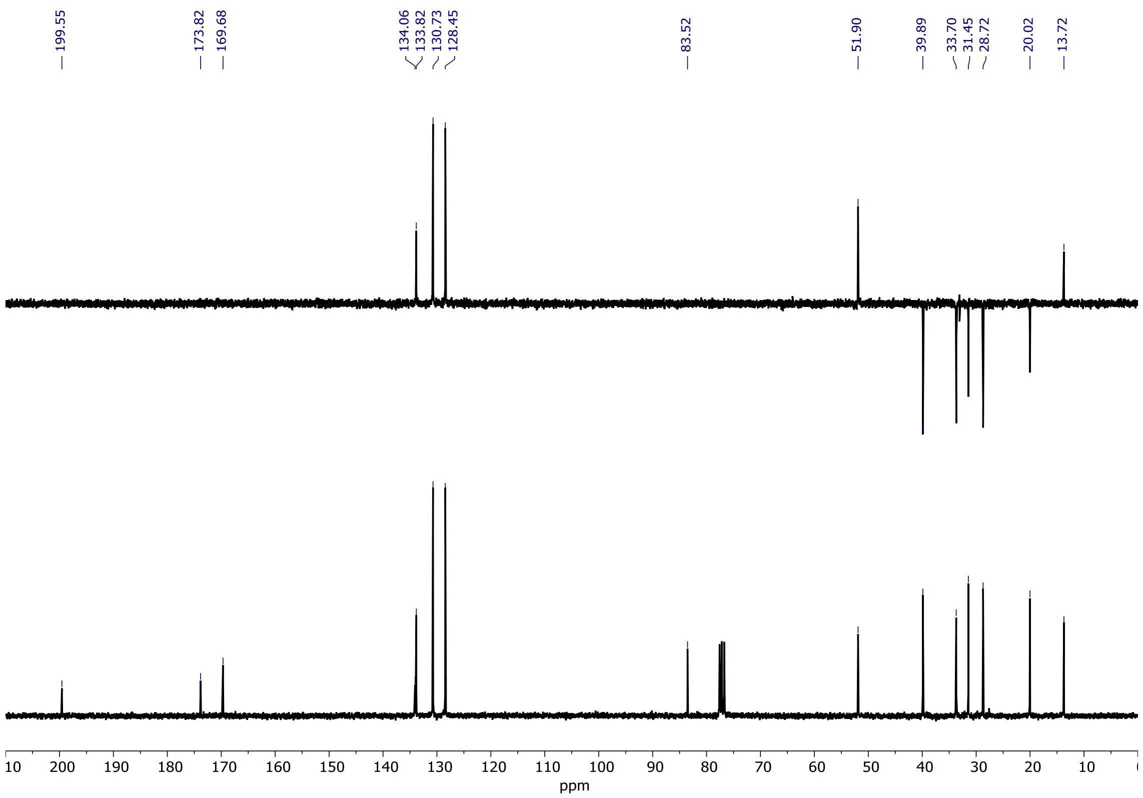

Figure 56. ^{13}C and DEPT-135 NMR spectra of **13b** (75 MHz, $CDCl_3$).

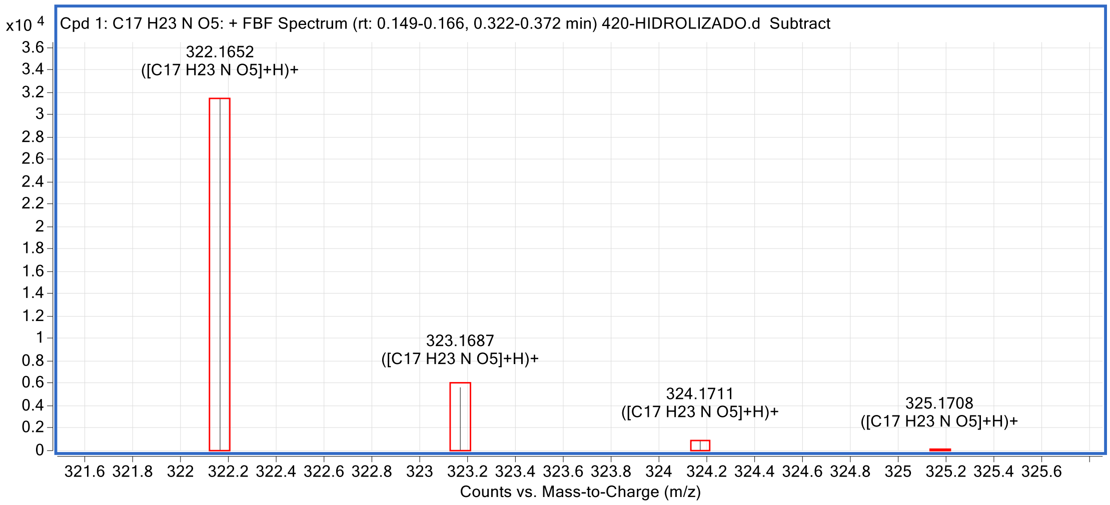

Figure 57. High-resolution mass spectrum of **13b**.

Methyl 4-benzoyl-5-(*tert*-butylamino)-4-hydroxy-5-oxopentanoate (13c)

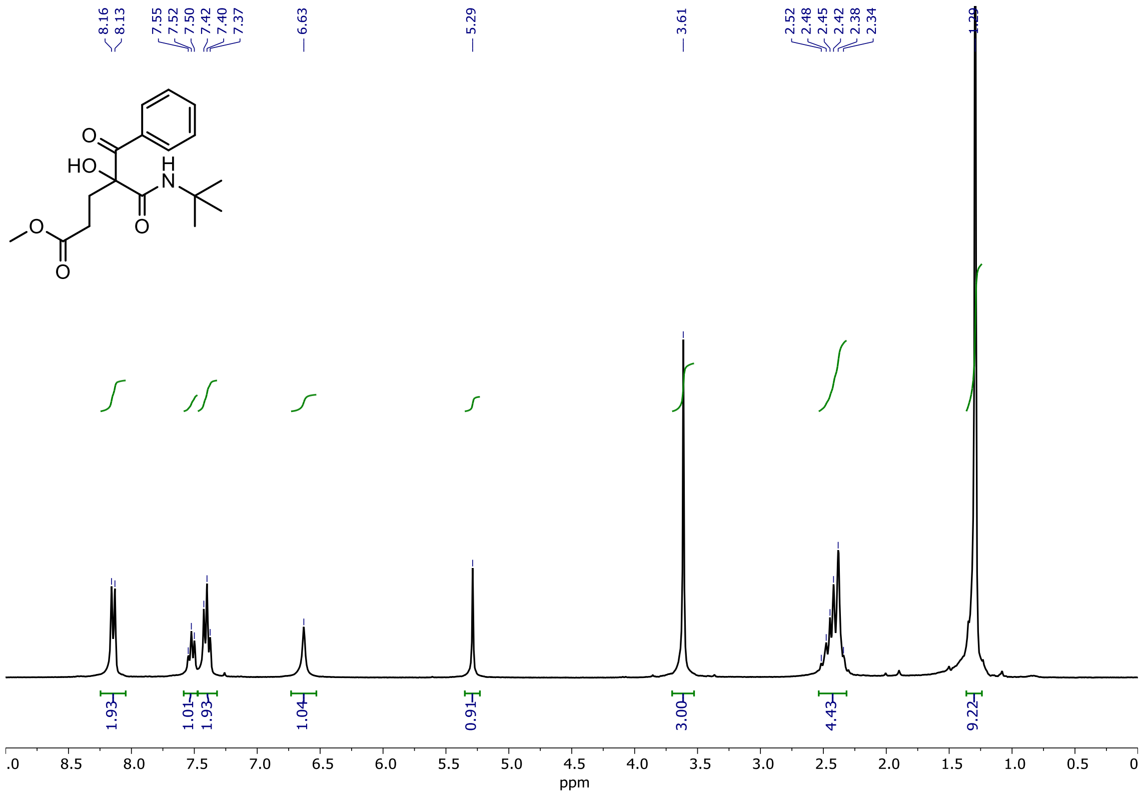

Figure 58. ^{1}H NMR spectrum of **13c** (300 MHz, $CDCl_3$).

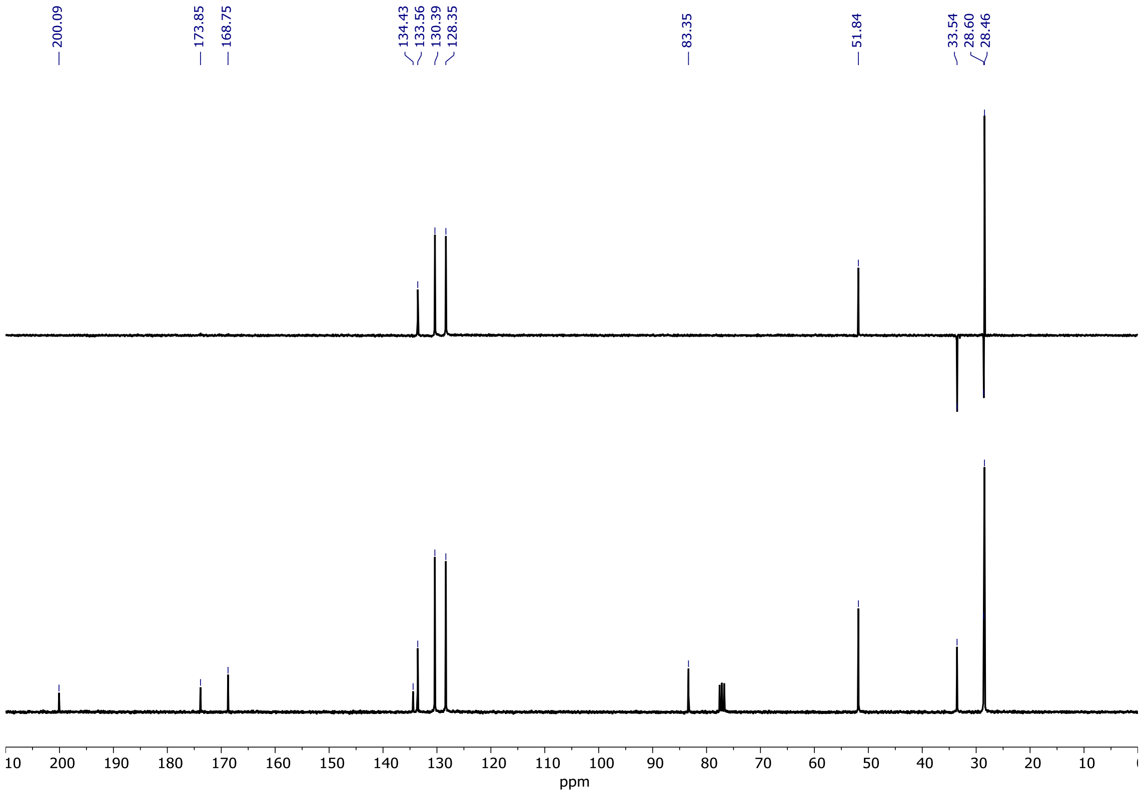

Figure 59. ^{13}C and DEPT-135 NMR spectra of **13c** (75 MHz, $CDCl_3$).

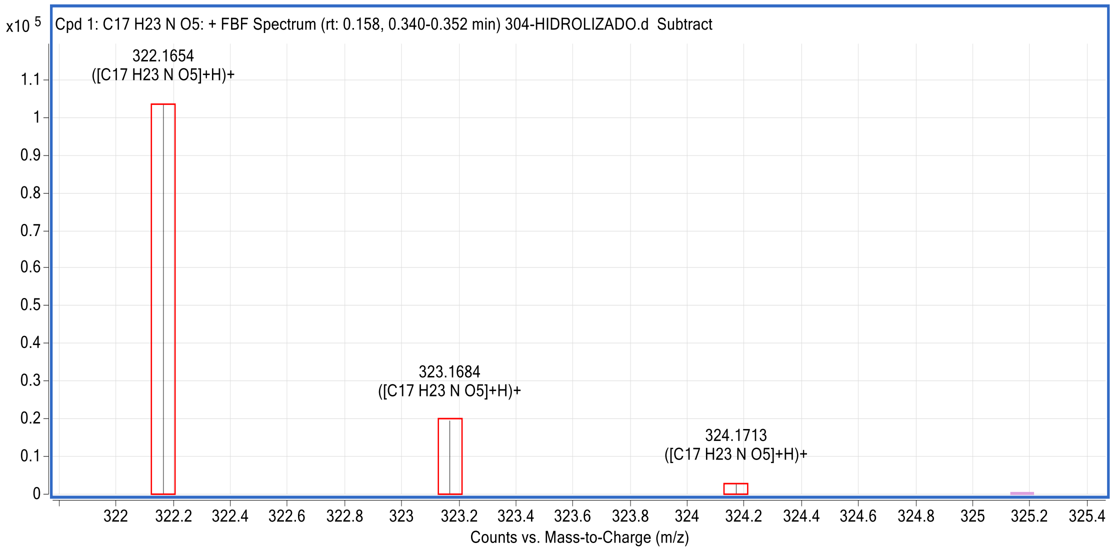

Figure 60. High-resolution mass spectrum of **13c**.

Methyl 5-(cyclohexylamino)-4-(4-fluorobenzoyl)-4-hydroxy-5-oxopentanoate (13d)

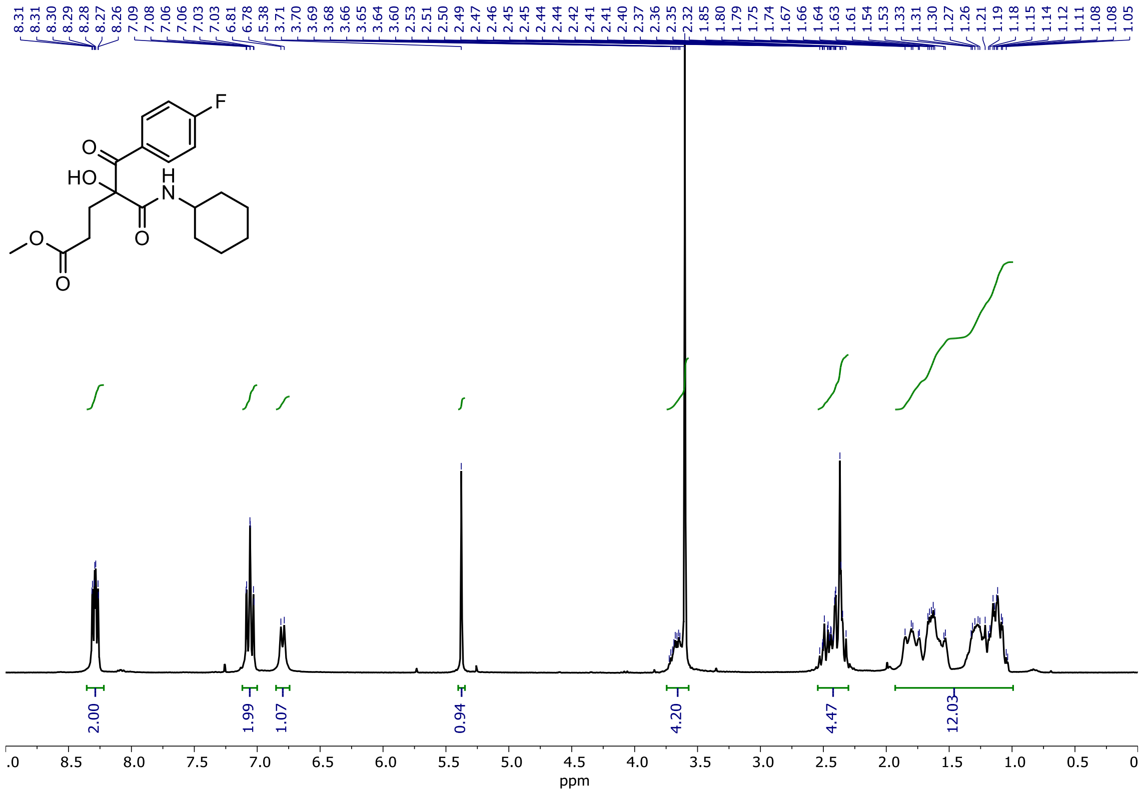

Figure 61. ^{1}H NMR spectrum of **13d** (300 MHz, $CDCl_3$).

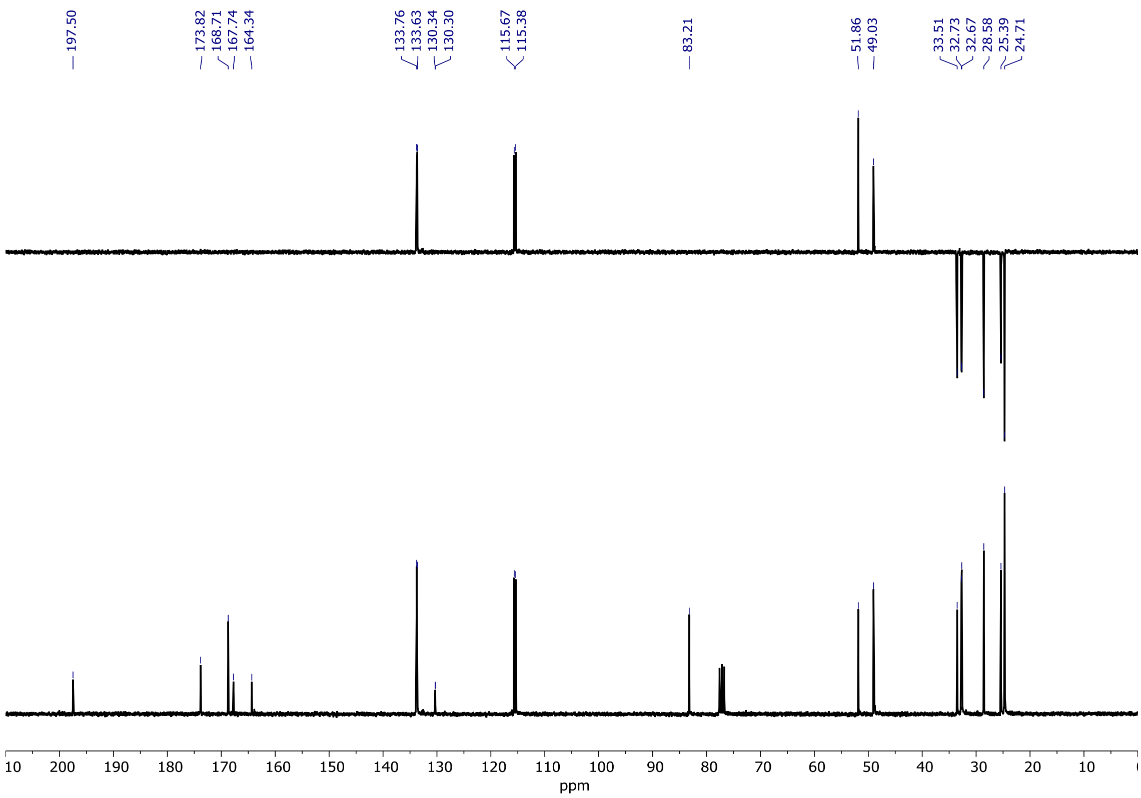

Figure 62. ^{13}C and DEPT-135 NMR spectra of **13d** (75 MHz, $CDCl_3$).

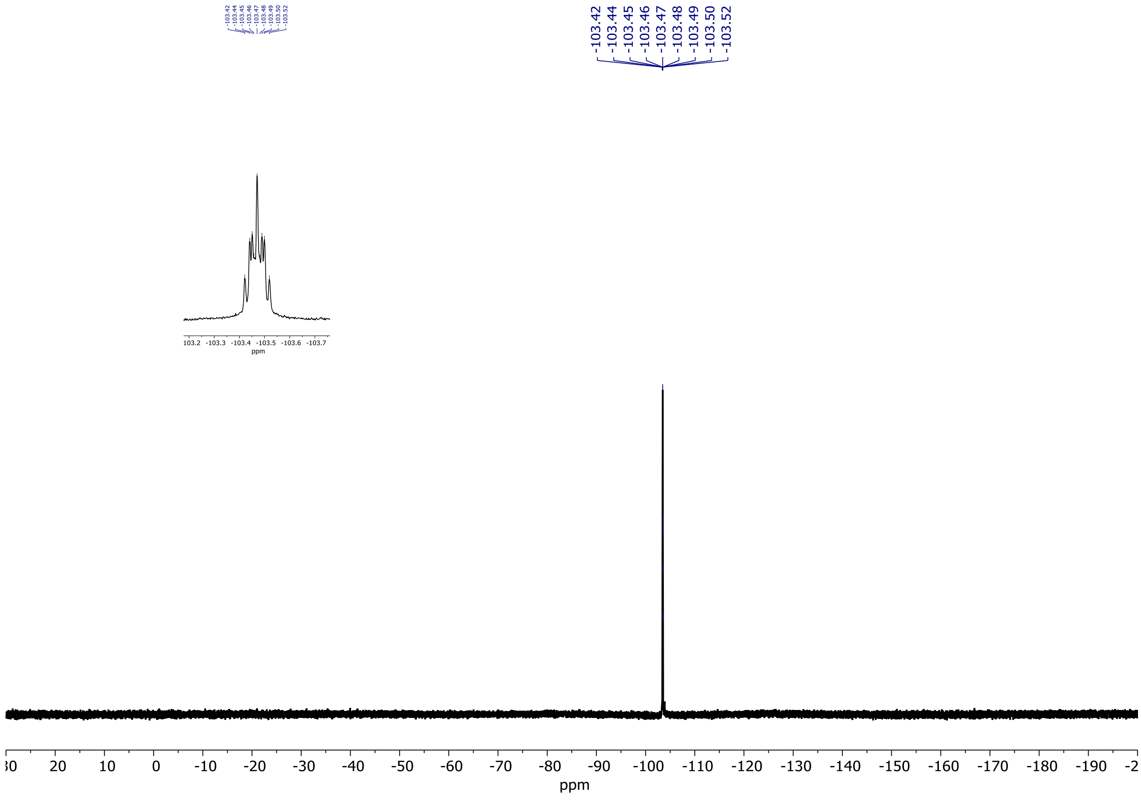

Figure 63. ^{19}F NMR spectrum of **13d** (300 MHz, $CDCl_3$).

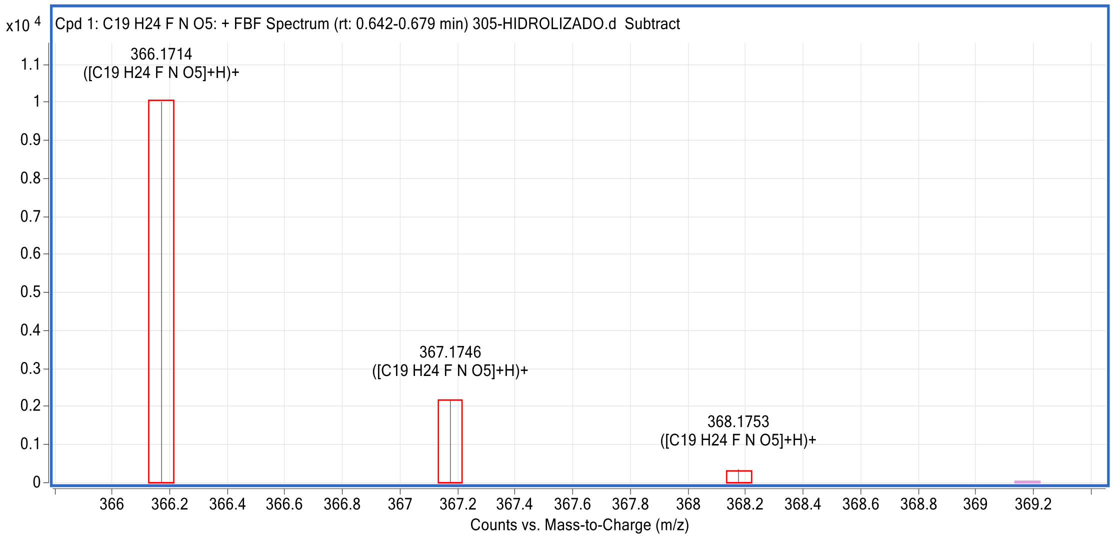

Figure 64. High-resolution mass spectrum of **13d**.

X-ray diffraction studies

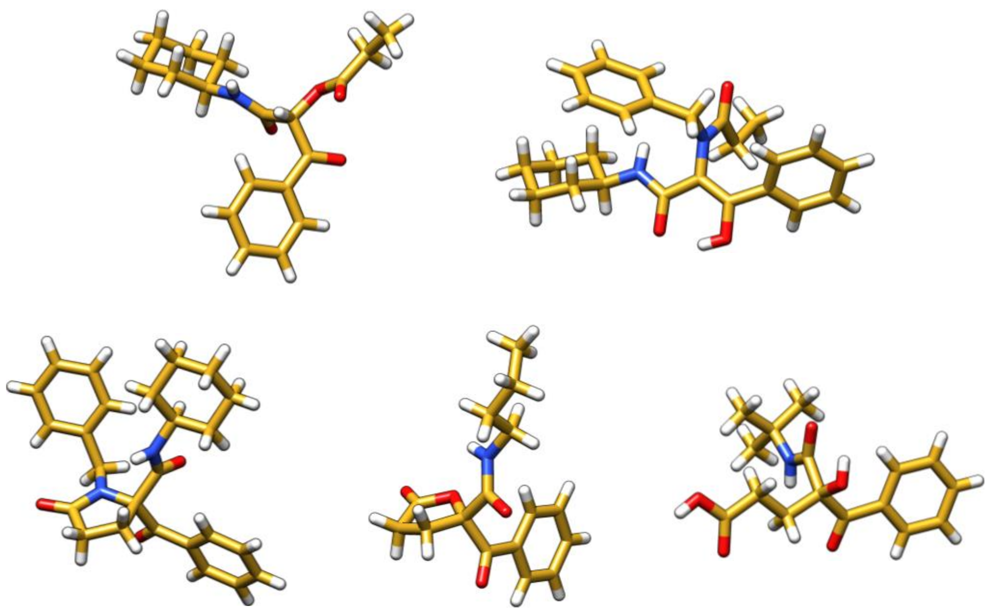

Figure 65. X-ray molecular structures of, from top to bottom and from left to right: **5**, **6**, **9**, **11b** and **12c**.

Table 1. Crystal data and refinement details for **5**, **6**, **9**, **11b** and **12c**.

	**5**	**6**	**9**	**11b**	**12c**
Empirical formula	$C_{18}H_{23}NO_4$	$C_{25}H_{30}N_2O_3$	$C_{25}H_{28}N_2O_3$	$C_{16}H_{19}NO_4$	$C_{16}H_{21}NO_5$
MW	317.37	406.51	404.49	289.32	307.34
crystal system	Ortorhombic	Triclinic	Triclinic	Monoclinic	Triclinic
space group	*Pbca*	*P*-1	*P*-1	$P2_1/c$	*P*-1
T/K	180(2)	180(2)	300(2)	180(2)	300(2)
a/Å	18.006(7)	8.2365(3)	10.213(3)	15.244(3)	8.7365(4)
b/Å	9.256(4)	10.4096(3)	11.006(3)	9.440(2)	9.1829(4)
c/Å	21.003(8)	14.2434(4)	11.207(3)	10.755(3)	10.7628(4)
α/deg	90	83.253(2)	105.448(9)	90	93.773(2)
β/deg	90	74.934(2)	114.550(8)	93.678(16)	96.241(2)
γ/deg	90	80.851(2)	91.981(9)	90	98.258(2)
V/Å^3	3500(2)	1160.51(6)	1089.2(5)	1544.5(6)	846.53(6)
F(000)	1360	436	432	616	328
Z	8	2	2	4	2
λ, Å	1.54178	1.54178	0.71073	1.54178	0.71073
D_{calc}/g cm^{-3}	1.204	1.163	1.233	1.244	1.206
μ/mm^{-1}	0.691	0.608	0.081	0.735	0.090
θ range/deg	4.87–65.18	5.18–68.23	2.22–36.50	2.90–66.56	2.86–30.58
R_{int}	0.0623	0.0742	0.0620	0.0832	0.0747
reflections measured	44626	28031	33628	10975	24160
unique reflections	2946	4229	10478	2705	5170
reflections observed	2606	2277	5728	1390	2689
GOF on F^2	1.062	1.046	1.025	1.021	1.020
$R1^a$	0.0511	0.0601	0.0643	0.0711	0.0536
$wR2^b$	0.1359	0.2378	0.1916	0.2489	0.1653
Largest ≠ peak & hole/eÅ^{-3}	0.145 and -0.191	0.235 and -0.173	0.274 and -0.194	0.376 and -0.270	0.146 and -0.158

^{a} $R1 = \sum||F_0|-|F_c||/\sum|F_0|$. ^{b} $wR2$ (all data) $= \{\sum[w(|F_0|^2-|F_c|^2)^2]/\sum[w(F_0^4)]\}^{1/2}$

Single crystals were obtained by slow evaporation of a solution of the isolated compound in diisopropyl ether (**6**), acetone (**9**), a 1:1 chloroform-hexane mixture (**5** and **11b**) or acetonitrile (**12c**).

Three dimensional X-ray data were collected on a Bruker D8 VENTURE diffractometer. Data were corrected for absorption effects using the multi-scan method (SADABS).[1] Complex scattering factors were taken from the SHELXL-2016[2] program running under the WinGX program system[3] as implemented on a Pentium® computer. The five structures were solved with Superflip[4] and refined by full-matrix least-squares on F^2. All hydrogen atoms, except those of the OH fragments of the hydroxyl and carboxyl groups of **12c**, and that of the OH fragment of the enol group of **6**, which were refined from electron density, were included in calculated positions and refined in riding mode. EXTI correction was used to complete the refinement of the structure of **6**. Refinement converged with anisotropic displacement parameters for all non-hydrogen atoms. Crystal data and details on data collection and refinement are summarized in **Table 1**. The structures were drawn with the UCSF Chimera software.[5]

1 SADABS: Krause, L.; Herbst-Irmer, R.; Sheldrick, G. M.; Stalke, D. *J. Appl. Cryst.* **2015**, *48*, 3-10.

2 SHELX-2016: Sheldrick, G. M. *Acta Cryst*. **2008**, *A64*, 112-122.

3 WinGX: Farrugia, L. J. *J. Appl. Cryst*. **1999**, *32*, 837-838.

4 SUPERFLIP: Palatinus, L.; Chapuis, G. *J. Appl. Cryst.* **2007**, *40*, 786-790.

5 UCSF Chimera: Pettersen, E. F.; Goddard, T. D.; Huang, C. C.; Couch, G. S.; Greenblatt, D. M.; Meng, E. C.; Ferrin, T. E. *J. Comput. Chem.* **2004**, *25*, 1605-1612.
